# Projected health and economic effects of a pan-tuberculosis treatment regimen: a modelling study

**DOI:** 10.1016/S2214-109X(24)00284-5

**Published:** 2024-08-16

**Authors:** Theresa S Ryckman, C Finn McQuaid, Ted Cohen, Nicolas A Menzies, Emily A Kendall

**Affiliations:** a Division of Infectious Diseases, Johns Hopkins School of Medicine, Baltimore, MD, USA; b TB Modelling Group, TB Centre and Centre for Mathematical Modelling of Infectious Diseases, Department of Infectious Disease Epidemiology, London School of Hygiene & Tropical Medicine, London, UK; c Department of Epidemiology of Microbial Diseases, Yale School of Public Health, New Haven, CT, USA; d Department of Global Health and Population and Center for Health Decision Science, Harvard T H Chan School of Public Health, Boston, MA, USA

## Abstract

**Background:**

A pan-tuberculosis regimen that could be initiated without knowledge of drug susceptibility has been proposed as an objective of tuberculosis regimen development. We modelled the health and economic benefits of such a regimen and analysed which of its features contribute most to impact and savings.

**Methods:**

We constructed a mathematical model of tuberculosis treatment parameterised with data from the published literature specific to three countries with a high tuberculosis burden (India, the Philippines, and South Africa). Our model simulated cohorts of newly diagnosed tuberculosis patients, including drug susceptibility testing if performed, regimen assignment, discontinuation, adherence, costs, and resulting outcomes of durable cure (microbiological cure without relapse), need for retreatment, or death. We compared a pan-tuberculosis regimen meeting the WHO 2023 target regimen profile against the standard of care of separate rifampicin-susceptible and rifampicin-resistant regimens. We estimated incremental cures; averted deaths, secondary cases, and costs; and prices below which a pan-tuberculosis regimen would be cost saving. We also assessed scenarios intended to describe which mechanisms of benefit from a pan-tuberculosis regimen (including improved characteristics compared with the current rifampicin-susceptible and rifampicin-resistant regimens and improved regimen assignment and retention in care for patients with rifampicin-resistant tuberculosis) would be most impactful. Results are presented as a range of means across countries with the most extreme 95% uncertainty intervals (UIs) from the three UI ranges.

**Findings:**

Compared with the standard of care, a pan-tuberculosis regimen could increase the proportion of patients durably cured after an initial treatment attempt from 69–71% (95% UI 57–80) to 75–76% (68–83), preventing 30–32% of the deaths (20–43) and 17–20% of the transmission (9–29) that occur after initial tuberculosis diagnosis. Considering savings to the health system and patients during and after the initial treatment attempt, the regimen could reduce non-drug costs by 32–42% (22–49) and would be cost saving at prices below US$170–340 (130–510). A rifamycin-containing regimen that otherwise met pan-tuberculosis targets yielded only slightly less impact, indicating that most of the benefits from a pan-tuberculosis regimen resulted from its improvements upon the rifampicin-susceptible standard of care. Eliminating non-adherence and treatment discontinuation, for example via a long-acting injectable regimen, increased health impact and savings.

**Interpretation:**

In countries with a high tuberculosis burden, a shorter, highly efficacious, safe, and tolerable regimen to treat all tuberculosis could yield substantial health improvements and savings.

**Funding:**

Bill & Melinda Gates Foundation.

## Introduction

The global burden of tuberculosis remains high, with 10·6 million new cases and 1·3 million deaths annually.^1^ Despite the development of highly efficacious regimens for treating both rifampicin-susceptible and rifampicin-resistant tuberculosis, many people with tuberculosis are not cured, contributing to a continued burden of symptoms, mortality, post-tuberculosis disability, onward transmission, and costs to both health systems and tuberculosis-affected households.^1^

Although tuberculosis treatment regimens have demonstrated high efficacy in clinical trials, their effectiveness in many programmatic settings is inadequate.^2^ Too many patients are lost to follow-up before beginning treatment,^3^, ^4^ cannot complete a full treatment course of at least 6 months, or have difficulty adhering to treatment (eg, due to daily dosing requirements, side-effects, and pill burden).^5^ Because drug susceptibility testing in many settings remains inadequate, resistance to rifampicin (4% of all incident tuberculosis globally^1^) and other drugs often goes undetected and patients are inappropriately treated.

The global tuberculosis community has called for a transformation in tuberculosis treatment, as embodied in target regimen profiles (TRPs) published by WHO in 2023.^6^ A non-rifamycin-based, shorter, safer, more tolerable, and more efficacious regimen that could treat all current drug-resistance profiles could reduce the challenges posed by current regimens. Regimens currently under evaluation for this pan-tuberculosis indication^7^ include second-line drugs and completely novel compounds, and have anticipated durations of less than 6 months (NCT05971602 and NCT06114628). The potential development of a pan-tuberculosis regimen is supported by compounds in the drug development pipeline with improved safety,^8^ potency,^9^ and pharmacokinetic properties, and new drug classes^10^ for which population-level resistance is expected to be minimal.

**Research in context**

**Evidence before this study**

This study was informed by the WHO updated target regimen profiles (TRPs) for tuberculosis treatment. The 2023 TRP document describes minimal and optimal characteristics for future rifampicin-susceptible, rifampicin-resistant, and universally indicated pan-tuberculosis regimens to meet. The document also describes modelling that was conducted to inform the rifampicin-susceptible and rifampicin-resistant regimen targets. However, no such modelling was conducted to inform the pan-tuberculosis targets. To identify studies evaluating the potential population-level health benefits or cost implications of such a pan-tuberculosis regimen, we searched PubMed using the following search terms: (tuberculosis OR TB) AND (“pan-TB” OR “pan-tuberculosis” OR universal) AND (treatment OR therapeutic OR regimen). All of these search terms were restricted to a title and abstract search, with the exception of “pan-TB” and “pan-tuberculosis”. We performed the search on Feb 28, 2024, and restricted our search to human studies published on or after Jan 1, 2010. No restriction on language was made. This search yielded 314 published studies, of which four contained estimates of the health or economic impact of universally indicated tuberculosis regimens compared to a standard of care. Studies varied in the extent to which the pan-tuberculosis regimen being modelled improved upon existing standards of care and thus varied in terms of projected health impact. All four studies compared a pan-tuberculosis scenario against previous rifampicin-resistant standards of care that were longer, less safe and tolerable, and less effective, and were conducted in an era when the coverage of rifampicin susceptibility testing was lower than it is now.

**Added value of this study**

This study builds on modelling conducted for non-pan-tuberculosis regimens as part of the 2023 TRP development process and adds to the existing literature by modelling a pan-tuberculosis regimen with more forward-looking characteristics (reflecting the more recent and ambitious TRP), updated standard-of-care regimens (which have improved particularly for rifampicin-resistant tuberculosis), and updated evidence on rifampicin susceptibility testing under the standard-of-care scenario (which has increased over the past decade). We not only estimated the potential impact and savings from pan-tuberculosis regimen scale-up (against standards of care), but also evaluated the extent to which the impact and savings are driven by the regimen's universal indication, rather than by other mechanisms of benefit (such as improved efficacy compared with current rifampicin-susceptible or rifampicin-resistant regimens).

**Implications of all the available evidence**

A tuberculosis treatment regimen with improved characteristics compared with standard-of-care regimens, and that could be used to treat all tuberculosis without drug susceptibility testing, could yield substantial health benefits and non-drug cost savings. Our results suggest that, in light of recent and ongoing improvements to the diagnosis and treatment of rifampicin-resistant tuberculosis, much of this impact could come from the extent to which this regimen improves rifampicin-susceptible tuberculosis outcomes. However, the universal indication inherent to a pan-tuberculosis regimen is expected to augment this impact and could yield improvements particularly for patients with rifampicin-resistant tuberculosis.

The investment case for pursuing a pan-tuberculosis regimen depends on the expected health impact, cost-effectiveness, and affordability of such a regimen. Developers should also understand which mechanisms of potential benefit from a pan-tuberculosis regimen are most influential and which characteristics are most crucial in the current tuberculosis care context. We used mathematical modelling to explore the health and economic implications of a pan-tuberculosis regimen and analysed which of its features contribute most to impact and savings.

## Methods

### Study overview

Building on a previous analysis of improved rifampicin-susceptible and rifampicin-resistant tuberculosis regimens,^11^ we developed a cohort model of clinical outcomes among people diagnosed with tuberculosis, which we combined with a simplified model of onward transmission and an ingredients-based costing approach. We used this model to evaluate the health and economic consequences of introducing a hypothetical pan-tuberculosis regimen, relative to current standards of care. We also evaluated several isolated improvements to the standards of care, intended to describe how each of four distinct benefits of a pan-tuberculosis regimen contribute to health improvements and cost savings. All model scenarios are described in table 1.

**Table 1:** Regimen scenarios

	**Rationale**	**Separate RR tuberculosis regimen?**	**Efficacy**	**Duration**	**Adherence**	**Forgiveness**	**Pretreatment loss to follow-up**	**Rifampicin DST**
**Primary scenarios for comparison**								
Standard of care	Simplified representation of current treatment (mostly HRZE and BPaL[M])	Yes	Standard of care	6 months	Standard of care levels	Standard of care	Higher for RR tuberculosis than RS tuberculosis	Standard of care levels
Pan-tuberculosis regimen	Oral regimen meeting the WHO pan-tuberculosis minimal target regimen profile	No	Equivalent to RS standard of care	3·5 months	Improved	Improved	Same for RS and RR tuberculosis	NA
**Isolated-improvement scenarios (isolating mechanisms of benefit of the oral pan-tuberculosis regimen)**								
Improved RS tuberculosis regimen characteristics	Isolates the RS tuberculosis treatment improvement benefit of a pan-tuberculosis regimen	Yes	Standard of care	2 months for RS tuberculosis; 6 months for RR tuberculosis	Improved for RS tuberculosis only	Improved for RS tuberculosis only	Higher for RR tuberculosis than RS tuberculosis	Standard of care levels
Improved RR tuberculosis regimen characteristics	Isolates the RR tuberculosis treatment improvement benefit of a pan-tuberculosis regimen	Yes	Equivalent to RS standard of care	6 months for RS tuberculosis; 2 months for RR tuberculosis	Improved for RR tuberculosis only	Improved for RR tuberculosis only	Higher for RR tuberculosis than RS tuberculosis	Standard of care levels
Improved retention in care for RR-tuberculosis	Isolates the retention improvement benefit of a pan-tuberculosis regimen	Yes	Standard of care	6 months	Same as standard of care	Same as standard of care	Same for RS and RR tuberculosis	Standard of care levels
Improved RR tuberculosis regimen assignment	Isolates the improved regimen assignment benefit of a pan-tuberculosis regimen	Yes	Standard of care	6 months	Same as standard of care	Same as standard of care	Higher for RR tuberculosis than RS tuberculosis	Higher DST coverage
**Alternative pan-tuberculosis scenarios**								
Pan-tuberculosis oral regimen with confirmatory DST	Adds DST to confirm susceptibility to drugs in the regimen	No*	Equivalent to RS standard of care	3·5 months	Improved	Improved	Same for RS and RR tuberculosis	Only for novel-drug-resistant tuberculosis*
Pan-tuberculosis one-time long-acting injectable regimen	Optimises duration and adherence	No	Equivalent to RS standard of care	NA	Perfect	NA	Same for RS and RR tuberculosis	NA

Comparisons of efficacy, adherence, forgiveness, pretreatment loss to follow-up, and rifampicin DST are against the standard of care. Additional details are presented in the appendix (pp 3–5). RR=rifampicin-resistant. DST=drug susceptibility testing. HRZE=6 months of isoniazid, rifampicin, pyrazinamide, and ethambutol (the standard of care for treating RS tuberculosis). BPaL(M)=6 months of bedaquiline, pretomanid, linezolid, and moxifloxacin (the standard of care for treating RR tuberculosis). RS=rifampicin-susceptible. NA=not applicable.

* People with detected novel-drug resistance would receive HRZE if RS or an individualised regimen if RR; all others would continue to receive the pan-tuberculosis regimen.

The model was constructed in R version 4.2.2 and parameterised with epidemiological and cost data specific to three countries (India, the Philippines, and South Africa) selected for geographical, drug-resistance, and income diversity. Outcomes of interest included durable cures (ie, microbiological cure without relapse), tuberculosis mortality, secondary cases, and the prices below which a pan-tuberculosis regimen would be cost-saving or cost-effective compared with standard of care. Although adoption of new regimens will be gradual and context-dependent, we modelled immediate 100% uptake to isolate regimen effects.

### Patient cohort model

Our state-transition model tracked annual cohorts of treatment-naive adults newly diagnosed with pulmonary tuberculosis (hereafter referred to as patients) over a 10-year horizon. We modelled population-level resistance trends, a pretreatment phase (including regimen assignment and initiation), an on-treatment phase (including adherence, discontinuation, and adverse events), and subsequent health outcomes (including retreatments, secondary cases, and mortality). Model parameters are described in the appendix (pp 3–5).

To evaluate population-level resistance trends, we modelled four resistance phenotypes: resistance to rifampicin only, resistance to novel drugs (ie, those in the pan-tuberculosis regimen) only, resistance to both rifampicin and novel drugs, or resistance to neither. We assumed that the pan-tuberculosis regimen would draw on drug classes already used for treating rifampicin-resistant tuberculosis, such that patients with novel-drug-resistant tuberculosis would have resistance to both the pan-tuberculosis and rifampicin-resistant tuberculosis standard-of-care regimens. At baseline, we estimated the prevalence of novel-drug resistance as 0·2% among rifampicin-susceptible tuberculosis and 1–4% among rifampicin-resistant tuberculosis;^1^, ^12^, ^13^ increases in resistance over time were modelled as proportional to corresponding regimen usage (appendix pp 6–9).

The pretreatment model captured drug susceptibility testing, regimen assignment and initiation, and pretreatment losses to follow-up (appendix pp 6–10). Under the standard-of-care scenario, coverage of rifampicin drug susceptibility testing remained constant,^1^ and testing for novel-drug resistance among those with detected rifampicin resistance gradually increased. Patients without detected rifampicin resistance were assigned to the rifampicin-susceptible tuberculosis standard of care, which consisted mostly of the 6-month HRZE regimen (isoniazid, rifampicin, pyrazinamide, and ethambutol). Due to low uptake of the 4HPMZ regimen (4-month isoniazid, rifapentine, moxifloxacin, and pyrazinamide) for rifampicin-susceptible tuberculosis, we considered this alternative in a sensitivity analysis.^1^

As the standard of care for patients with detected rifampicin resistance, we selected BPaL(M) (6 months of bedaquiline, pretomanid, linezolid, and, for most patients, moxifloxacin) based on WHO recommendations.^14^ Although countries are in various stages of adopting BPaL(M), this choice allows the analysis to be conservative in its estimates of the benefits of improved regimens, because BPaL(M) represents a substantial improvement over previous rifampicin-resistant tuberculosis regimens. Average outcomes and costs were modified by isoniazid resistance in rifampicin-susceptible tuberculosis regimens and by fluoroquinolone resistance in rifampicin-resistant regimens (appendix p 7). Patients with detected resistance to both rifampicin and novel drugs were assigned to individualised regimens (ie, less efficacious and tolerable, longer-duration, and higher-cost regimens that might be indicated for patients with extensively drug-resistant tuberculosis, under current definitions).

Under the pan-tuberculosis scenario, no drug susceptibility testing was conducted and all new patients were assigned to the pan-tuberculosis regimen, with pretreatment loss to follow-up similar to that for patients assigned to the rifampicin-susceptible tuberculosis standard of care. In the standard-of-care scenario, pretreatment loss to follow-up was assumed to be greater for those assigned to rifampicin-resistant or individualised regimens, due to delays incurred in initiating these separate treatment pathways.^1^, ^3^, ^4^

In the on-treatment model, treatment was modelled as resulting in either durable cure, failure to cure, or death. Failure to cure encompassed failures during treatment, successes with subsequent relapse, and some losses to follow-up during treatment. Death during treatment was modelled as a fixed proportion among those not durably cured.^1^

The probability of durable cure depended on regimen efficacy, duration, ease of adherence, forgiveness (ie, the extent to which durable cure occurs despite missed doses), and resistance (appendix p 3). Efficacy was defined as the proportion of patients curable by the regimen under optimal conditions of perfect adherence, retention in care, and complete initial regimen susceptibility; for the standard of care, efficacy estimates were based on clinical trial data.^15^, ^16^ Starting from each regimen's efficacy, the probability of cure was adjusted downward for resistance to drugs in the regimen, early treatment discontinuation (appendix p 11), and missed doses while on treatment. Discontinuation with standard-of-care regimens was based on programmatic data, and adherence was based on control groups in trials of adherence-improving interventions. The impact of adherence varied by regimen forgiveness;^17^ for more forgiving regimens, more doses could be intermittently missed without affecting the probability of cure.

The hypothetical pan-tuberculosis regimen was informed by the WHO minimal TRP and ongoing regimen development efforts (table 1).^6^ In particular, the regimen was modelled as easier to adhere to, at least as forgiving, of shorter duration (3·5 months), and as efficacious and safe as HRZE, and consisting of drugs with a lower population-wide prevalence of resistance than rifampicin. We varied these characteristics in sensitivity analyses.

We modelled people who did not initiate treatment or who failed treatment as experiencing further active tuberculosis, with contributions to transmission and competing risks of case fatality and tuberculosis detection. Previously treated patients who were subsequently retreated were modelled as experiencing a lower probability of cure than treatment-naive patients. Non-cured patients who remained alive were assumed to generate, on average, one secondary case of tuberculosis disease, after a simulated serial interval (appendix p 12). Undiagnosed secondary cases were also assumed to face competing risks of fatality and detection; detected secondary cases were assumed to experience the same outcomes as primary cases. Estimates of mortality thus capture all tuberculosis-related deaths that are avertible at the point of diagnosis, including post-diagnosis deaths among index patients and deaths arising from transmission after index patient diagnosis.

### Outcomes

Under each scenario, we estimated durable cures (referred to as initial cures if cured after one round of treatment, and eventual cures if cured after one or more rounds), avertible tuberculosis deaths, and avertible secondary cases, each relative to the number of new tuberculosis diagnoses. These ratios are presented as annual averages over 10 years. Estimates of the cumulative effect of the pan-tuberculosis regimen accounted for both improved outcomes among the average patient and reductions in numbers of patients over time, based on a model extension that estimated dynamic reductions in incidence resulting from regimen improvements (appendix p 13).

Costs were estimated using an ingredients-based approach that multiplied fixed country-specific unit costs by the quantities of inputs required under each scenario (appendix pp 14–19). Because the price of a future pan-tuberculosis regimen is unknown, the primary cost outcome was the price below which the regimen would be cost-saving compared with the standard of care, under a societal perspective that considered both health-system and patient-borne costs. The prices at which a pan-tuberculosis regimen would be cost saving were estimated by calculating the difference in total costs per patient treated between the pan-tuberculosis scenario (excluding unknown pan-tuberculosis drug costs) and the comparator scenario (including the costs of standard-of-care regimens). Short-term prices considered only costs accrued before and during a patient's treatment course, and medium-term prices added savings from averted retreatments and secondary cases. All costs were calculated in 2021 US dollars (US$; appendix p 14).

In secondary analyses, we estimated the prices at which a pan-tuberculosis regimen would be cost effective, accounting for both costs and health improvements (as disability-adjusted life-years [DALYs]; appendix p 20). Tuberculosis mortality was converted to life-years based on country-specific life expectancies and age distributions of incident tuberculosis,^1^, ^18^ and costs and DALYs were discounted at 3% annually. DALYs also captured non-fatal health losses from tuberculosis illness, adverse events, and post-tuberculosis mortality and disability.^19^ The cost-effectiveness analysis used country-specific willingness-to-pay thresholds^20^ and adhered to the Consolidated Health Economic Evaluation Reporting Standards 2022 statement (appendix p 21).

### Statistical analysis

In the main analysis, we propagated uncertainty in model parameters by running the model for 10 000 parameter sets sampled from uncertainty distributions. Results are presented as means and uncertainty intervals (UIs; the 2·5th and 97·5th percentiles of modelled outputs) for a given country. Summaries across countries are presented as a range of means and a range between the most extreme UI endpoints.

In sensitivity analyses, we considered two alternatives to the main pan-tuberculosis scenario. To reflect a scenario in which the pan-tuberculosis regimen is only used empirically until confirmation of drug susceptibility, we modelled use of novel-drug susceptibility testing with the pan-tuberculosis regimen (in which those with detected novel-drug resistance receive either the rifampicin-susceptible tuberculosis standard of care or an individualised regimen depending on rifampicin susceptibility). To estimate the impact of optimising adherence and eliminating discontinuation during treatment, we modelled a pan-tuberculosis regimen that was delivered as a single, sustained-release dose (ie, long-acting injectable).^21^, ^22^

Additionally, we conducted one-way sensitivity analyses on pan-tuberculosis regimen characteristics, and sensitivity analyses exploring specific assumptions about the standards of care and the prevalence of novel-drug resistance (appendix pp 23–24).

### Role of the funding source

Employees of the Bill & Melinda Gates Foundation provided initial input on scope, suggesting to estimate the health, economic, and incidence impact of both oral and long-acting injectable pan-tuberculosis regimens resembling the 2023 WHO TRP. Based on pan-tuberculosis regimens currently under development, they suggested basing novel-drug resistance on phenotypic bedaquiline resistance. They were not involved in final decisions about model parameters or regimen scenarios and had no role in data collection, data analysis, data interpretation, or writing of the report.

## Results

Under the standard-of-care scenario, 69–71% of patients (95% UI 57–80) diagnosed with tuberculosis in each country were projected to be durably cured by an initial treatment attempt; this proportion increased to 82–86% (71–92) after retreatment (table 2). Per 100 diagnosed patients, we projected 10–16 (6–26) deaths (including among relapses and secondary cases) and 7–8 (4–12) secondary cases, with variation across countries driven primarily by variation in case detection and case-fatality proportions.

**Table 2:** Estimated health outcomes and costs over 10 years under the standard-of-care scenario

		**India**	**Philippines**	**South Africa**
Initial durable cures per 100 index diagnoses		70·5 (58·3–79·7)	70·7 (58·4–80·0)	68·9 (57·0–77·9)
Eventual durable cures* per 100 index diagnoses		83·0 (71·9–90·0)	85·6 (74·9–92·2)	82·2 (71·4–89·3)
Index patient deaths per 100 index diagnoses		10·3 (6·2–16·8)	6·3 (3·5–10·9)	9·9 (6·0–15·9)
Total deaths† per 100 index diagnoses		16·0 (9·4–26·2)	10·3 (5·6–17·8)	15·5 (9·2–25·0)
Secondary cases per 100 index diagnoses		7·6 (4·7–10·7)	8·1 (4·5–11·8)	6·9 (4·1–10·0)
Costs per person, US$				
	Short-term, health system	140 (110–160)	130 (110–140)	260 (220–310)
	Short-term, societal	360 (250–490)	410 (270–620)	600 (450–860)
	Medium-term, societal	470 (330–650)	570 (370–870)	800 (600–1150)

Values in parentheses are 95% uncertainty intervals. All health outcomes are presented as annual averages over the 10-year model horizon. Short-term costs were calculated only during a patient's primary treatment course, whereas medium-term costs include the costs of retreatment and treatment of secondary cases.

* Includes those cured through retreatment.

† Includes deaths among secondary cases (that are attributable to transmission after index patient diagnosis).

**Figure 1:**
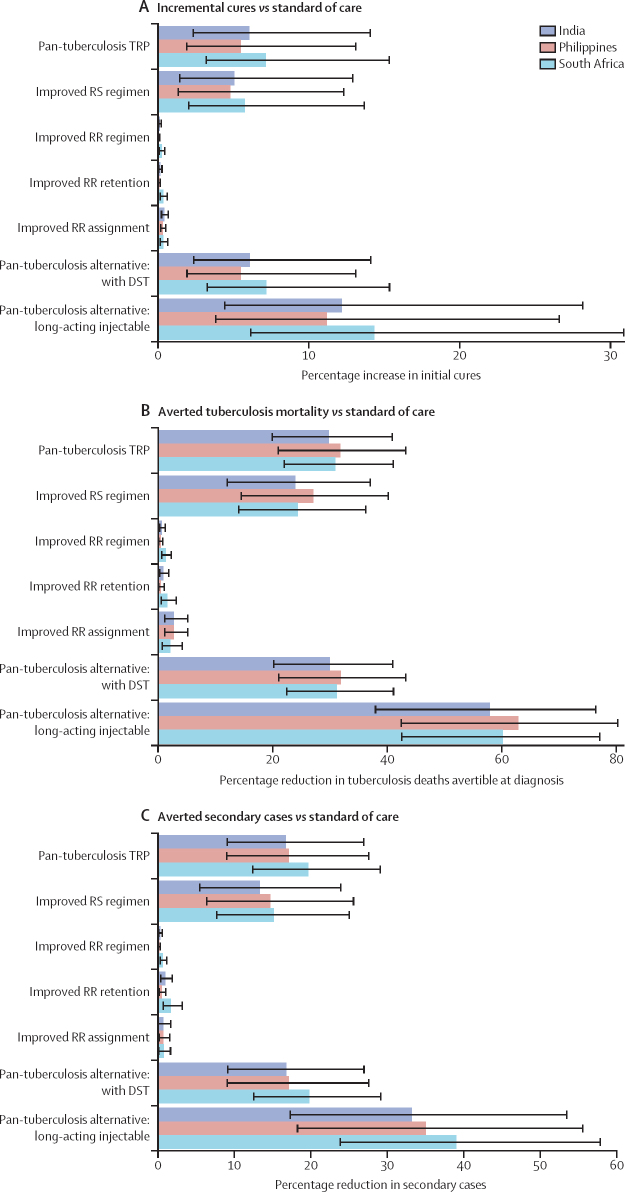
Incremental health outcomes under a pan-tuberculosis regimen and isolated-improvement scenarios compared with standard of care The figure shows the percentage increase in the average proportion of newly diagnosed patients durably cured of tuberculosis after initial treatment (A), the percentage reduction in cumulative tuberculosis deaths arising after diagnosis, including those averted through prevention of onward transmission (B), and the percentage reduction in cumulative secondary cases (C) in the pan-tuberculosis and isolated-improvement scenarios relative to the standard-of-care scenario. The coloured bars indicate means and black error bars indicate 95% uncertainty intervals. All outcomes are presented over 10 years. DST=drug susceptibility testing. RR=rifampicin-resistant. RS=rifampicin-susceptible. TRP=target regimen profile.

Assigning all patients to a pan-tuberculosis regimen meeting the WHO minimal TRP increased initial cures to 75–76% (95% UI 68–83) and eventual cures to 88–90% (82–94). This prevented 30–32% of treatment-avertible tuberculosis deaths (20–43) and 17–20% of avertible secondary cases (9–29; figure 1). Mirroring these projections, the regimen was estimated to reduce annual tuberculosis incidence by 4–5% (2–9) after 10 years (appendix pp 30–32).

An improved rifampicin-susceptible tuberculosis regimen (rifamycin-containing, but with the duration, adherence, and forgiveness advantages of the pan-tuberculosis regimen) was estimated to have nearly as much health impact as the pan-tuberculosis regimen: 24–27% fewer tuberculosis deaths (95% UI 12–40) and 13–15% fewer secondary cases (5–26). The other isolated-improvement scenarios (improved rifampicin-resistant tuberculosis regimen, patient retention, or regimen assignment) yielded much smaller improvements in population-wide health outcomes: a 5% or smaller decline in mortality and a 3% or smaller decline in secondary cases. Compared directly with the improved rifampicin-susceptible tuberculosis regimen scenario, the pan-tuberculosis scenario decreased deaths and secondary cases by 6–9% (3–13) and 3–5% (2–8), respectively (appendix pp 30–35).

Under the standard-of-care scenario, per-person health-system costs during a patient's treatment course were $140 (95% UI 110–160) in India, $130 (110–140) in the Philippines, and $260 (220–310) in South Africa, where unit costs were highest (table 2). Laboratory tests, outpatient visits, and drugs made up the greatest share of short-term health-system costs (appendix p 36). Incorporating patient-borne non-medical and indirect costs more than doubled total costs. Adding the costs of retreatments and treatment of secondary cases further increased per-person costs to $470 (330–650) in India, $570 (370–870) in the Philippines, and $800 (600–1150) in South Africa.

**Figure 2:**
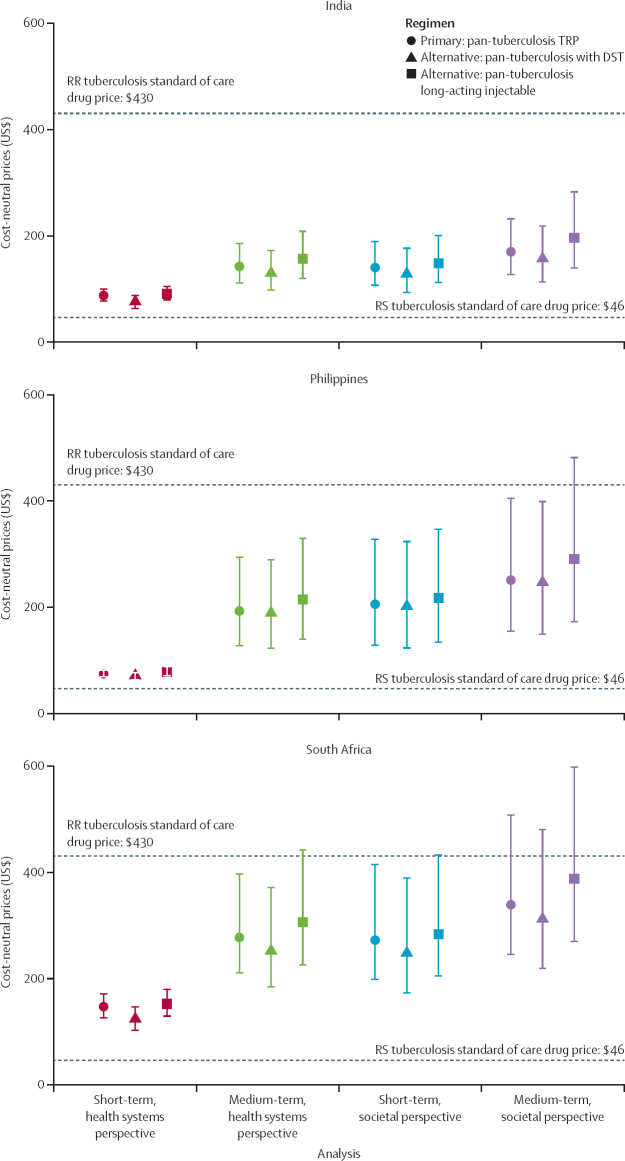
Cost-saving prices of a pan-tuberculosis regimen The figure shows the prices below which the pan-tuberculosis regimen would be cost-saving compared with the standard of care. Markers indicate means and error bars indicate 95% uncertainty intervals. Results are presented in 2021 US dollars. Current drug prices of the rifampicin-susceptible and rifampicin-resistant standards of care, which were assumed not to vary by country, are shown as dashed horizontal lines on each plot. DST=drug susceptibility testing. RR=rifampicin-resistant. RS=rifampicin-susceptible. TRP=target regimen profile.

A pan-tuberculosis regimen reduced non-drug costs for patients and the health system in the short term (primarily from shorter regimen duration) and the medium term (from averted retreatments and transmission) by 32–42% (95% UI 22–49; appendix p 36). Due to these savings, the regimen could be priced higher than the current rifampicin-susceptible tuberculosis standard of care (HRZE; $46) and still be cost saving under all time horizons and perspectives considered. From a societal perspective, medium-term cost-saving price thresholds were $170 (130–230) in India, $250 (150–400) in the Philippines, and $340 (250–510) in South Africa (figure 2). Cost-saving prices were lower when considering only short-term health-system costs.

Of the four isolated-improvement scenarios, the greatest savings were with an improved rifampicin-susceptible tuberculosis regimen. Cost-saving prices for pan-tuberculosis regimens thus depended heavily on the comparator rifampicin-susceptible tuberculosis regimen, and were far lower in a scenario in which a pan-tuberculosis regimen was introduced after the scale-up of an improved rifampicin-susceptible tuberculosis regimen priced the same as HRZE (appendix pp 37–39).

Cost-effective prices were substantially higher ($620–4260) than cost-neutral prices, reflecting both monetary savings and DALYs averted from improved regimens, and they varied with willingness to pay (appendix pp 40–42).

The 10-year impact of the pan-tuberculosis regimen changed minimally when testing was added to confirm susceptibility, but cost-saving prices were lower than in the main analysis. Eliminating non-adherence and discontinuation during treatment via a long-acting injectable pan-tuberculosis regimen improved outcomes more than the oral regimen and increased cost-saving prices.

A one-way sensitivity analysis on pan-tuberculosis regimen characteristics showed that health outcomes were most sensitive to efficacy, adherence, and forgiveness, whereas savings were most sensitive to duration and safety (appendix pp 44–47). In additional sensitivity analyses (appendix pp 49–57), health outcomes varied somewhat with the magnitude of gap between clinical trial efficacy and programmatic outcomes under the standard-of-care scenario, and the extent to which this difference shrank with the pan-tuberculosis regimen. Savings (and thus cost-saving prices) were lower when 4HPMZ was modelled as the rifampicin-susceptible tuberculosis standard of care. Results were insensitive to simultaneously doubling the baseline prevalence of resistance to the pan-tuberculosis regimen and its rate of emergence over 10 years. The relative impacts of the pan-tuberculosis and isolated-improvement scenarios were qualitatively unchanged in all sensitivity analyses.

## Discussion

A pan-tuberculosis regimen meeting internationally accepted targets could have substantial impact in three countries with high tuberculosis burden, with immediate national uptake resulting in 30–32% fewer tuberculosis deaths (among deaths avertible at the point of diagnosis) and 32–42% lower non-drug treatment costs, over 10 years. The regimen would need to be priced no more than 1·5 to three times higher than HRZE to be cost saving compared with the standards of care under a short-term health-systems perspective. Adding patient-borne costs or the costs of retreatments and secondary cases (or both) increased cost-saving price thresholds to between three and five times higher than HRZE in India and the Philippines and six to seven times higher in South Africa.

The isolated-improvement scenarios demonstrated that most of the predicted impact and savings of a pan-tuberculosis regimen came not from its universal indication, but rather from its superiority over HRZE as an rifampicin-susceptible tuberculosis regimen, in terms of duration, adherence, and forgiveness. Especially given recent improvements to the rifampicin-resistant tuberculosis standard of care, it has become more crucial for a pan-tuberculosis regimen to improve outcomes, and be priced competitively, relative to the rifampicin-susceptible tuberculosis standard of care.^23^, ^24^ As a result, if a rifamycin-containing regimen could reach other pan-tuberculosis targets, the case for developing and adopting a pan-tuberculosis regimen becomes less certain and more dependent on specific operational and drug-resistance-related characteristics of a regimen.

In an idealised conceptualisation that eliminated non-adherence and non-completion, a long-acting injectable pan-tuberculosis regimen almost doubled the oral regimen's impact on cases and deaths. In the context of existing rifampicin-susceptible tuberculosis and rifampicin-resistant tuberculosis regimens that are already highly efficacious under optimal conditions, this finding illustrates the importance of reducing the gap between efficacy and effectiveness by not only improving regimen characteristics (as modelled here), but also by strengthening tuberculosis programmes and improving patient experiences, such as through nutritional, social, or other patient-focused support during treatment.

Although the prevalence of rifampicin-resistant tuberculosis in the modelled countries reflects global averages, our analysis has limited generalisability to settings with very high rifampicin-resistant tuberculosis prevalence (ie, >10%). Emergence of extensively drug-resistant tuberculosis^25^ could limit the feasibility of the simplified test-and-treat strategy in these settings, at least with current candidate regimens that contain existing second-line drug classes (NCT05971602 and NCT06114628). Furthermore, although simplified treatment strategies resulting from an improved and universally indicated regimen could encourage increased tuberculosis detection—for example, facilitating development and use of simpler point-of-care diagnostics^26^ or altering the risk-benefit calculation for treating early disease^27^—such benefits were not considered here.

Other limitations include uncertainty about changes in drug resistance prevalence, drug susceptibility testing practices, standards of care, care cascades, and the impact of advances in prevention, diagnostics, and vaccines on overall disease burden by the time a pan-tuberculosis regimen becomes available; this time period is highly uncertain but could be long. Evidence on the prevalence, acquisition, and consequences of bedaquiline resistance is still emerging, and might also have limited applicability to future pan-tuberculosis regimens, which could include next-generation diarylquinolines or other drug classes. Although parameters related to novel-drug resistance had little effect within the relatively short horizon modelled, resistance is expected to become more problematic with longer-term usage, necessitating proactive strategies to minimise, monitor, and respond to emerging resistance (for example, by reinstituting universal drug susceptibility testing) to preserve regimen durability. Without such strategies, amplification of and selection for resistance could eventually outweigh the benefits of a pan-tuberculosis regimen.^28^ Other potential downsides to a pan-tuberculosis strategy include that the one-size-fits-all approach could lead to poorer outcomes for patients with novel-drug-resistant tuberculosis and fails to account for patient preferences.

The characteristics of a pan-tuberculosis regimen in reality, although explored in sensitivity analyses, are unlikely to match those modelled here, which were based on published targets. Future analyses could incorporate evidence on the characteristics (including resistance-related characteristics) and costs of specific regimen candidates, and adherence and forgiveness under current regimens (which were based on a small body of evidence from HRZE), as such evidence emerges from clinical trials and operational research. Although the pan-tuberculosis regimens modelled here are ambitious, findings from a 2023 clinical trial point towards the possibility of regimens that are short and efficacious, including rifamycin-free regimens.^29^

We assumed immediate scale-up of new regimens under all scenarios. Impact and savings will be proportional to uptake over time, which could vary due to cost, access, acceptability, and other factors. Also, our simple transmission modelling approach yielded incidence estimates which depend heavily on assumptions regarding the transmission potential of individuals who are not cured after diagnosis. Finally, all mathematical models are necessarily simplifications of the real world and subject to limitations imposed by assumptions inherent in the model's structure.

A previous modelling analysis of a pan-tuberculosis regimen in India estimated greater incidence reductions and cost savings than in the current study.^24^ Improvements in the diagnosis of rifampicin resistance (scale-up of rapid molecular testing) and its treatment (BPaL[M] adoption) since the previous publication probably account for much of this difference. Assumptions about loss to follow-up also differed between models. Our analysis adds to the literature by comparing a pan-tuberculosis regimen against the latest standards of care and isolating which of its attributes would be most beneficial. We also considered the case for such a regimen from multiple economic perspectives across three high-burden countries. Our findings were robust to parameter uncertainty and remained qualitatively consistent across several sensitivity analyses.

In summary, if ambitious targets for a pan-tuberculosis regimen are met, expected health gains are substantial and potential savings could offset drug prices. Many similar benefits could be achieved with an improved non-pan-tuberculosis regimen if most patients were eligible for the regimen and ineligible patients were readily identifiable. However, a pan-tuberculosis regimen would offer additional advantages, incrementally improving health outcomes and reducing non-drug costs. When paired with appropriate drug resistance surveillance and awareness that some specific clinical situations require non-standard management, such a regimen could be a valuable tool for combatting tuberculosis. Despite recent advances in tuberculosis therapeutics, continuing to pursue the development and evaluation of potentially transformative novel compounds and regimens remains an important priority for the global tuberculosis community.

## Contributors

## Data sharing

All data used in this study are from publicly available sources that are listed in the appendix (pp 3–5, 15–20) and are cited in the references. Model code is available at: github.com/rycktessman/pan-tb-modeling.

## Declaration of interests

TSR reports funding for work outside of this study from WHO. CFM was funded for work outside of this study by the Bill & Melinda Gates Foundation (TB MAC OPP1135288) and Unitaid (20193-3-ASCENT). All other authors declare no competing interests.
