# Supplementary material for: Projected health and economic effects of a pan-tuberculosis treatment regimen: a modelling study
Source: Lancet Glob Health. 2024 Aug 16;12(10):e1629–37. doi: 10.1016/S2214-109X(24)00284-5 (PMC11413512; doi:10.1016/S2214-109X(24)00284-5)
Supplement: Supplementary appendix [file mmc1.pdf]

THE LANCET
Global Health

Supplementary appendix

This appendix formed part of the original submission and has been peer reviewed. We post it as supplied by the authors.

Supplement to: Ryckman TS, McQuaid CF, Cohen T, Menzies NA, Kendall EA. Projected health and economic effects of a pan-tuberculosis treatment regimen: a modelling study. *Lancet Glob Health* 2024; published online Aug 16. https://doi.org/10.1016/S2214-109X(24)00284-5.

Supplementary Appendix to “Projected health and economic effects of a pan-tuberculosis treatment regimen: a modelling study”

TABLE OF CONTENTS

Supplementary Methods 3
Appendix Table 1: Model Parameters 3
Appendix Text 1: Model Description and Additional Details 6
Appendix Figure 1: Assumed trends in drug resistance prevalence by country and scenario 9
Appendix Figure 2: Pretreatment model, standard of care and pan-TB scenarios 10
Appendix Figure 3: Modeled relationship between loss-to-follow-up over time and probability of cure 11
Appendix Figure 4: Estimated serial interval distribution 12
Appendix Text 2: Incidence estimates 13
Appendix Text 3: Costing Approach 14
Appendix Table 2: Unit cost parameters 15
Appendix Table 3: Quantities of treatment inputs and event probabilities in the cost analysis 18
Appendix Table 4: DALY and disability weight parameters 20
Appendix Table 5: CHEERS checklist for economic evaluation studies 21
Appendix Text 4: One-Way and Additional Sensitivity Analyses 23
Appendix Table 6: Optimistic and pessimistic regimen characteristics used in one-way sensitivity analysis 24
Supplementary Appendix References 25
Supplementary Results 30
Appendix Table 7: Health impact of Pan-TB and isolated improvement scenarios 30
Appendix Figure 5: Projected reductions in TB incidence from Pan-TB regimens and other regimen improvements 32
Appendix Table 8: Incremental health impact of Pan-TB Scenario vs. Improved Rifampin-Susceptible Regimen Scenario 33
Appendix Figure 6: Initial durable cures, by drug resistance phenotype, country, and scenario 34
Appendix Figure 7: Costs per person by cost category, country, and scenario 36
Appendix Figure 8: Cost-saving price thresholds of Pan-TB regimens under alternative comparators 37
Appendix Figure 9: Uncertainty in cost-saving price thresholds 39
Appendix Figure 10: DALYs per person by type, country, and scenario 40
Appendix Figure 11: Cost-effective price thresholds for a Pan-TB regimen 41
Appendix Figure 12: Sensitivity of cost-effective prices to willingness-to-pay 42
Appendix Figure 13: Effects of varying oral pan-TB regimen characteristics on initial durable cures ranges 44
Appendix Figure 14: Effects of varying oral pan-TB regimen characteristics on TB deaths 45
Appendix Figure 15: Effects of varying oral pan-TB regimen characteristics on secondary cases 46
Appendix Figure 16: Effects of varying oral pan-TB regimen characteristics on the cost-saving price thresholds 47

Appendix Text 5: Results of additional sensitivity analyses 49
Appendix Figure 13: Proportion of patients cured with standard of care and pan-TB regimens, under additional sensitivity analyses 50
Appendix Figure 14: TB deaths with standard of care and pan-TB regimens, under additional sensitivity analyses 51
Appendix Figure 15: Secondary cases with standard of care and pan-TB regimens, under additional sensitivity analyses 52
Appendix Figure 16: Short-term non-drug costs with standard of care and pan-TB regimens, under additional sensitivity analyses 53
Appendix Table 9: Percent decrease in proportion of patients not initially cured from regimen improvements, under additional sensitivity analyses 54
Appendix Table 10: Percent decline in TB deaths from regimen improvements, under additional sensitivity analyses 55
Appendix Table 11: Percent decline in secondary cases from regimen improvements, under additional sensitivity analyses 56
Appendix Table 12: Cost-saving price thresholds, under additional sensitivity analyses 57

SUPPLEMENTARY METHODS

Appendix Table 1: Model Parameters

Parameter	Country or Regimen	Estimate [95% uncertainty interval]	Distribution used for parameter sampling*	Sources/Notes
Baseline prevalence of rifampin resistance	India	2·5% [2·3-2·7%]	Normal (0·025, 0·0010)	WHO estimates , based on drug resistance surveys & surveillance[1]
	South Africa	4·1% [3·9-4·2%]	Normal (0·041, 0·0008)	
	Philippines	1·5% [1·0-2·1%]	Normal (0·015, 0·0028)	
Baseline prevalence of novel resistance, among new RS-TB	India	0·2% [0·0-1·0%]	Beta (0·6, 299·4)	Timm et al. 2023[2]
	South Africa	0·2% [0·0-1·0%]		
	Philippines	0·2% [0·0-1·0%]		
Baseline prevalence of novel resistance, among new RR-TB	India	0·7% [0·4-1·4%]	Equals prevalence in South Africa, multiplied by Beta (15, 85) and divided by Beta (89, 11)	Equals South Africa estimate scaled by average BDQ usage over 2017-21
	South Africa	3·6% [2·8-4·6%]	Beta (72, 1951)	Based on Ismail et al. 2022[3]
	Philippines	1·3% [0·9-2·0%]	Equals prevalence in South Africa, multiplied by Beta (30, 70) and divided by Beta (89, 11)	Equals South Africa estimate scaled by average BDQ usage over 2017-21
Pre-treatment LTFU if assigned to RS SOC or Pan-TB regimen	India	13% [8-19%]	Normal (mean 0·134, sdev 0·028)	Subbaraman et al. 2016; Naidoo et al. 2017[4,5]
	South Africa			
	Philippines			
Additional pre-treatment LTFU if assigned to a separate care pathway (RR SOC or individualized regimen)	India	16% [7-27%]	Beta (8, 42)	Based on Subbaraman et al. 2016, Cox et al. 2017, and WHO notifications data[1,4,6].
	South Africa			
	Philippines			
% patients receiving rifampin DST, status quo	India	49% [35-63%]	Beta (24·5, 25·5)	WHO drug resistance surveillance data[1]
	South Africa	67% [54-79%]	Beta (33·5, 16·5)	
	Philippines	40% [28-53%]	Beta (22·9, 34·3)	
% patients receiving rifampin DST, improved DST scenario	India	90%	NA – part of scenario assumptions; no uncertainty modeled	Assumed (based on assumed % of pulmonary TB that could feasibly be tested given microbiological positivity).
	South Africa			
	Philippines			
% detected RR patients receiving DST for novel drugs, status quo	India	6% [5-7%] to 60% [46-73%]	Linear increase from 0% (no uncertainty) to Beta (30, 20)	Increases linearly over time, from 0% (with no uncertainty) prior to year 1, to a maximum level in year 10 that is based off current FQ testing among RR TB in each country, from WHO drug resistance surveillance data.[1]
	South Africa	9% [8-10%] to 90% [80-97%]	Linear increase from 0% (no uncertainty) to Beta (45, 5)	
	Philippines	2% [1-3%] to 20% [10-32%]	Linear increase from 0% (no uncertainty) to Beta (10, 40)	
% patients receiving DST for novel drugs, main pan-TB scenarios (#2, #3)	All	0%	NA – no uncertainty modeled around 0%	Assumed based on intended use for a pan-TB regimen
% all patients receiving DST for novel drugs, alternative pan-TB scenario with DST (#4)	India	6% [5-7%] to 60% [46-73%]	Same as % detected RR-TB patients receiving DST for novel drugs in the status quo scenario.	Same as % detected RR-TB patients receiving DST for novel drugs in the status quo scenario.
	South Africa	9% [8-10%] to 90% [80-97%]		
	Philippines	2% [1-3%] to 20% [10-32%]		
Weekly probability of early discontinuation (all regimens except Pan-TB LAI)	India	0·13% [0·08-0.18%]	Beta (26, 19923)	WHO data.[1] Assumed to be constant over time based on Kruk et al. 2008[7]; see also Appendix Figure 3.
	South Africa	0·41% [0·31-0·52%]	Beta (58, 14168)	
	Philippines	0·14% [0·08-0·20%]	Beta (20, 14824)	
Relative risk of cure if rifampin resistant (when assigned to rifamycin-containing regimen), versus rifampin susceptible	Rifamycin-containing regimen	35% [22-50%]	Beta (17·5, 32·5)	Cox et al. 2006; He et al. 2010[8,9]
Relative risk of cure if novel-resistant (if assigned to novel drug-containing regimen), versus novel-susceptible	RR SOC and Pan-TB regimens	77% [50-97%]	Beta (9, 3) divided by Beta (179, 6)	Timm et al. 2023[2]

Efficacy	RS-TB SOC	95% [93-97%]	See Appendix Text 1, *Model assumptions: isoniazid and fluoroquinolone resistance under the standard of care* section	Gegia et al. 2016[10]
	RR-TB SOC	89% [83-94%]	Beta (96, 11)	Conradie et al. 2020, Conradie et al. 2022, Nyang'wa et al. 2022[11–13]
	Pan-TB TRP	95% [93-97%]	Same as RS SOC, not modeled independently	
	Individualized	75% [67-83%]	Beta (75, 25)	Based on outcomes of MDR-TB patients pre-BPaL/BPaLM[14]
Duration	RS-TB SOC	24 weeks	NA – no uncertainty in duration was modeled	WHO guidelines[15]
	RR-TB SOC	26 weeks		WHO guidelines[16]
	Pan-TB oral	14 weeks		WHO target regimen profile (minimal target)[17]
	Pan-TB LAI	Not Applicable		1-time injectable
	Individualized	18 months		Based on RR regimens pre-BPaL/BPaLM
% patients w/ < 70% adherence	RS-TB SOC	38% [28-48%]	Multinomial (100, 0·379)	Median of control groups in 3 adherence-improving intervention studies[18–20]
	RR-TB SOC	40% [31-49%]	RS-TB proportion plus 2%	Worst of control groups in 3 adherence-improved intervention studies[18–20]
	Pan-TB oral	14% [12-16%]	Multinomial (100, 0·14)	Best of control groups in 3 adherence-improving 3 intervention studies[18–20], based on minimal TRP target of better tolerability than the standard of care.[17]
	Pan-TB LAI	0%	NA – no uncertainty modeled around 0%	Assumed (no uncertainty modeled).
	Individualized	38% [28-48%]	Same as RR-TB	Assumed same as BPaL/BPaLM.
% patients w/ 70-90% adherence	RS-TB SOC	31% [18-46%]	Multinomial (100, 0·312)	Median of control groups in 3 adherence-improving intervention studies[18–20]
	RR-TB SOC	34% [21-50%]	RS-TB proportion plus 4%	Worse of control groups in 3 adherence-improved intervention studies[18–20]
	Pan-TB oral	34% [31-38%]	Multinomial (100, 0·35)	Best of control groups in 3 adherence-improving 3 intervention studies[18–20], based on minimal TRP target of better tolerability than the standard of care.[17]
	Pan-TB LAI	0%	NA – no uncertainty modeled around 0%	Assumed (no uncertainty modeled).
	Individualized	31% [18-46%]	Same as RR-TB	Assumed same as HRZE.
% patients w/ ≥ 90% adherence	RS-TB SOC	31% [22-40%]	Multinomial (100, 0·309)	Median of control groups in 3 adherence-improving intervention studies[18–20]
	RR-TB SOC	26% [16-34%]	RS-TB proportion minus 6%	Worst of control groups in 3 adherence-improved intervention studies[18–20]
	Pan-TB oral	51% [47-55%]	Multinomial (100, 0·51)	Best of intervention groups in 3 adherence-improving 3 intervention studies[18–20], based on minimal TRP target of better tolerability than the standard of care.[17]
	Pan-TB LAI	100%	NA – no uncertainty modeled around 0%	Assumed (no uncertainty modeled).
	Individualized	31% [22-40%]	Same as RR-TB	Assumed same as HRZE.
Forgiveness (nonadherence threshold above which probability of cure < efficacy)	RS-TB SOC	10%	NA – no uncertainty was modeled in the forgiveness thresholds, but uncertainty was included in the relative probability of cure above vs. below the forgiveness threshold (next row).	Imperial et al.[21]
	RR-TB SOC	15%		Assumed slightly better than HRZE, given better pharmacokinetic properties[22]
	Pan-TB oral	15%		Value from WHO minimal TRP.[17]
	Pan-TB LAI	Not Applicable		No nonadherence with a long-acting injectable.
	Individualized	10%		Assumed same as HRZE.
Relative probability of cure if missed doses exceed the forgiveness threshold	All	82% [63-94%]	Simulated using the confidence intervals from Imperial et al. [21], assuming Wald distributions.	Imperial et al.[21]
Case fatality ratio, incident TB	India	16% [11-24%]	Normal (451,000, 16,937) divided by Normal (2,960,000, 522,959)	WHO, using 2019 estimates to exclude temporary COVID-related effects[1]. To sample from this parameter, we sampled from both incidence and mortality

	South Africa	17% [8-28%]	Normal (58,000, 13,520) divided by Normal (357,000, 60,459)	distributions, and then divided the mortality samples by the incidence samples.
	Philippines	5% [3-10%]	Normal (30,000, 765) divided by Normal (612,000, 156,378)	
Proportion of unsuccessful treatments resulting in death	India	48% [36-60%]	Beta (31·5, 34·1)	WHO treatment outcomes data, using 2019 data to exclude temporary COVID-related effects[1]
	South Africa	40% [30-50%]	Beta (36·5, 54·7)	
	Philippines	34% [26-43%]	Beta (40·2, 78·1)	
Case detection ratio	India	73% [53-90%]	Beta (11·8, 4·4)	WHO, using 2019 estimates to exclude temporary COVID-related effects[1]
	South Africa	59% [43-84%]	Beta (12·5, 8·7)	
	Philippines	67% [43-90%]	Beta (7·7, 3·8)	
Discounted life expectancy of a person w/ TB (years)	India	24·1 [23·5-25·8]	Gamma (772, 0·031)	Overall life expectancy was calculated by combining country-specific estimates of life expectancy by age from WHO life tables with WHO Global TB Report estimates of the country-specific distribution of incident TB by age.[1,23] Discounted life expectancy was then estimated by discounting overall life expectancy by 3% annually.
	South Africa	22·1 [21·4-23·5]	Gamma (957, 0·023)	
	Philippines	23·2 [21·8-26·3]	Gamma (215, 0·108)	
Secondary cases per relapse	All	1	NA – no uncertainty modeled	Assumed based on evidence on the TB reproductive number[24] and an assumption of equal average transmission after relapse as before an initial TB diagnosis.
Relative probability of cure, retreatments	All	86% [50-100%]	Beta (4·40, 0·72)	Ratio of treatment success for new vs. previously-treated patients from WHO outcomes data[1], averaged across all countries that report the data for both patient groups
Willingness to pay thresholds	India	$430	NA – uncertainty incorporated separately (via cost-effectiveness acceptability analysis).	Ochalek et al. 2018[25], updated with 2021 gross national income per capita for each country.
	South Africa	$3400		
	Philippines	$1060		

"RS" = rifampin-susceptible; "RR" = rifampin-resistant; "SOC" = standard of care; "LAI" = long-acting injectable regimen; "DST" = drug-susceptibility testing; "HRZE" = 6 months of isoniazid, rifampin, pyrazinamide, and ethambutol (the standard of care regimen for RS-TB); "BPaLM" = 6 months of bedaquiline, pretomanid, linezolid, and moxifloxacin (the standard of care regimen for RR-TB). "TRP" = target regimen profile.

Both point estimates/means and uncertainty intervals have been estimated from the sources indicated in the "Sources/Notes" column, unless otherwise noted.

*For the uncertainty distributions (column 4), the normal distribution is displayed with mean and standard deviation in parentheses, the beta distribution is displayed with alpha and beta in parentheses, where the mean of a beta distribution is equal to alpha divided by the sum of alpha and beta, the multinomial distribution is displayed with size and probability parameters in parentheses, and the gamma distribution is displayed with shape and scale parameters in parentheses.

Appendix Text 1: Model Description and Additional Details

Study setting

Our modeling analysis was conducted for three high-TB-burden countries: India, South Africa, and the Philippines. Country-specific model inputs included epidemiological parameters (such as TB case fatality and case detection ratio), treatment details (such as monitoring and treatment visit schedules, as well as loss-to-follow-up under the standards of care), and unit cost parameters, each of which are described in more detail in subsequent sections of this appendix. The three countries were chosen because they represent variation in geographics regions, baseline levels of resistance to rifampin and novel drugs, and income (and therefore unit costs and willingness-to-pay).

Model overview

Our TB patient cohort model included 4 components: population-level resistance trends; a pretreatment phase (including regimen assignment and initiation); an on-treatment phase (including adherence, discontinuation, and adverse events); and modeling of subsequent health outcomes (including retreatments, secondary cases, and mortality). The third and fourth components of the model (on-treatment model and modeling of subsequent health outcomes) are fully described in the main text. Additional details are provided below for the first two components (modeling population-level drug resistance and the pretreatment phase).

Model assumptions: population-level drug resistance

Time trends in the proportion of TB with resistance to rifampin and to novel drugs (assumed to be included in both the pan-TB regimen and the rifampin-resistant TB standard of care/RR-TB SOC) were modeled in a simplified manner. At baseline (model year 1, roughly corresponding to 2022), the prevalence of rifampin resistance was based on country-specific surveillance data reported to the WHO,[1] while the prevalence of resistance to novel drugs, stratified by rifampin-resistance status, was based on limited evidence on bedaquiline resistance and adjusted for bedaquiline usage in each country.[1–3]

Estimates of the impact of novel drug resistance on probability of cure (Appendix Table 1) were also based on bedaquiline resistance data; since resistance was dichotomized, our estimates largely capture more common mutations (i.e., Rv0678) that cause lower-level resistance.[2,3]

Each subsequent year, the fraction of TB that was rifampin- or novel-drug-resistant increased linearly at a rate proportional to the modeled percent of treated patients in the previous year that received the corresponding drug (Appendix Figure 1). The increase was capped at one percentage point increase per decade, such that prevalence of a given drug resistance phenotype would increase from baseline levels by one percentage point over ten years if all treated patients were assigned to regimens containing that drug. The assumption of a maximum one percentage point increase in resistance over 10 years was based on observed global prevalence of rifampin-resistance over time (around 3% globally [1]), since the scaleup of rifampin-based regimens globally in the 1990s. The same one percentage point increase per decade was then also used to model trends in resistance to novel drugs (e.g., the pan-TB regimen), based on the minimal target in the WHO target regimen profiles for potential to develop resistance ("no worse than with the SOC").[17] Trends in novel drug resistances were varied in sensitivity analysis.

Model assumptions: pretreatment model

The pretreatment model captured drug susceptibility testing, regimen assignment and initiation, and pre-treatment losses to follow-up (Appendix Figure 2). Drug susceptibility testing practices varied by scenario. Under the standard of care scenario, coverage of rifampin-resistance testing was assumed to remain constant at current country-specific levels, and testing for novel drug resistance among those with detected rifampin resistance increased linearly: from 0% prior to year 1, to current country-specific levels of fluoroquinolone-resistance testing for rifampin-resistant patients by year 10.[1] Fluoroquinolone testing served as a proxy for testing levels that could realistically be achieved for novel drugs. We assumed no novel resistance testing among those without detected rifampin-resistance; estimated prevalence in this population remained very low over the model horizon (Appendix Figure 1).

Regimen assignment also varied by scenario and depended on drug susceptibility testing results. Under the standard of care scenario, those without detected rifampin resistance (those with RS-TB, or with RR-TB that did not receive susceptibility testing) were assigned to the standard of care for treating RS-TB ("RS-TB SOC"; 6HRZE, a six-month regimen that for most patients includes isoniazid, rifampin, pyrazinamide, and ethambutol); those with detected rifampin resistance were assigned to the RR-TB SOC (BPaL[M], a 6 month regimen of bedaquiline, pretomanid, linezolid, and for most patients a fluoroquinolone, which we costed as moxifloxacin), with the exception of those with detected resistance to both rifampin and novel drug(s), who were assigned to individualized regimens that would be based on their confirmed or anticipated drug susceptibility profiles. Pre-treatment losses to follow-up were modeled under all scenarios and regimen assignments, but were assumed to be greater for those assigned to rifampin-resistant or individualized regimens, due to delays incurred in initiating these separate treatment pathways (4,5,16,17). Isoniazid and fluoroquinolone resistance modified the average outcomes and costs of RS- and RR-TB regimens, respectively, as described below.

Under the pan-TB scenario, no drug susceptibility testing was conducted and all patients were assigned to the pan-TB regimen, with pre-treatment losses to follow-up comparable to those among patients assigned to the rifampin-susceptible regimen in the standard of care scenario.

All patients who were modeled as initiating treatment were then included in the on-treatment model (described more in the main text).

Model assumptions: isoniazid and fluoroquinolone resistance under the standard of care

Although we only explicitly modeled resistance to rifampin and to novel drugs, our model also considered isoniazid resistance among rifampin-susceptible patients and fluoroquinolone resistance among rifampin-resistant patients. However, these resistance phenotypes were not modeled explicitly; instead, their effects on treatment outcomes and costs were factored into parameter estimates of the average efficacy and treatment costs under the standard of care (Appendix Table 1).

Starting from estimates of each country's prevalence of isoniazid monoresistance and frequency of isoniazid susceptibility testing among patients with RS-TB, and assuming that patients who were determined to have isoniazid monoresistance would receive a regimen containing levofloxacin, rifampin, pyrazinamide, and ethambutol (with higher costs and the same efficacy as HRZE against fully susceptible TB), we calculated the cure probability with the RS-TB standard of care regimen as a weighted average of treatment outcomes across the fully-susceptible TB treated with HRZE, the isoniazid monoresistant TB treated with HRZE [10], and the isoniazid monoresistant TB treated with the alternative, fluoroquinolone-containing regimen. We modeled HRZE as being 96% [94-97%] efficacious against isoniazid-susceptible (and rifampin-susceptible) TB (sampling from a beta distribution with alpha=576 and beta=24) and 84% [79-89%] efficacious against isoniazid-resistant (rifampin-susceptible) TB (sampling from a beta distribution with alpha=168 and beta=32).[10] We used the same samples drawn for HRZE efficacy against isoniazid-susceptible TB to parameterize the efficacy of the levofloxacin/rifampin/pyrazinamide/ethambutol regimen.

The downstream effects of this efficacy on costs, as well as the direct costs of isoniazid susceptibility testing and levofloxacin for those who received them, were also factored into our weighted average estimates of costs [1,26–28].

Similarly, costs of rifampin-resistant TB treatment under the standard of care were a weighted average of costs for BPaL with and without fluoroquinolone susceptibility testing and moxifloxacin as part of the regimen (no moxifloxacin for patients with detected resistance) [27–29].

Model input parameters

Model parameters were sourced from the published literature. All parameters are described in appendix tables 1-4.

Time horizon

The analysis considered a ten-year policy and analytical horizon.

Sensitivity analysis

We conducted a probabilistic sensitivity analysis that propagated uncertainty in all model parameters by sampling 10,000 parameter sets from parameter uncertainty distributions (see appendix tables 1-4) and calculating means and 95% uncertainty intervals (2·5th and 97·5th percentiles) across the resulting 10,000 sets of modeled output. We also conducted one-way sensitivity analysis on the 6 characteristics of the Pan-TB regimen (efficacy, duration, ease of adherence, forgiveness, safety, and barrier to resistance). Finally, we conducted select scenario analyses, which are described more in appendix text 4.

Model calibration

Since we only modeled cohorts of patients from the point of diagnosis (i.e., this was not a population-level transmission model) and model parameters could be sourced from the published literature, no model calibration was conducted.

Appendix Figure 1: Assumed trends in drug resistance prevalence by country and scenario

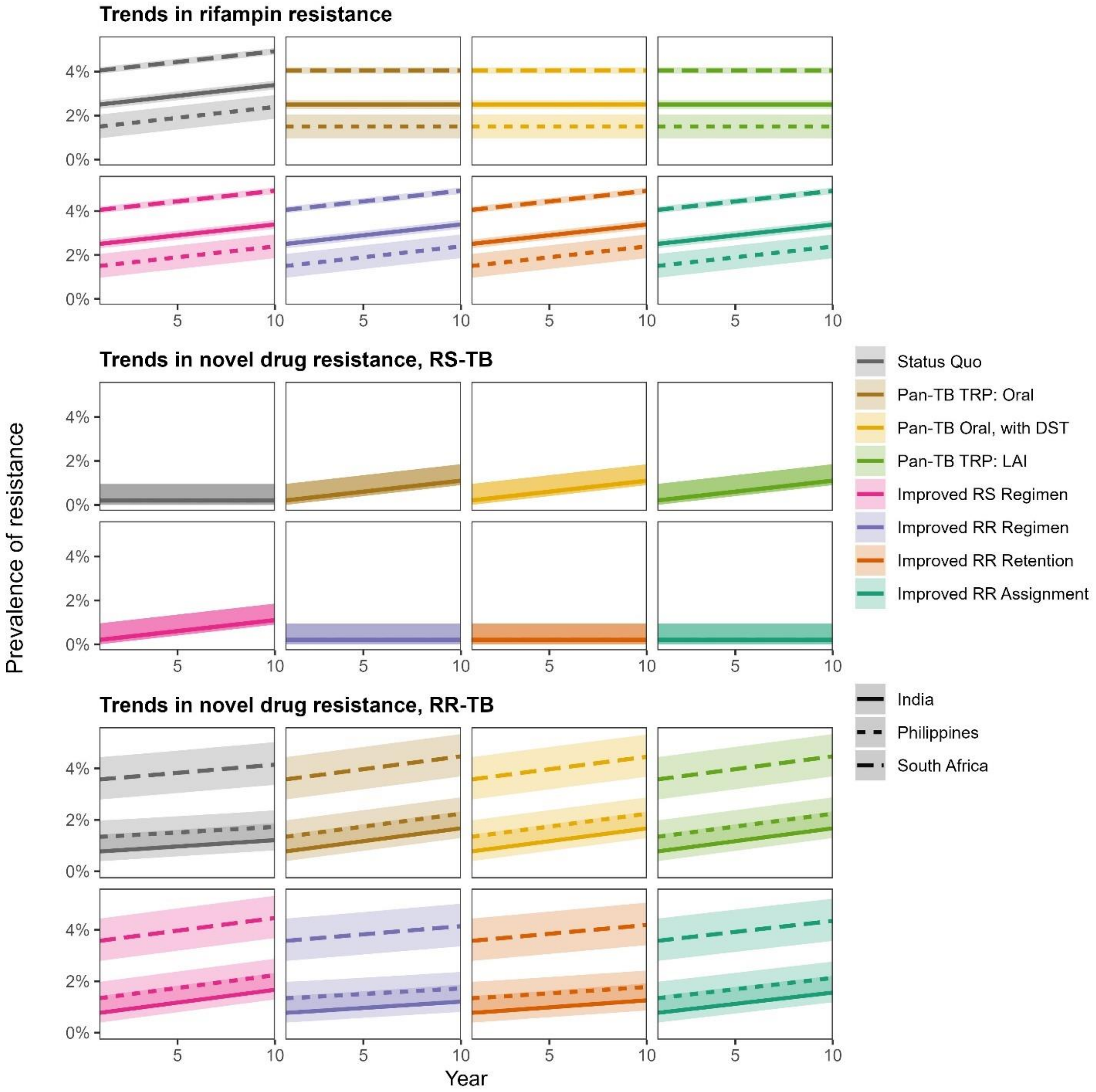

Figure shows trends in the proportion of all TB that is rifampin resistant (top panel) the proportion of rifampin-susceptible (RS) TB that is resistant to novel drugs (middle panel), and the proportion of rifampin-resistant (RR) TB that is resistant to novel drugs (bottom panel) over time, across three modeled countries (line styles) and eight modeled scenarios (colors). Darker lines indicate mean estimates, while lighter shaded area indicate 95% uncertainty intervals. "RS" = rifampin-susceptible; "RR" = rifampin-resistant. Baseline levels of and trends in resistance to novel drugs among patients with RS-TB were assumed to be the same across all countries; modeling assumptions are described in more detail in Appendix Text 1.

Appendix Figure 2: Pretreatment model, standard of care and pan-TB scenarios

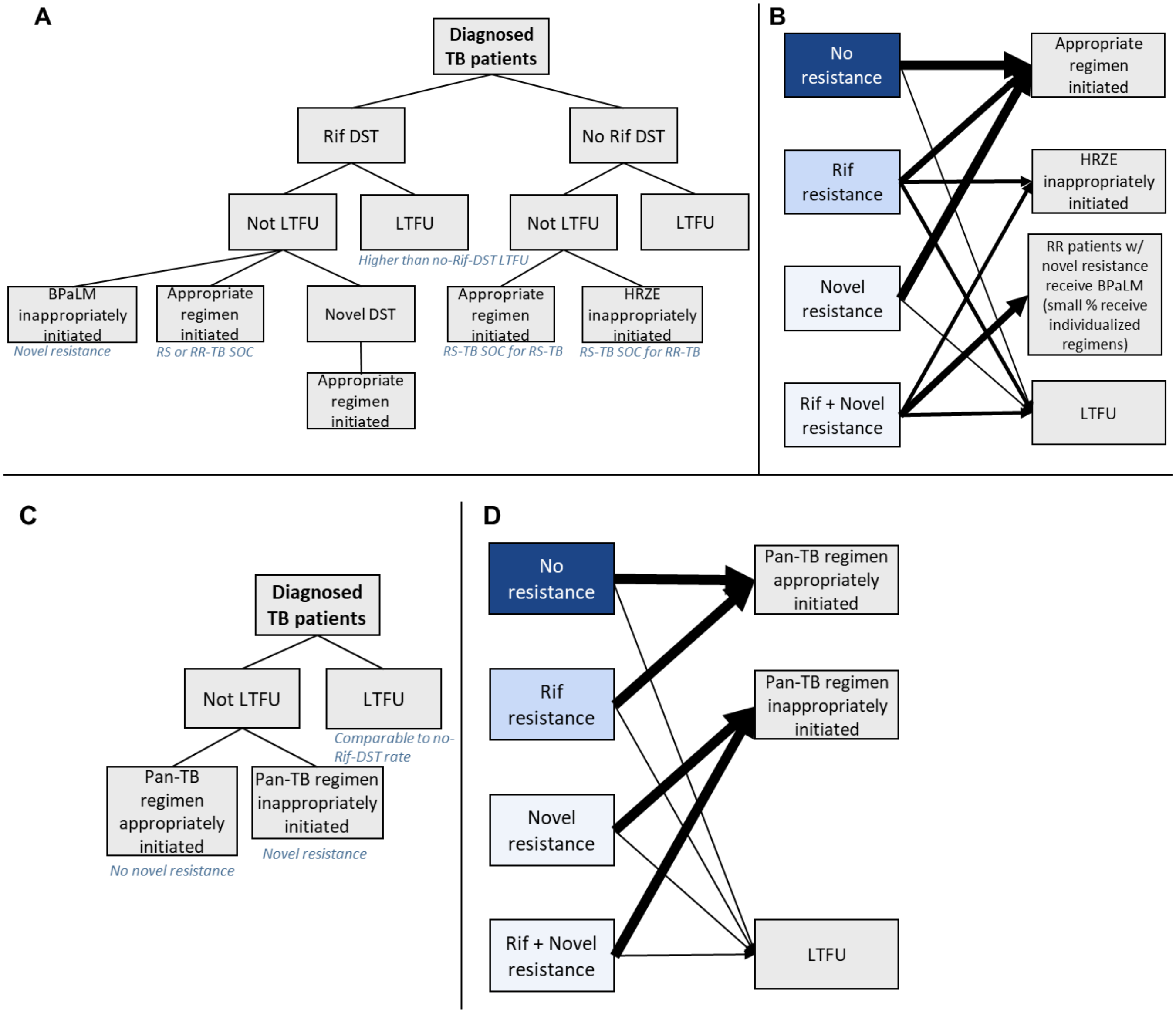

Figure shows drug susceptibility testing, pre-treatment losses to follow-up, and regimen assignment under the standard of care scenario (panels A and B) and the pan-TB scenario (panels C and D). In panels B and D, the drug resistance phenotypes are shaded according to the percent of the patient population with each phenotype (darker shading = higher prevalence) and the arrow widths correspond to the proportion of each phenotype assigned to each regimen (thicker arrows = more patients assigned). "RS" = rifampin-susceptible; "RR" = rifampin-resistant. "DST" = drug-susceptibility testing; "LTFU" = lost-to-follow-up; "HRZE" = 6 months of isoniazid, rifampin, pyrazinamide, and ethambutol (the standard of care for treating rifampin-susceptible TB); "BPaLM" = 6 months of bedaquiline, pretomanid, linezolid, and moxifloxacin (the standard of care for treating rifampin-resistant TB).

Appendix Figure 3: Modeled relationship between loss-to-follow-up over time and probability of cure

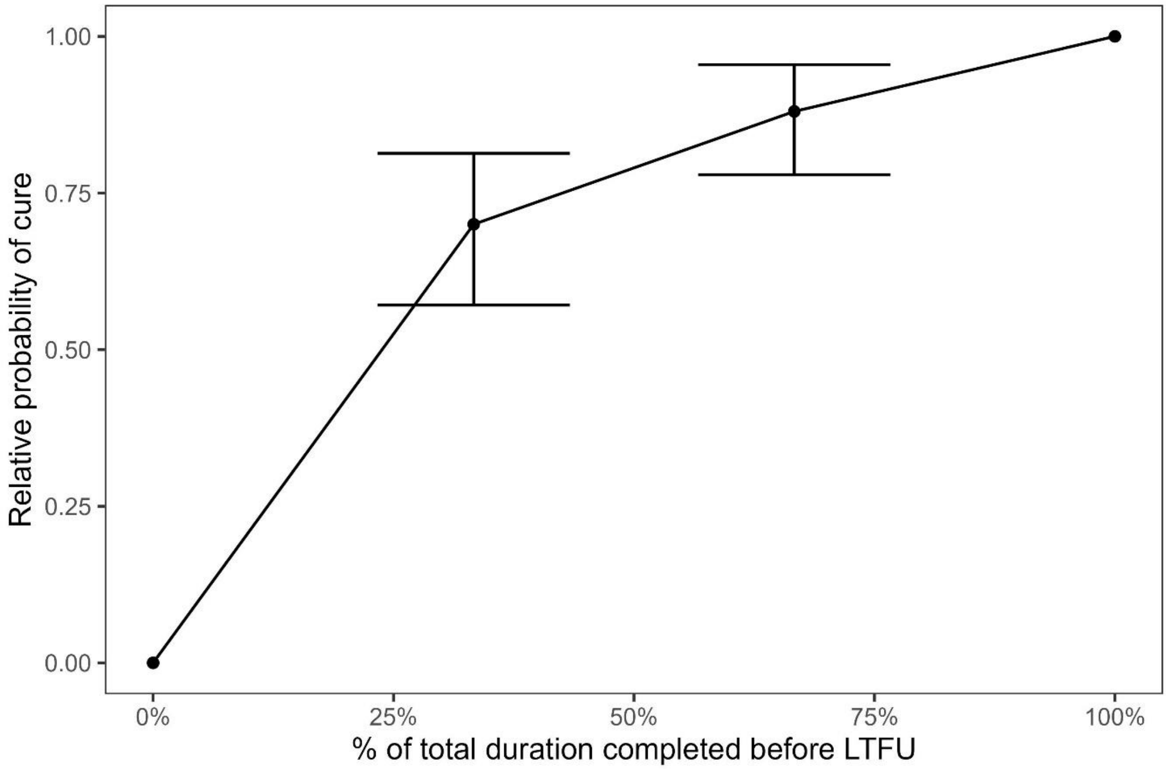

Figure shows the relative probability of cure (relative to probability of cure with 100% completion) depending on the percentage of treatment completed before a patient discontinues or is lost-to-follow-up (LTFU). This figure does not include reductions in the probability of cure due to poor adherence while still on treatment, which is modeled separately. This relationship was estimated based on evidence from historical trials of shorter HRZE regimens [30,31] and was applied to all regimens. Error bars indicate 95% uncertainty intervals in the relative probability of cure at one-third and two-thirds of regimen duration completed.

Appendix Figure 4: Estimated serial interval distribution

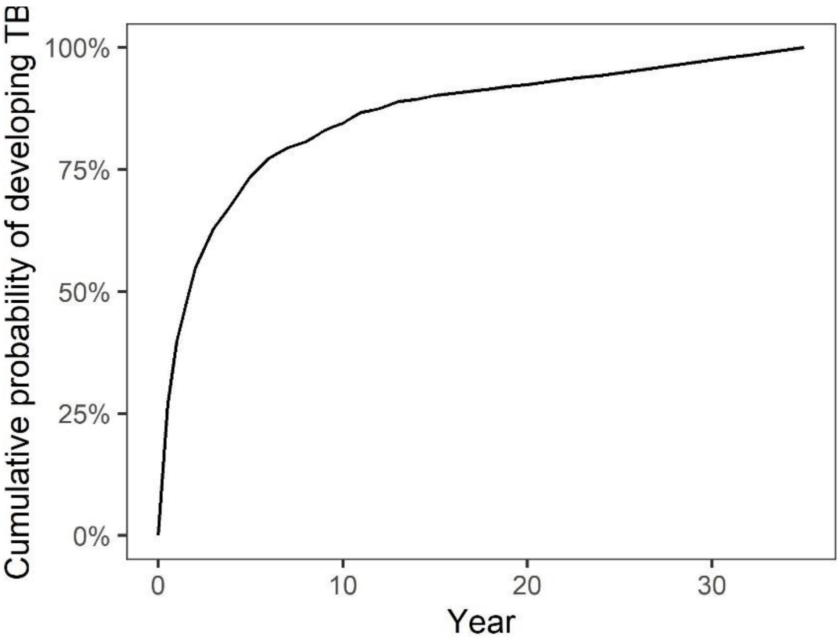

Figure shows the cumulative probability of a secondary case having developed TB disease by year since the index case developed TB disease, conditional on the secondary case eventually developing TB disease. Estimates are based on data reported in [32,33]. In the model, we added six months to this distribution to account for the average delay between when treatment would have been initiated and when a patient would become infectious again.

Appendix Text 2: Incidence estimates

To estimate incidence reductions from improved regimen scenarios, we estimated secondary cases resulting from each year's patient cohort (i.e., from transmission that occurred after TB diagnosis) under the standard of care. We used these estimates to estimate the secondary cases that could be averted through improved treatment outcomes, and adjusted the sizes of subsequent cohorts in improved regimen scenarios accordingly. For the latter calculation, we assumed that under the SOC the total number of TB patients in each country would remain at 2019 levels; for all other scenarios, we estimated year-to-year reductions in cohort sizes, as described below.

We started by assuming annual cohorts of 100 incident TB cases under the standard of care. For each annual cohort, based on the serial interval distribution (Appendix Figure 4), we estimated the proportion of the cohort that had been infected prior to year 1 of the model; this "pre-model" cohort size was 100 people in year 1, 60 people in year 2 (because 60% of the serial interval distribution was greater than 1 year), 45 people in year 3, etc. The pre-model cohort sizes remained fixed across scenarios, because reductions in transmission were assumed to only occur after regimen improvements were introduced in year 1.

We then used equation 1 to estimate, for each regimen scenario *q,* the number of secondary cases accruing from the year 1 cohort in each subsequent year $t \geq 2$. In this equation, *cohort* is the size of the cohort in year 1 (100), *serial* is the proportion of secondary cases arising from the year 1 cohort that accrue in each subsequent year (i.e., between year *t-1* and year *t*), *cdr* is the case detection ratio, *p_fail* is the proportion of all patients that are not microbiologically cured in year 1 under the specified scenario, and *SOC* refers to the standard of care scenario. $cases_{t,q,s}$ refers to the number of cases generated in year *t,* under scenario *q,* by transmission arising from the year *s* cohort after they had been diagnosed with TB.

[1] $cases_{t,q,1} = cohort_{q,1} * \int_{t-1}^{t} serial * (1 + cdr * p\_fail_{q,1})/(1 + cdr * p\_fail_{SOC,1})$

We used this result to recalculate the size of the year 2 cohort in each scenario, as the size of the pre-model year 2 cohort (60) plus the secondary cases occurring in year 2 from the year 1 cohort (40 under the status quo, < 40 under other scenarios):

[2] $cohort_{q,2} = cases_{2,q,1} + pre\_cohort_2$

We then repeated similar steps iteratively through year 10 (the end of the modeled time horizon) to estimate the cohort sizes in all years under each scenario *q*. For each year *s* from *s*=2 to *s*=9, we estimated the number of secondary cases accruing in all subsequent years *t* (where $s < t \leq 10$) from the cohort diagnosed in year *s:*

[3] $cases_{t,q,s} = cohort_{q,s} * \int_{t-1}^{t} serial * (1 + cdr * p\_fail_{q,s})/(1 + cdr * p\_fail_{SOC,s})$

We added these estimated secondary cases to each year's pre-model cohort to estimate the cohort size for each subsequent year *s+1*:

[4] $cohort_{q,s+1} = \sum_{i=1}^{s} cases_{s+1,q,i} + pre\_cohort_{s+1}$

Finally, we calculated the percent reduction in cohort sizes each year under each scenario, and applied these percent reductions to current estimated incidence in each country, in order to estimate trends in incidence over time under each scenario.

Appendix Text 3: Costing Approach

Costing perspective

We adopted both health system (considering medical costs only) and societal (considering both medical costs and non-medical costs borne by people with TB) perspectives.

Other cost details

We estimated costs using an ingredients-based approach that multiplied country-specific unit costs (which were fixed across scenarios; Appendix Table 2) by the country-specific quantities of inputs required under each scenario (Appendix Table 3). Prior to treatment initiation, patients accrued diagnostic, drug susceptibility testing, and patient care-seeking costs accrued. In addition, costs of drugs, routine outpatient visits and inpatient care for severe cases, laboratory tests to monitor adverse events and treatment outcomes, adverse event management, patient support vouchers, and patient-borne out-of-pocket and time costs accrued each week a patient remained in care. After treatment, we included retreatment costs for patients from the initial cohort who were retreated, and the costs of treating secondary cases for those secondary cases who were detected. For secondary cases, costs were equal to the average cost of treating a new patient in a given year, stratified by drug resistance. For retreatments, costs were assumed to be 10% higher than average initial treatment costs (again stratified by drug resistance), reflecting the potential for more extensive drug susceptibility testing, assignment to a more expensive regimen, or increased monitoring of previously treated patients.

Unit costs were all converted to 2021 US Dollars (USD). This adjustment was accomplished by converting prices reported in USD for earlier years to local currency units, inflating to 2021 local currency using medical currency consumer price indices[34–36] and finally converting to 2021 USD using 2021 exchange rates.[37]

In the cost-effectiveness analysis, both costs and disability-adjusted life years (DALYs) were discounted at 3% annually, in keeping with standard cost-effectiveness guidelines.[38]

Appendix Table 2: Unit cost parameters

Cost Component	India			South Africa			Philippines		
	Estimate [95% uncertainty interval]	Distribution used for parameter sampling*	Source	Estimate [95% uncertainty interval]	Distribution used for parameter sampling*	Source	Estimate [95% uncertainty interval]	Distribution used for parameter sampling*	Source
**Outpatient Treatment and Monitoring Visits**									
Outpatient visit	$2·12 [$1·17-3·07]	Gamma (19·1, 0·11)	[39]	$14·83 [$9·85-20·93]	Gamma (28·5, 0·5)	[40]	$3·47 [$2·49-4·58]	Gamma (40·0, 0·09)	[41]
**Laboratory Tests/Screening**									
Sputum smear microscopy	$2·37 [$1·58-3·31]	Gamma (28·2, 0·08)	[39 42]	$9·02 [$6·78-11·61]	Gamma (53·1, 0·17)	[43 42]	$5·79 [$3·43-8·72]	Gamma (18·9, 0·31)	[41 42]
Sputum Culture	$10·26 [$6·50-14·83]	Gamma (23·3, 0·44)		$20·37 [$7·74-41·00]	Gamma (5·2, 3·9)		$27·78 [$22·65-33·47]	Gamma (100·2, 0·28)	
Xpert MTB/RIF and Xpert Ultra	$23·18 [$10·98-39·51]	Gamma (11·9, 2·12)		$23·42 [$17·64-29·71]	Gamma (67·6, 0·4)		$25·74 [$23·22-28·37]	Gamma (454·6, 0·06)	
Xpert XDR	$35·18 [22·98-51·51]	Xpert cost plus $12		$35·42 [$29·64-41·71]	Xpert cost plus $12		$37·74 [$35·22-40·37]	Xpert cost plus $12	
Novel drug susceptibility testing	Same as Xpert XDR			Same as Xpert XDR			Same as Xpert XDR		
Chest Xray	$3·53 [$2·17-5·25]	Gamma (20·1, 0·18)		$15·79 [$4·13-35·54]	Gamma (3·8, 4·1)		$4·84 [$3·50-6·37]	Gamma (44·3, 0·11)	
Liver function testing	$3·61 [$2·86-4·46]	Gamma (79·2, 0·05)		$9·67 [$5·45-14·95]	Gamma (15·4, 0·63)	[44]	$4·46 [$3·23-5·86]	Gamma (45·2, 0·10)	
Full blood count	$1·16 [$0·73-1·70]	Gamma (22·0, 0·05)		$4·45 [$2·48-6·91]	Gamma (15·4, 0·29)		$3·80 [$2·34-5·66]	Gamma (10·2, 0·19)	
ECG	$1·51 [$0·64-2·76]	Gamma (7·7, 0·20)		$14·61 [$10·70-19·21]	Gamma (44·9, 0·33)	[45]	$5·62 [$2·71-9·50]	Gamma (10·2, 0·55)	
Neuropathy screening	$1·06 [$0·59-1·75]	Outpatient visit cost divided by 2	Clinician time; assumed to be half the cost of an outpatient visit	$7·42 [$4·94-10·41]	Outpatient visit cost divided by 2	Clinician time; assumed to be half the cost of an outpatient visit	$1·74 [$1·24-2·29]	Outpatient visit cost divided by 2	Clinician time; assumed to be half the cost of an outpatient visit
**Adverse Events**									
Liver dysfunction	$154 [$87-240]	Gamma (15·4, 10·0)	[45]	$728 [$408-1139]	Gamma (15·4, 47·4)	[45,46]	$241 [$136-380]	Gamma (15·4, 15·7)	[45]
Pancreatitis	$134 [$78-210]	Gamma (15·4, 8·7)		$472 [$267-734]	Gamma (15·4, 30·7)		$209 [$117-323]	Gamma (15·4, 13·6)	
Anemia	$65 [$36-101]	Gamma (15·4, 4·2)		$97 [$55-151]	Gamma (15·4, 6·3)		$102 [$57-158]	Gamma (15·4, 6·6)	
Neutropenia	$8 [$4-12]	Gamma (15·4, 0·5)		$102 [$58-158]	Gamma (15·4, 6·6)		$13 [$7-19]	Gamma (15·4, 0·8)	
QTcF prolongation	$138 [$77-214]	Gamma (15·4, 9·0)		$517 [$292-810]	Gamma (15·4, 33·6)		$215 [$120-335]	Gamma (15·4, 14·0)	
Renal disfunction	$146 [$82-227]	Gamma (15·4, 9·5)		$619 [$344-961]	Gamma (15·4, 40·3)		$227 [$128-353]	Gamma (15·4, 14·8)	

Vision	$10 [$6-16]	Gamma (15·4, 0·7)	South Africa cost scaled by relative GNI p.c.	$30 [$17-47]	Gamma (15·4, 2·0)		$17 [$10-26]	Gamma (15·4, 1·1)	South Africa cost scaled by relative GNI p.c.
Arthralgia	$5 [$3-7]	Gamma (15·4, 0·3)		$14 [$8-22]	Gamma (15·4, 0·9)		$8 [$4-12]	Gamma (15·4, 0·5)	
Peripheral neuropathy	$0	No uncertainty modeled in parameters equaling 0	Only affects DALYs & monitoring costs	$0	No uncertainty modeled in parameters equaling 0	Only affects DALYs & monitoring costs	$0	No uncertainty modeled in parameters equaling 0	Only affects DALYs & monitoring
**Treatment Support**									
Treatment vouchers (for expenses) per month, RS-TB	$7 [$4-10]	Gamma (15·4, 0·4)	[47]	$0	No uncertainty modeled in parameters equaling 0	[48]	$0	No uncertainty modeled in parameters equaling 0	Estimate from [49,50]
Treatment vouchers (for expenses) per month, RR-TB	$7 [$4-10]	Gamma (15·4, 0·4)		$134 [$76-210]	Gamma (15·4, 8·7)		$30 [$17-47]	Gamma (15·4, 2·0)	
**Hospitalization (conditional on being hospitalized)**									
Cost per episode, RS-TB	$76 [$51-106]	Gamma (28·9, 2·6)	[39]	$266 [$175-375]	Gamma (26·7, 10·0)	[40]	$134 [$101-172]	Gamma (56·6, 2·4)	[51]
Cost per episode, RR-TB	$400 [$192-689]	Gamma (9·7, 41·0)	[39]	$1819 [$1190-2574]	Gamma (26·7, 68·1)	[40]	$542 [$411-690]	Gamma (56·6, 9·6)	[51]
**Drugs (cost per full course)**									
RS SOC#	$46·5 [$46·3-46·7]	#	[52]	$46·5 [$46·3-46·8]	#	[52]	$47·1 [$47·0-47·6]	#	[52]
RR SOC#	$430·5 [$429·8-431·0]	#		$429·0 [$426·8-430·7]	#		$432·0 [$431·7-432·1]	#	
Individualized Regimen	$2526	No uncertainty modeled		$2526	No uncertainty modeled		$2526	No uncertainty modeled	
Wastage	8% [5-12%]	Beta (16, 184)	[39]	5% [2-8%]	Beta (10, 190)	Assumed	5% [2-8%]	Beta (10, 190)	[41]
**Patient-Borne Out-of-Pocket Costs**									
Pre-diagnosis (RS)^	$16 [$0-61]	Gamma (1·0, 16·3)	[53]	$22 [$13-35]	Gamma (15·4, 1·5)	[54]	$3 [$1-8]	Gamma (3·3, 1·0)	[50,51]
Pre-diagnosis (RR)^	$32 [$0-122]	Doubling of RS pre-diagnostic costs		$44 [$26-70]	Doubling of RS pre-diagnostic costs		$7 [$0-27]	Doubling of RS pre-diagnostic costs	
Treatment (RS)^ per month	$5 [$1-10]*	Gamma (3·8, 1·2)	[53,55–57]	$24 [$2-76]	Gamma (1·5, 16·3)	[54,58,59]	$41 [$14-81]	Gamma (5·4, 45·6)	
Treatment (RR)^ per month	$25 [$1-96]	Gamma (1·0, 26·3)	[60]	$53 [$4-167]^	Gamma (1·5, 36·9)	[61]	$155 [$99-225]^	Gamma (23·2, 80·4)	
**Patient-Borne Indirect Costs (i.e., productivity/lost wages)**									
Pre-diagnosis (RS)	$83 [23-182]	Gamma (4·2, 20·0)	[53,57]	$48 [$27-75]	Gamma (15·4, 3·1)	[54]	$45 [$26-71]	Gamma (15·4, 2·9)	

Pre-diagnosis (RR)	$166 [46-364]	Doubling of RS pre-diagnosis costs		$96 [$54-150]	Doubling of RS pre-diagnosis costs		$82 [$46-127]	Doubling of RS pre-diagnosis costs	
Treatment intensive phase (RS)	$64 [$17-141] total	Gamma (4·0, 16·0)	53,55–57,60	$82 [$46-128] total	Gamma (15·4, 5·3)	54	$3 [$2-4] per outpatient visit + $252 [$142-394] per episode of hospitalization	Gamma (15·4, 0·17) + Gamma (15·4, 16·4)	
Treatment continuation phase (RS)	$17 [$5-37] per month	Gamma (4·0, 17·1)		$19 [$11-30] per month	Gamma (15·4, 5·0)				
Treatment all phases (RR)	$103 [$58-161] per month	Gamma (15·4, 6·7)		$61 [$35-96] per month + $219 [$122-340]^ per episode of hospitalization	Gamma (15·4, 4·0) + Gamma (15·4, 14·3)	61	$4 [$2-6] per outpatient visit + $1019 [$579-1592]^ per episode of hospitalization	Gamma (15·4, 0·26) + Gamma (15·4, 66·2)	

*For the uncertainty distributions (columns 3, 6, and 9), the normal distribution is displayed with mean and standard deviation in parentheses, the beta distribution is displayed with alpha and beta in parentheses, where the mean of a beta distribution is equal to alpha divided by the sum of alpha and beta, the multinomial distribution is displayed with size and probability parameters in parentheses, and the gamma distribution is displayed with shape and scale parameters in parentheses.

#Rifampin-susceptible (RS) and rifampin-resistant (RR) standard of care (SOC) prices vary based on the country-specific prevalences of isoniazid and fluoroquinolone resistance and resistance testing practices for these drugs. No uncertainty was included apart from the prevalence of isoniazid/fluoroquinolone resistance and drug susceptibility testing.

^Treatment support voucher costs were subsequently subtracted out of RR-TB out-of-pocket + indirect costs for South Africa and the Philippines and out of RS-TB costs for India to avoid double-counting. Treatment support vouchers were not subtracted out of RR-TB costs for India to avoid underestimation, because the support vouchers are for food and food was not costed in Mullerpattan et al. 2020.

All costs are shown in 2021 USD. This table shows the cost per each cost component (i.e., each service/commodity) listed in the first column. These costs are assumed to be fixed across regimens. The quantities of these cost components required for each regimen are shown in Appendix Table 3. Both point estimates/means and uncertainty intervals have been estimated from the sources indicated in the "Source" column, unless otherwise noted.

Appendix Table 3: Quantities of treatment inputs and event probabilities in the cost analysis

Cost Components	Country	RS-TB SOC	RR-TB SOC	Pan-TB Oral	Pan-TB LAI	Individualized	Notes/Sources
**Quantities of inputs during treatment***							
Outpatient visits	India	7	9	5	5	21	-SOC: [62–66]<br>-Pan-TB oral and Individualized: scales with duration<br>-Pan-TB LAI: assume same as Pan-TB oral
	South Africa	7	9	5	5	21	
	Philippines	8	9	6	6	21	
Sputum smear microscopy	India	3	5	2	2	7	
	South Africa	4	7	3	3	7	
	Philippines	3	7	2	2	7	
Sputum Culture	India	0	3	0	0	7	
	South Africa	0	7	0	0	7	
	Philippines	0	7	0	0	7	
Chest Xray	India	0	3	0	0	7	
	South Africa	0	2	0	0	7	
	Philippines	0	2	0	0	7	
Liver function test (ALT, AST, bilirubin)	India	0	7	0	0	21	-SOC: [62,64–68].<br>-Pan-TB : no safety monitoring (same as RS SOC)<br>-Individualized : similar to RR SOC but scales with duration
	South Africa	0	7	0	0	21	
	Philippines	0	7	0	0	21	
Full blood count	India	0	7	0	0	21	
	South Africa	0	7	0	0	21	
	Philippines	0	7	0	0	21	
ECG	India	0	7	0	0	21	
	South Africa	0	7	0	0	21	
	Philippines	0	7	0	0	21	
Neuropathy screening	India	0	7	0	0	21	
	South Africa	0	7	0	0	21	
	Philippines	0	7	0	0	21	
Treatment support vouchers	India	6	6	3	0	18	-Pan-TB oral and Individualized: scales with duration<br>-Pan TB LAI: assume same as Pan-TB oral<br>-SOCs: RS-TB patients in South Africa & Philippines aren't eligible for support vouchers.
	South Africa	0	6	0	0	18	
	Philippines	0	6	0	0	18	
**Cumulative event probabilities [95% uncertainty interval]**							
Liver disfunction	Assumed to be the same across all countries	0·40% [0·11-0·87%]	3·5% [0·9-7·8%]	0·24% [0·07-0·51%]	0·24% [0·07-0·51%]	10·4% [2·4-24·0%]	-SOC RS-TB: [69–72]<br>-SOC RR-TB: [12,13,45,73]<br>-Pan-TB: same safety profile as HRZE from the TRPs was interpreted to mean same event frequency, but shorter duration yields fewer adverse events. The long-acting injectable Pan-TB regimen was assumed to have the same cumulative event probabilities as the oral Pan-TB regimen.<br>-Individualized: same weekly incidence as RR-TB SOC but longer duration
Pancreatitis		0%	2·0% [0·2-5·5%]	0%	0%	5·9% [0·8-16·1%]	
Anemia		0%	3·0% [0·6-7·1%]	0%	0%	8·9% [1·9-21·3%]	
Neutropenia		0%	4·0% [1·1-8·5%]	0%	0%	12·0% [3·3-26·5%]	

QTcF prolongation		0%	0·5% [0·1-1·3%]	0%	0%	1·5% [0·2-3·8%]	
Renal disfunction		0%	1·0% [0·2-2·4%]	0%	0%	2·9% [0·6-7·0%]	
Vision		0·30% [0·06-0·73%]	0%	0·18% [0·04-0·43%]	0·18% [0·04-0·43%]	0%	
Arthralgia		4·3% [1·2-9·2%]	0%	2·6% [0·8-5·5%]	2·6% [0·8-5·5%]	0%	
Short-term peripheral neuropathy		0%	27% [19-36%]	0%	0%	92% [60-100%]	
Long-term peripheral neuropathy		0%	5% [1·7-9·9%]	0%	0%	14% [5-29%]	
Hospitalization	India	1·0% [0·3-2·0%]	25% [19-31%]	1·0% [0·3-2·0%]	1·0% [0·3-2·0%]	25% [19-31%]	-RS and RR SOCs: [50,51]<br>-Pan: assumed same as RS SOC.<br>-Individualized: assumed same as RR SOC.
	South Africa	5·0% [2·4-8·4%]	30% [24-36%]	5·0% [2·4-8·4%]	5·0% [2·4-8·4%]	30% [24-36%]	
	Philippines	3·0% [2·2-4·0%]	6·9% [4·4-10·0%]	3·0% [2·2-4·0%]	3·0% [2·2-4·0%]	6·9% [4·4-10·0%]	

These quantities were multiplied by the unit costs in Appendix Table 2 to estimate total costs. “RS” = rifampin-susceptible; “RR” = rifampin-resistant; “SOC” = standard of care; “LAI” = long-acting injectable regimen. Both point estimates/means and uncertainty intervals have been estimated from the sources indicated in the “Notes/Sources” column, unless otherwise noted.

*At diagnosis, we also costed one outpatient visit and one Xpert MDR test for all patients.

Appendix Table 4: DALY and disability weight parameters

Health State	Disability Weight [95% uncertainty interval]	Distribution used for parameter sampling*	Sources/Notes
Active TB disease	0·33 [0·22-0·45]	Beta (21·2, 42·4)	[74]; assumed to be the same (per unit time) for both RS- and RR-TB
Post-TB disability	3·06 [2·02-4·43]	Beta (24·8, 0·12)	[75]; This disability weight is not shown per year; rather it is displayed cumulatively over the remaining lifespan of a TB survivor, and has also already been discounted. Post-TB disability included both post-TB mortality and post-TB sequellae and was assumed to be the same for all surviving patients, regardless of initial treatment outcome.
Renal disfunction	0·10 [0·07-0·15]	Beta (25·0, 215·5)	[74]; In the model, each weight was applied for 1 month per person experiencing each adverse event.
Pancreatitis	0·11 [0·08-0·16]	Beta (26·7, 208·7)	
Anemia	0·05 [0·03-0·08]	Beta (22·3, 406·1)	
Moderate vision impairment	0·03 [0·02-0·05]	Beta (15·9, 496·0)	
Arthralgia	0·12 [0·08-0·16]	Beta (26·8, 202·6)	
Peripheral neuropathy	0·13 [0·09-0·19]	Beta (24·4, 159·1)	[73,74]. In the model, this weight was applied for 3 months for people experiencing short-term peripheral neuropathy and for the lifetime of patients experiencing long-term peripheral neuropathy.
Neutropenia	0	Uncertainty not including in parameters equaling 0	Assumed to be asymptomatic based on [45].
QTcF prolongation	0	Uncertainty not including in parameters equaling 0	
Liver disfunction	0	Uncertainty not including in parameters equaling 0	

All disability weights are shown per year spent with each health state unless otherwise noted. Both point estimates/means and uncertainty intervals have been estimated from the sources indicated in the "Sources/Notes" column, unless otherwise noted.

*The beta distribution is displayed with alpha and beta in parentheses, where the mean of a beta distribution is equal to alpha divided by the sum of alpha and beta.

Appendix Table 5: CHEERS checklist for economic evaluation studies

Topic	No.	Item	Location where item is reported
**Title**	1	Identify the study as an economic evaluation and specify the interventions being compared.	Title
**Abstract**	2	Provide a structured summary that highlights context, key methods, results, and alternative analyses.	Abstract
		**Introduction**	
**Background and objectives**	3	Give the context for the study, the study question, and its practical relevance for decision making in policy or practice.	Introduction, paragraphs 1-4
		**Methods**	
**Health economic analysis plan**	4	Indicate whether a health economic analysis plan was developed and where available.	Not Applicable
**Study population**	5	Describe characteristics of the study population (such as age range, demographics, socioeconomic, or clinical characteristics).	Methods, paragraph 3
**Setting and location**	6	Provide relevant contextual information that may influence findings.	Methods, paragraph 2
**Comparators**	7	Describe the interventions or strategies being compared and why chosen.	Table 1; Methods paragraph 1
**Perspective**	8	State the perspective(s) adopted by the study and why chosen.	Methods, Outcomes subsection (paragraph 2)
**Time horizon**	9	State the time horizon for the study and why appropriate.	Methods, Outcomes subsection (paragraph 1)
**Discount rate**	10	Report the discount rate(s) and reason chosen.	Methods, Outcomes subsection (paragraph 3)
**Selection of outcomes**	11	Describe what outcomes were used as the measure(s) of benefit(s) and harm(s).	Methods, Outcomes subsection
**Measurement of outcomes**	12	Describe how outcomes used to capture benefit(s) and harm(s) were measured.	Methods, Patient Cohort Model subsection (and Methods Appendix)
**Valuation of outcomes**	13	Describe the population and methods used to measure and value outcomes.	Methods, Patient Cohort Model subsection (and Methods Appendix)
**Measurement and valuation of resources and costs**	14	Describe how costs were valued.	Methods, Outcomes subsection paragraph 2 (and Methods Appendix)
**Currency, price date, and conversion**	15	Report the dates of the estimated resource quantities and unit costs, plus the currency and year of conversion.	Methods, Outcomes subsection paragraph 2 (and Methods Appendix)
**Rationale and description of model**	16	If modelling is used, describe in detail and why used. Report if the model is publicly available and where it can be accessed.	Methods, Patient Cohort Model subsection (and Methods Appendix)
**Analytics and assumptions**	17	Describe any methods for analyzing or statistically transforming data, any extrapolation methods, and approaches for validating any model used.	Methods, Outcomes subsection paragraph 4
**Characterizing heterogeneity**	18	Describe any methods used for estimating how the results of the study vary for subgroups.	Not Applicable
**Characterizing distributional effects**	19	Describe how impacts are distributed across different individuals or adjustments made to reflect priority populations.	Not Applicable
**Characterizing uncertainty**	20	Describe methods to characterize any sources of uncertainty in the analysis.	Methods, Outcomes subsection paragraph 4 (and Appendix Tables 1-4)
**Approach to engagement with patients and others affected by the study**	21	Describe any approaches to engage patients or service recipients, the general public, communities, or stakeholders (such as clinicians or payers) in the design of the study.	Not Applicable
		**Results**	
**Study parameters**	22	Report all analytic inputs (such as values, ranges, references) including uncertainty or distributional assumptions.	Appendix Tables 1-4
**Summary of main results**	23	Report the mean values for the main categories of costs and outcomes of interest and summarize them in the most appropriate overall measure.	Results (paragraphs 1-8)

**Effect of uncertainty**	24	Describe how uncertainty about analytic judgments, inputs, or projections affect findings. Report the effect of choice of discount rate and time horizon, if applicable.	Results (paragraphs 1-9) and Appendix Results
**Effect of engagement with patients and others affected by the study**	25	Report on any difference patient/service recipient, general public, community, or stakeholder involvement made to the approach or findings of the study	Not Applicable
**Discussion**			
**Study findings, limitations, generalizability, and current knowledge**	26	Report key findings, limitations, ethical or equity considerations not captured, and how these could affect patients, policy, or practice.	Discussion paragraphs 1, 4, 5
**Other relevant information**			
**Source of funding**	27	Describe how the study was funded and any role of the funder in the identification, design, conduct, and reporting of the analysis	Methods, Role of the Funding Source subsection and Funding Statement
**Conflicts of interest**	28	Report authors conflicts of interest according to journal or International Committee of Medical Journal Editors requirements.	Declaration of Interests and submitted ICMJE forms

Appendix Text 4: One-Way and Additional Sensitivity Analyses

In addition to the main analyses of the Pan-TB oral regimen, we conducted one-way sensitivity analyses examining the sensitivity of results to specific characteristics of the Pan-TB regimen. These one-way sensitivity analyses were conducted by holding all characteristics fixed at the values in the main analysis (i.e., Appendix Table 1), and varying one characteristic at a time from a pessimistic value to an optimistic value (Appendix Table 6). The seven characteristics varied were Pan-TB regimen efficacy, duration, adherence, forgiveness, and safety; baseline prevalence of resistance to novel drugs in the Pan-TB regimen; and the trend in novel drug resistance over time (i.e., the population-level increase in the proportion of TB that is novel-resistant).

We included four additional sensitivity analyses that compared oral and injectable Pan-TB regimens and isolated improvements against the standards of care, each aimed at evaluating the sensitivity of results to specific model and scenario assumptions. Given uncertainty in the acquisition of novel drug resistance, we complemented the one-way sensitivity analysis of Pan-TB regimen characteristics with a scenario that included both 50% faster increases in novel drug resistance and a 50% higher baseline prevalence of novel resistance. We also compared outcomes to a version of the standard of care scenario in which the RS-TB regimen is only four months in duration, designed to represent wider scale up of the four-month isoniazid rifapentine moxifloxacin pyrazinamide (4HPMZ) regimen. To explore variation in the gap between clinical efficacy and programmatic effectiveness, we conducted targeted sensitivity analysis on adherence, forgiveness, and loss-to-follow-up.

Additionally, we estimated how cost-saving price thresholds would vary if the pan-TB regimen were instead compared to scenarios with improved RS-TB and RR-TB regimens, with these improved regimens priced the same as the standards of care.

A summary of the results of these analyses is presented in Appendix Text 5.

Appendix Table 6: Optimistic and pessimistic regimen characteristics used in one-way sensitivity analysis

Characteristics and additional details		Value in main analysis	Pessimistic value	Optimistic value
**Pan-TB regimen efficacy**		95% [93-97%]	90%	99%
**Pan-TB regimen duration***		3·5 months	6 months	2 months
**Pan-TB regimen ease of adherence**	% patients w/ < 70% adherence	14% [12-16%]	25%	5%
	% patients w/ 70-<90% adherence	34% [31-38%]	40%	18%
	% patients w/ ≥ 90% adherence	51% [47-55%]	35%	67%
**Pan-TB regimen forgiveness**		15%	10%	30%
**Pan-TB regimen safety^**	Liver disfunction	0·24% [0·07-0·51%]	1·9% [0·5-4·4%]	0·12% [0·04-0·25%]
	Pancreatitis	0%	1·1% [0·1-3·0%]	0%
	Anemia	0%	1·6% [0·3-3·9%]	0%
	Neutropenia	0%	2·2% [0·7-4·9%]	0%
	QTcF prolongation	0%	0·3% [0·1-0·7%]	0%
	Renal disfunction	0%	0·5% [0·1-1·3%]	0%
	Vision	0·18% [0·04-0·43%]	0%	0·09% [0·02-0·21%]
	Arthralgia	2·6% [0·8-5·5%]	0%	1·3% [0·4-2·8%]
	Short-term peripheral neuropathy	0%	17·0% [11·2-23·9%]	0%
	Long-term peripheral neuropathy	0%	5·0% [1·7-9·8%]	0%
	Liver function test (ALT, AST, bilirubin)	0	4	0
	Full blood count	0	4	0
	ECG	0	4	0
	Neuropathy Screening	0	4	0
**Baseline prevalence of resistance**	% RS-TB patients with novel-drug-resistant TB at baseline	0·2% [0·0-1·0%]	0·1%	0·4%
	% RR-TB patients with novel-drug-resistant TB at baseline, India	0·7% [0·4-1·4%]	0·4%	1·4%
	% RR-TB patients with novel-drug-resistant TB at baseline, South Africa	3·6% [2·8-4·6%]	1·8%	7·2%
	% RR-TB patients with novel-drug-resistant TB at baseline, Philippines	1·3% [0·9-2·0%]	0·7%	2·6%
**Barrier to resistance**	Increase per decade of 100% of patients using regimens with novel drugs	1 percentage point	0·5 percentage points	3 percentage points

* As in the main analysis, frequency of outpatient visits and monitoring; magnitude of patient-borne non-medical out-of-pocket and indirect costs; discontinuation during treatment; and cumulative incidence of adverse events were all assumed to scale with regimen duration.

^ Pessimistic weekly incidence of adverse events and safety monitoring were the same as with the rifampin-resistant standard of care (BPaL/BPaLM), but scaled by the shorter duration of the pan-TB regimen (3·5 months vs. 6 months). Optimistic safety was the same as the main analysis, but with half the weekly incidence of adverse event.

SUPPLEMENTARY APPENDIX REFERENCES

1 World Health Organization. Global Tuberculosis Report 2022. 2022; published online Oct. https://www.who.int/teams/global-tuberculosis-programme/tb-reports/global-tuberculosis-report-2022 (accessed Aug 24, 2023).

2 Timm J, Bateson A, Solanki P, *et al.* Baseline and acquired resistance to bedaquiline, linezolid and pretomanid, and impact on treatment outcomes in four tuberculosis clinical trials containing pretomanid. *PLOS Global Public Health* 2023; **3**: e0002283.

3 Ismail NA, Omar SV, Moultrie H, *et al.* Assessment of epidemiological and genetic characteristics and clinical outcomes of resistance to bedaquiline in patients treated for rifampicin-resistant tuberculosis: a cross-sectional and longitudinal study. *Lancet Infect Dis* 2022; **22**: 496–506.

4 Subbaraman R, Nathavitharana RR, Satyanarayana S, *et al.* The Tuberculosis Cascade of Care in India's Public Sector: A Systematic Review and Meta-analysis. *PLOS Medicine* 2016; **13**: e1002149.

5 Naidoo P, Theron G, Rangaka MX, *et al.* The South African Tuberculosis Care Cascade: Estimated Losses and Methodological Challenges. *The Journal of Infectious Diseases* 2017; **216**: S702–13.

6 Cox H, Dickson-Hall L, Ndjeka N, *et al.* Delays and loss to follow-up before treatment of drug-resistant tuberculosis following implementation of Xpert MTB/RIF in South Africa: A retrospective cohort study. *PLOS Medicine* 2017; **14**: e1002238.

7 Kruk ME, Schwalbe NR, Aguiar CA. Timing of default from tuberculosis treatment: a systematic review. *Trop Med Int Health* 2008; **13**: 703–12.

8 Cox H, Kebede Y, Allamuratova S, *et al.* Tuberculosis Recurrence and Mortality after Successful Treatment: Impact of Drug Resistance. *PLOS Medicine* 2006; **3**: e384.

9 He GX, Xie YG, Wang LX, *et al.* Follow-Up of Patients with Multidrug Resistant Tuberculosis Four Years after Standardized First-Line Drug Treatment. *PLOS ONE* 2010; **5**: e10799.

10 Gegia M, Winters N, Benedetti A, van Soolingen D, Menzies D. Treatment of isoniazid-resistant tuberculosis with first-line drugs: a systematic review and meta-analysis. *The Lancet Infectious Diseases* 2017; **17**: 223–34.

11 Conradie F, Diacon AH, Ngubane N, *et al.* Treatment of Highly Drug-Resistant Pulmonary Tuberculosis. *New England Journal of Medicine* 2020; **382**: 893–902.

12 Nyang'wa B-T, Berry C, Kazounis E, *et al.* A 24-Week, All-Oral Regimen for Rifampin-Resistant Tuberculosis. *New England Journal of Medicine* 2022; **387**: 2331–43.

13 Conradie F, Bagdasaryan TR, Borisov S, *et al.* Bedaquiline–Pretomanid–Linezolid Regimens for Drug-Resistant Tuberculosis. *New England Journal of Medicine* 2022; **387**: 810–23.

14 Collaborative Group for the Meta-Analysis of Individual Patient Data in MDR-TB treatment–2017, Ahmad N, Ahuja SD, *et al.* Treatment correlates of successful outcomes in pulmonary multidrug-resistant tuberculosis: an individual patient data meta-analysis. *Lancet* 2018; **392**: 821–34.

15 World Health Organization. Consolidated guidelines on tuberculosis: drug-susceptible tuberculosis treatment. 2022; published online May 24. https://www.who.int/publications-detail-redirect/9789240048126 (accessed March 16, 2023).

16 World Health Organization. Consolidated guidelines on tuberculosis: drug-resistant tuberculosis treatment, 2022 update. 2022; published online Dec 15. https://www.who.int/publications-detail-redirect/9789240063129 (accessed March 16, 2023).

17 World Health Organization. Target Regimen Profiles for Tuberculosis Treatment. 2023; published online Nov. https://www.who.int/publications-detail-redirect/9789240081512 (accessed Dec 7, 2023).

18 Stagg HR, Lewis JJ, Liu X, *et al.* Temporal Factors and Missed Doses of Tuberculosis Treatment. A Causal Associations Approach to Analyses of Digital Adherence Data. *Ann Am Thorac Soc* 2020; **17**: 438–49.

19 Maraba N, Orrell C, Chetty-Makkan CM, *et al.* Evaluation of adherence monitoring system using evriMED with a differentiated response compared to standard of care among drug-sensitive TB patients in three provinces in South Africa: a protocol for a cluster randomised control trial. *Trials* 2021; **22**: 389.

20 Liu X, Thompson J, Dong H, *et al.* Digital adherence technologies to improve tuberculosis treatment outcomes in China: a cluster-randomised superiority trial. *The Lancet Global Health* 2023; **11**: e693–703.

21 Imperial MZ, Nahid P, Phillips PPJ, *et al.* A patient-level pooled analysis of treatment-shortening regimens for drug-susceptible pulmonary tuberculosis. *Nat Med* 2018; **24**: 1708–15.

22 Garcia-Cremades M, Solans BP, Strydom N, *et al.* Emerging Therapeutics, Technologies, and Drug Development Strategies to Address Patient Nonadherence and Improve Tuberculosis Treatment. *Annu Rev Pharmacol Toxicol* 2022; **62**: 197–210.

23 World Health Organization. Life tables. 2020; published online Dec 6. https://www.who.int/data/gho/data/themes/topics/indicator-groups/indicator-group-details/GHO/gho-ghe-global-health-estimates-life-tables (accessed Aug 29, 2022).

24 Ma Y, Horsburgh CR, White LF, Jenkins HE. Quantifying TB transmission: a systematic review of reproduction number and serial interval estimates for tuberculosis. *Epidemiology & Infection* 2018; **146**: 1478–94.

25 Ochalek J, Lomas J, Claxton K. Estimating health opportunity costs in low-income and middle-income countries: a novel approach and evidence from cross-country data. *BMJ Global Health* 2018; **3**: e000964.

26 Zignol M, Cabibbe AM, Dean AS, *et al.* Genetic sequencing for surveillance of drug resistance in tuberculosis in highly endemic countries: a multi-country population-based surveillance study. *The Lancet Infectious Diseases* 2018; **18**: 675–83.

27 National Institute for Communicable Diseases. South African Tuberculosis Drug Resistance Survey. https://www.nicd.ac.za/assets/files/K-12750%20NICD%20National%20Survey%20Report_Dev_V11-LR.pdf (accessed June 28, 2023).

28 Indian Ministry of Health and Family Welfare, World Health Organization. Report of the First National Anti-Tuberculosis Drug Resistance Survey. 2018. https://tbcindia.gov.in/showfile.php?lid=3315 (accessed Feb 14, 2023).

29 Lim DR, Dean AS, Taguinod-Santiago MR, *et al.* Low prevalence of fluoroquinolone resistance among patients with tuberculosis in the Philippines: results of a national survey. *European Respiratory Journal* 2018; **51**. DOI:10.1183/13993003.02571-2017.

30 Hong Kong Chest Service. A controlled trial of 2-month, 3-month, and 12-month regimens of chemotherapy for sputum-smear-negative pulmonary tuberculosis. Results at 60 months. *Am Rev Respir Dis* 1984; **130**: 23–8.

31 Fox W. Whither short-course chemotherapy? *British Journal of Diseases of the Chest* 1981; **75**: 331–57.

32 Borgdorff MW, Sebek M, Geskus RB, Kremer K, Kalisvaart N, van Soolingen D. The incubation period distribution of tuberculosis estimated with a molecular epidemiological approach. *International Journal of Epidemiology* 2011; **40**: 964–70.

33 Menzies NA, Swartwood N, Testa C, *et al.* Time Since Infection and Risks of Future Disease for Individuals with Mycobacterium tuberculosis Infection in the United States. *Epidemiology* 2021; **32**: 70–8.

34 Statistics South Africa. Consumer Price Index data. 2022; published online Aug 24. https://www.statssa.gov.za/?page_id=1854 (accessed Aug 24, 2022).

35 Philippine Statistics Authority. OpenSTAT Database: Consumer Price Index. 2022; published online Jan 5. https://openstat.psa.gov.ph/PXWeb/pxweb/en/DB/DB__2M__PI__CPI__2012/?tablelist=true (accessed Aug 24, 2022).

36 India Ministry of Statistics and Programme Implementation. Inflation Data. 2022; published online Aug 24. https://www.mospi.gov.in/web/mospi/download-tables-data/-/reports/view/templateOne/16401?q=TBDCAT (accessed Aug 24, 2022).

37 International Monetary Fund. International Finance Statistics. 2022; published online Aug 23. https://data.imf.org/?sk=4C514D48-B6BA-49ED-8AB9-52B0C1A0179B (accessed Aug 24, 2022).

38 Neumann PJ, Ganiats TG, Russell LB, Sanders GD, Siegel JE, editors. Second Panel on Cost-Effectiveness in Health and Medicine. In: Cost-Effectiveness in Health and Medicine. Oxford University Press, 2016: 0.

39 Chatterjee S, Toshniwal MN, Bhide P, *et al.* Costs of TB services in India (No 1). *The International Journal of Tuberculosis and Lung Disease* 2021; **25**: 1013–8.

40 Sinanovic E, Ramma L, Vassall A, *et al.* Impact of reduced hospitalisation on the cost of treatment for drug-resistant tuberculosis in South Africa. *Int J Tuberc Lung Dis* 2015; **19**: 172–8.

41 Capeding TPJ, Rosa JD, Lam H, *et al.* Cost of TB prevention and treatment in the Philippines in 2017. *Int J Tuberc Lung Dis* 2022; **26**: 392–8.

42 FIND. GeneXpert® Negotiated Prices. 2022. https://www.finddx.org/pricing/genexpert/ (accessed Aug 31, 2022).

43 Vassall A, Siapka M, Foster N, *et al.* Cost-effectiveness of Xpert MTB/RIF for tuberculosis diagnosis in South Africa: a real-world cost analysis and economic evaluation. *Lancet Glob Health* 2017; **5**: e710–9.

44 South Africa National Health Laboratory Service. State Price List. 2018. https://paediatrics.org.za/wp-content/uploads/2023/05/NHLS-State-Price-List-2018.pdf (accessed Dec 16, 2021).

45 Sweeney S, Berry C, Kazounis E, *et al.* Cost-effectiveness of short, oral treatment regimens for rifampicin resistant tuberculosis. *PLOS Global Public Health* 2022; **2**: e0001337.

46 Schnippel K, Firnhaber C, Berhanu R, Page-Shipp L, Sinanovic E. Direct costs of managing adverse drug reactions during rifampicin-resistant tuberculosis treatment in South Africa. *The International Journal of Tuberculosis and Lung Disease* 2018; **22**: 393–8.

47 India Ministry of Health and Family Welfare. Nutrition Support to TB Patients (Nikshay Poshan Yojana). 2018. https://tbcindia.gov.in/WriteReadData/l892s/6851513623Nutrition%20support%20DBT%20Scheme%20details.pdf (accessed Aug 30, 2022).

48 South African Government. Disability grants. https://www.gov.za/services/social-benefits/disability-grant (accessed Aug 30, 2022).

49 The Global Fund. Philippines Global Fund 2020 tuberculosis funding request. 2020. https://data.theglobalfund.org/documents (accessed Aug 29, 2022).

50 WHO. TB Data. 2021; published online Oct 14. https://www.who.int/teams/global-tuberculosis-programme/data (accessed Aug 15, 2022).

51 Florentino JL, Arao RML, Garfin AMC, *et al.* Expansion of social protection is necessary towards zero catastrophic costs due to TB: The first national TB patient cost survey in the Philippines. *PLOS ONE* 2022; **17**: e0264689.

52 STOP TB Partnership. Global Drug Facility Products Catalog. 2023; published online Aug. https://www.stoptb.org/global-drug-facility-gdf/gdf-product-catalog (accessed Sept 11, 2023).

53 Chandra A, Kumar R, Kant S, Krishnan A. Costs of TB care incurred by adult patients with newly diagnosed drug-sensitive TB in Ballabgarh block in northern India. *Transactions of The Royal Society of Tropical Medicine and Hygiene* 2022; **116**: 63–9.

54 Foster N, Vassall A, Cleary S, Cunnama L, Churchyard G, Sinanovic E. The economic burden of TB diagnosis and treatment in South Africa. *Social Science & Medicine* 2015; **130**: 42–50.

55 Rupani MP, Cattamanchi A, Shete PB, Vollmer WM, Basu S, Dave JD. Costs incurred by patients with drug-susceptible pulmonary tuberculosis in semi-urban and rural settings of Western India. *Infect Dis Poverty* 2020; **9**: 144.

56 Sarin R, Vohra V, Singla N, Thomas BE, Krishnan R, Muniyandi M. Identifying costs contributing to catastrophic expenditure among TB patients registered under RNTCP in Delhi metro city in India - ClinicalKey. *Indian Journal of Tuberculosis* 2019; **66**: 150–7.

57 Muniyandi M, Thomas BE, Karikalan N, *et al.* Association of Tuberculosis With Household Catastrophic Expenditure in South India. *JAMA Network Open* 2020; **3**: e1920973.

58 Chimbindi N, Bor J, Newell M-L, *et al.* Time and money: the true costs of health care utilization for patients receiving ‘free’ HIV/TB care and treatment in rural KwaZulu-Natal. *J Acquir Immune Defic Syndr* 2015; **70**: e52–60.

59 Mudzengi D, Sweeney S, Hippner P, *et al.* The patient costs of care for those with TB and HIV: a cross-sectional study from South Africa. *Health Policy Plan* 2017; **32**: iv48–56.

60 Mullerpattan JB, Udwadia ZZ, Banka RA, Ganatra SR, Udwadia ZF. Catastrophic costs of treating drug resistant TB patients in a tertiary care hospital in India. *Indian Journal of Tuberculosis* 2019; **66**: 87–91.

61 Ramma L, Cox H, Wilkinson L, *et al.* Patients’ costs associated with seeking and accessing treatment for drug-resistant tuberculosis in South Africa. *Int J Tuberc Lung Dis* 2015; **19**: 1513–9.

62 India Ministry of Health and Family Welfare. Guidelines for programmatic management of drug resistance tuberculosis in India. 2021; published online March. https://tbcindia.gov.in/showfile.php?lid=3590 (accessed Aug 23, 2022).

63 World Health Organization India Office. Standards for TB care in India. 2014. https://tbcindia.gov.in/showfile.php?lid=3061 (accessed Aug 24, 2022).

64 Philippines National Tuberculosis Control Program. Manual of Procedures 6th Edition. 2022; published online March 3. https://ntp.doh.gov.ph/download/ntp-mop-6th-edition/ (accessed Aug 24, 2022).

65 South Africa National Department of Health. Management of rifampicin-resistance tuberculosis: a clinical reference guide. 2019; published online Nov. https://www.health.gov.za/wp-content/uploads/2020/11/management-of-rifampicin-resistant-tb-booklet-0220-v11.pdf (accessed Aug 24, 2022).

66 South Africa National Department of Health. National tuberculosis management guidelines. 2014. https://www.tbonline.info/media/uploads/documents/national_tuberculosis_management_guidelines_%282014%29.pdf (accessed Aug 24, 2022).

67 World Health Organization. Operational Handbook on Tuberculosis, Drug-Susceptible Tuberculosis Treatment. 2022; published online May 24. https://www.who.int/publications-detail-redirect/9789240050761 (accessed Aug 29, 2022).

68 World Health Organization. Operational Handbook on Tuberculosis, Drug-Resistant Tuberculosis Treatment. 2020; published online June 15. https://www.who.int/publications-detail-redirect/9789240006997 (accessed Aug 29, 2022).

69 Merle CS, Fielding K, Sow OB, *et al.* A Four-Month Gatifloxacin-Containing Regimen for Treating Tuberculosis. *New England Journal of Medicine* 2014; **371**: 1588–98.

70 Jindani A, Harrison TS, Nunn AJ, *et al.* High-Dose Rifapentine with Moxifloxacin for Pulmonary Tuberculosis. *N Engl J Med* 2014; **371**: 1599–608.

71 Scott JC, Shah N, Porco T, Flood J. Cost Resulting from Anti-Tuberculosis Drug Shortages in the United States: A Hypothetical Cohort Study. *PLoS One* 2015; **10**: e0134597.

72 Steele MA, Des Prez RM. The Role of Pyrazinamide in Tuberculosis Chemotherapy. *Chest* 1988; **94**: 845–50.

73 Conradie F, Diacon AH, Ngubane N, *et al.* Treatment of Highly Drug-Resistant Pulmonary Tuberculosis. *New England Journal of Medicine* 2020; **382**: 893–902.

74 Global Burden of Disease Collaborative Network. Global Burden of Disease Study 2019 (GBD 2019) Disability Weights. 2020. DOI:10.6069/1W19-VX76.

75 Menzies NA, Quaife M, Allwood BW, *et al.* Lifetime burden of disease due to incident tuberculosis: a global reappraisal including post-tuberculosis sequelae. *The Lancet Global Health* 2021; **9**: e1679–87.

76 Dorman SE, Nahid P, Kurbatova EV, *et al.* Four-Month Rifapentine Regimens with or without Moxifloxacin for Tuberculosis. *New England Journal of Medicine* 2021; **384**: 1705–18.

SUPPLEMENTARY RESULTS

Appendix Table 7: Health impact of Pan-TB and isolated improvement scenarios

Scenarios	India	Philippines	South Africa
**Average Annual Initial Cures (% of cohort)**			
Standard of Care	70.5% [58.3-79.7%]	70.7% [58.4-80.0%]	68.9% [57.0-77.9%]
Pan-TB TRP: Oral	76.3% [68.4-83.4%]	76.2% [68.4-83.3%]	75.4% [67.6-82.5%]
Pan-TB Oral, with DST	76.3% [68.4-83.4%]	76.3% [68.4-83.3%]	75.5% [67.7-82.5%]
Pan-TB TRP: LAI	82.4% [77.3-88.0%]	82.4% [77.3-88.0%]	82.3% [77.3-88.0%]
Improved RS Regimen	75.2% [67.4-82.2%]	75.5% [67.7-82.5%]	74.1% [66.3-81.1%]
Improved RR Regimen	70.6% [58.4-79.8%]	70.8% [58.5-80.1%]	69.1% [57.3-78.1%]
Improved RR Retention	70.7% [58.5-79.9%]	70.8% [58.5-80.1%]	69.3% [57.3-78.3%]
Improved RR Assignment	70.9% [58.6-80.1%]	71.0% [58.7-80.4%]	69.2% [57.3-78.3%]
**Change in Cumulative Initial Cures (% increase vs. SOC)**			
Pan-TB TRP: Oral	6.1% [2.3-14.1%]	5.5% [1.9-13.1%]	7.1% [3.2-15.3%]
Pan-TB Oral, with DST	6.1% [2.4-14.1%]	5.5% [1.9-13.1%]	7.2% [3.3-15.3%]
Pan-TB TRP: LAI	12.2% [4.4-28.1%]	11.2% [3.8-26.6%]	14.3% [6.1-30.9%]
Improved RS Regimen	5.1% [1.4-12.9%]	4.8% [1.3-12.3%]	5.8% [2.0-13.7%]
Improved RR Regimen	0.1% [0.0- 0.2%]	0.1% [0.0- 0.1%]	0.2% [0.1- 0.5%]
Improved RR Retention	0.1% [0.1- 0.3%]	0.1% [0.0- 0.2%]	0.3% [0.1- 0.6%]
Improved RR Assignment	0.4% [0.2- 0.7%]	0.3% [0.2- 0.5%]	0.4% [0.1- 0.6%]
**Average Annual Cures After Possible Retreatment (% of cohort)**			
Standard of Care	83.0% [71.9-90.0%]	85.6% [74.9-92.2%]	82.2% [71.4-89.3%]
Pan-TB TRP: Oral	88.1% [81.8-92.7%]	90.2% [84.2-94.3%]	87.8% [81.5-92.5%]
Pan-TB Oral, with DST	88.1% [81.8-92.7%]	90.2% [84.2-94.3%]	87.9% [81.5-92.5%]
Pan-TB TRP: LAI	93.1% [90.5-95.5%]	94.7% [92.4-96.7%]	93.2% [90.3-95.8%]
Improved RS Regimen	87.1% [80.8-91.7%]	89.5% [83.5-93.6%]	86.6% [80.2-91.3%]
Improved RR Regimen	83.1% [72.0-90.1%]	85.7% [75.0-92.3%]	82.5% [71.7-89.5%]
Improved RR Retention	83.2% [72.0-90.2%]	85.7% [75.0-92.3%]	82.6% [71.7-89.6%]
Improved RR Assignment	83.4% [72.3-90.5%]	86.0% [75.3-92.6%]	82.6% [71.7-89.6%]
**Change in Cumulative Eventual Cures (% increase vs. SOC)**			
Pan-TB TRP: Oral	4.0% [1.3-10.3%]	2.9% [0.7- 8.1%]	4.4% [1.6-10.4%]
Pan-TB Oral, with DST	4.0% [1.3-10.3%]	2.9% [0.7- 8.1%]	4.4% [1.6-10.5%]
Pan-TB TRP: LAI	7.5% [2.2-18.9%]	5.3% [1.1-14.6%]	8.2% [2.8-19.4%]
Improved RS Regimen	3.3% [0.7- 9.4%]	2.5% [0.3- 7.5%]	3.5% [0.9- 9.3%]
Improved RR Regimen	0.1% [0.0- 0.2%]	0.1% [0.0- 0.1%]	0.2% [0.1- 0.4%]
Improved RR Retention	0.1% [0.0- 0.2%]	0.0% [0.0- 0.1%]	0.2% [0.1- 0.4%]
Improved RR Assignment	0.5% [0.3- 0.7%]	0.3% [0.2- 0.5%]	0.4% [0.1- 0.6%]
**Cumulative TB Deaths (hundred thousands)**			
Standard of Care	29.7 [17.5-48.9]	3.0 [1.6- 5.1]	2.4 [1.4- 3.9]
Pan-TB TRP: Oral	20.6 [12.9-31.1]	2.0 [1.2- 3.1]	1.7 [1.0- 2.5]
Pan-TB Oral, with DST	20.5 [12.9-31.1]	2.0 [1.2- 3.1]	1.7 [1.0- 2.5]
Pan-TB TRP: LAI	11.9 [7.9-16.7]	1.0 [0.7- 1.5]	0.9 [0.6- 1.4]
Improved RS Regimen	22.2 [14.5-32.7]	2.1 [1.3- 3.3]	1.8 [1.2- 2.7]
Improved RR Regimen	29.5 [17.4-48.7]	2.9 [1.6- 5.1]	2.4 [1.4- 3.9]
Improved RR Retention	29.5 [17.3-48.5]	2.9 [1.6- 5.1]	2.4 [1.4- 3.9]
Improved RR Assignment	29.0 [16.8-48.1]	2.9 [1.5- 5.0]	2.4 [1.4- 3.9]
**Average Annual TB Deaths (per 100 index diagnoses)**			
Standard of Care	16.0 [9.4-26.2]	10.3 [5.6-17.8]	15.5 [9.2-25.0]
Pan-TB TRP: Oral	11.3 [7.1-17.3]	7.1 [4.2-11.4]	10.9 [6.6-16.7]
Pan-TB Oral, with DST	11.3 [7.0-17.3]	7.1 [4.2-11.4]	10.8 [6.6-16.7]
Pan-TB TRP: LAI	6.7 [4.4- 9.4]	3.8 [2.5- 5.5]	6.3 [3.8- 9.3]
Improved RS Regimen	12.2 [7.9-18.1]	7.5 [4.6-11.9]	11.8 [7.5-17.7]
Improved RR Regimen	15.9 [9.3-26.1]	10.2 [5.6-17.8]	15.3 [9.1-24.8]
Improved RR Retention	15.8 [9.3-26.1]	10.2 [5.6-17.8]	15.3 [9.0-24.8]
Improved RR Assignment	15.6 [9.0-25.8]	10.0 [5.4-17.6]	15.2 [8.9-24.8]
**Change in Cumulative TB Deaths (% decline vs. SOC)**			
Pan-TB TRP: Oral	29.8% [19.9-40.9%]	31.8% [20.9-43.2%]	30.9% [22.0-41.0%]
Pan-TB Oral, with DST	30.0% [20.2-40.9%]	31.9% [21.1-43.2%]	31.1% [22.4-41.1%]
Pan-TB TRP: LAI	57.9% [37.9-76.4%]	62.9% [42.4-80.2%]	60.2% [42.5-77.1%]
Improved RS Regimen	24.0% [12.1-37.0%]	27.0% [14.5-40.1%]	24.3% [14.0-36.2%]
Improved RR Regimen	0.7% [0.3- 1.2%]	0.4% [0.2- 0.8%]	1.3% [0.6- 2.3%]
Improved RR Retention	0.9% [0.3- 1.9%]	0.5% [0.1- 1.1%]	1.6% [0.6- 3.1%]
Improved RR Assignment	2.8% [1.2- 5.1%]	2.7% [1.1- 5.2%]	2.1% [0.7- 4.2%]
**Cumulative Secondary Cases (hundreds of thousands)**			

Standard of Care	14.1 [8.8-19.9]	2.3 [1.3- 3.4]	1.1 [0.6- 1.6]
Pan-TB TRP: Oral	11.7 [7.3-16.1]	1.9 [1.1- 2.7]	0.9 [0.5- 1.2]
Pan-TB Oral, with DST	11.7 [7.3-16.1]	1.9 [1.1- 2.7]	0.9 [0.5- 1.2]
Pan-TB TRP: LAI	9.3 [5.6-13.1]	1.5 [0.8- 2.2]	0.7 [0.4- 0.9]
Improved RS Regimen	12.2 [7.7-16.6]	2.0 [1.1- 2.8]	0.9 [0.6- 1.3]
Improved RR Regimen	14.0 [8.7-19.9]	2.3 [1.3- 3.4]	1.1 [0.6- 1.6]
Improved RR Retention	13.9 [8.6-19.8]	2.3 [1.3- 3.4]	1.1 [0.6- 1.6]
Improved RR Assignment	14.0 [8.7-19.8]	2.3 [1.3- 3.4]	1.1 [0.6- 1.6]
**Average Annual Secondary Cases (per 100 index diagnoses)**			
Standard of Care	7.6 [4.7-10.7]	8.1 [4.5-11.8]	6.9 [4.1-10.0]
Pan-TB TRP: Oral	6.4 [4.0- 8.9]	6.8 [3.8- 9.8]	5.6 [3.4- 8.1]
Pan-TB Oral, with DST	6.4 [4.0- 8.9]	6.8 [3.8- 9.8]	5.6 [3.4- 8.1]
Pan-TB TRP: LAI	5.2 [3.1- 7.4]	5.4 [2.9- 7.9]	4.4 [2.5- 6.4]
Improved RS Regimen	6.6 [4.2- 9.2]	7.0 [3.9-10.0]	5.9 [3.6- 8.5]
Improved RR Regimen	7.5 [4.7-10.7]	8.0 [4.4-11.8]	6.8 [4.1-10.0]
Improved RR Retention	7.5 [4.6-10.6]	8.0 [4.4-11.8]	6.8 [4.1- 9.9]
Improved RR Assignment	7.5 [4.7-10.7]	8.0 [4.4-11.8]	6.8 [4.1-10.0]
**Change in Cumulative Secondary Cases (% decline vs. SOC)**			
Pan-TB TRP: Oral	16.7% [9.1-26.9%]	17.1% [9.0-27.6%]	19.7% [12.4-29.1%]
Pan-TB Oral, with DST	16.8% [9.1-26.9%]	17.1% [9.1-27.6%]	19.8% [12.5-29.1%]
Pan-TB TRP: LAI	33.2% [17.3-53.5%]	35.0% [18.2-55.6%]	39.0% [23.8-57.9%]
Improved RS Regimen	13.3% [5.5-23.9%]	14.7% [6.4-25.6%]	15.1% [7.7-25.0%]
Improved RR Regimen	0.3% [0.1- 0.5%]	0.2% [0.1- 0.3%]	0.6% [0.3- 1.1%]
Improved RR Retention	1.0% [0.4- 1.9%]	0.5% [0.2- 1.0%]	1.7% [0.7- 3.2%]
Improved RR Assignment	0.7% [0.0- 1.7%]	0.7% [0.1- 1.5%]	0.7% [0.1- 1.6%]
**Incidence (per 100,000, at year 10)**			
Standard of Care	204 [178-230]	535 [251-811]	561 [379-741]
Pan-TB TRP: Oral	196 [170-221]	511 [241-776]	536 [362-709]
Pan-TB Oral, with DST	196 [170-221]	511 [241-776]	536 [362-709]
Pan-TB TRP: LAI	187 [161-214]	487 [229-746]	512 [344-680]
Improved RS Regimen	197 [172-223]	514 [243-782]	542 [366-716]
Improved RR Regimen	204 [178-229]	535 [251-810]	560 [379-740]
Improved RR Retention	203 [177-229]	534 [251-809]	559 [378-738]
Improved RR Assignment	204 [177-229]	534 [250-809]	560 [379-740]
**Change in Incidence (% decline vs. SOC at year 10)**			
Pan-TB TRP: Oral	4.1% [1.8- 7.9%]	4.5% [1.8- 8.8%]	4.4% [2.2- 8.1%]
Pan-TB Oral, with DST	4.1% [1.9- 7.9%]	4.5% [1.8- 8.8%]	4.4% [2.2- 8.1%]
Pan-TB TRP: LAI	8.1% [3.5-15.4%]	9.1% [3.6-17.5%]	8.7% [4.1-15.8%]
Improved RS Regimen	3.3% [1.1- 7.0%]	3.8% [1.2- 8.2%]	3.4% [1.3- 7.0%]
Improved RR Regimen	0.1% [0.0- 0.1%]	0.0% [0.0- 0.1%]	0.1% [0.1- 0.3%]
Improved RR Retention	0.2% [0.1- 0.4%]	0.1% [0.0- 0.3%]	0.4% [0.2- 0.7%]
Improved RR Assignment	0.2% [0.0- 0.4%]	0.2% [0.0- 0.4%]	0.2% [0.0- 0.4%]

Table shows outcome ratios, absolute outcomes, and incremental outcomes under the Pan-TB and isolated improvement scenarios. Initial cures = newly diagnosed TB patients cured after 1 round of treatment; Eventual cures = newly diagnosed TB patients cured after initial round of treatment and possible retreatment; TB deaths include post-diagnosis deaths in the index patient and all deaths in the secondary cases they generate after diagnosis. All cumulative outcomes are shown over a 10-year horizon, all annual outcomes are averaged over 10 years. “TRP” = target regimen profile; “LAI” = long-acting injectable; “RS” = rifampin-susceptible; “RR” = rifampin-resistant; “DST” = drug-susceptibility testing.

Appendix Figure 5: Projected reductions in TB incidence from Pan-TB regimens and other regimen improvements

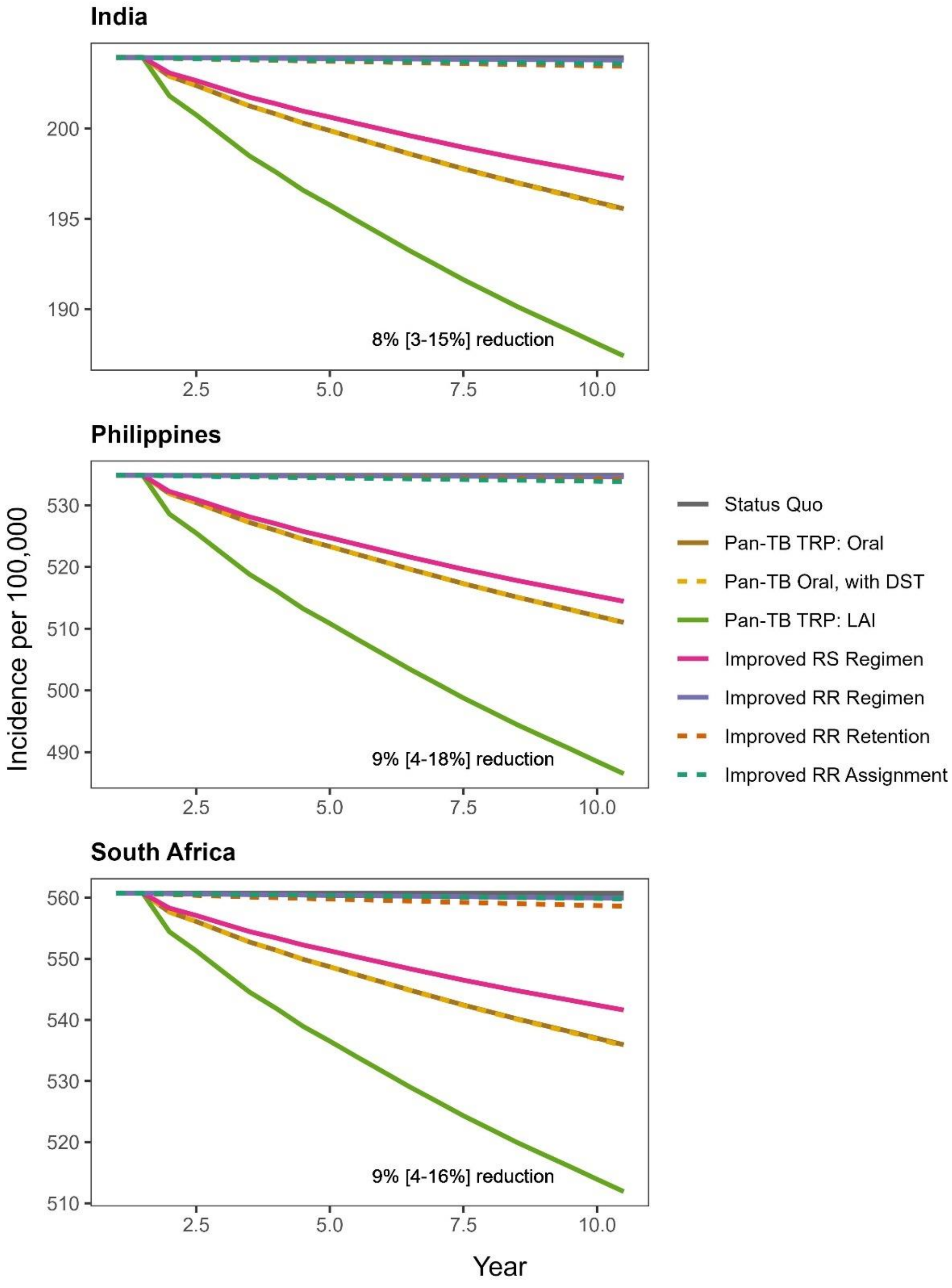

Figure shows estimated reductions in incidence under the Pan-TB and isolated improvement scenarios, assuming flat trends in incidence under the standard of care. Note the y-axis does not extend to zero and scale varies by country. Text labels on each panel indicate the cumulative reduction in incidence at year 10 under the Pan-TB long-acting injectable scenario (solid green line, with 95% uncertainty intervals reported in brackets). “TRP” = target regimen profile; “LAI” = long-acting injectable; “RS” = rifampin-susceptible; “RR” = rifampin-resistant.

Appendix Table 8: Incremental health impact of Pan-TB Scenario vs. Improved Rifampin-Susceptible Regimen Scenario

Outcome (all presented as % changes)	India	Philippines	South Africa
Increase in cumulative initial cures	1.0% [0.7-1.2%]	0.7% [0.4-0.9%]	1.3% [0.9-1.7%]
Increase in cumulative eventual cures	0.7% [0.4-1.0%]	0.5% [0.2-0.7%]	0.9% [0.5-1.3%]
Decline in cumulative TB deaths	7.6% [4.7-11.5%]	6.4% [3.4-10.5%]	8.7% [5.5-12.9%]
Decline in cumulative secondary cases	3.9% [2.5-5.9%]	2.8% [1.6-4.6%]	5.4% [3.5-8.0%]

Table shows percent increases in total cures and percent decreases in cumulative deaths and secondary cases under the Pan-TB TRP scenario, compared to the scenario with an improved rifampin-susceptible regimen. Initial cures = newly diagnosed TB patients cured after 1 round of treatment; Eventual cures = newly diagnosed TB patients cured after initial round of treatment and possible retreatment; TB deaths include post-diagnosis deaths in the index patient and all deaths in the secondary cases they generate after diagnosis. All cumulative outcomes are shown over a 10-year horizon.

Appendix Figure 6: Initial durable cures, by drug resistance phenotype, country, and scenario

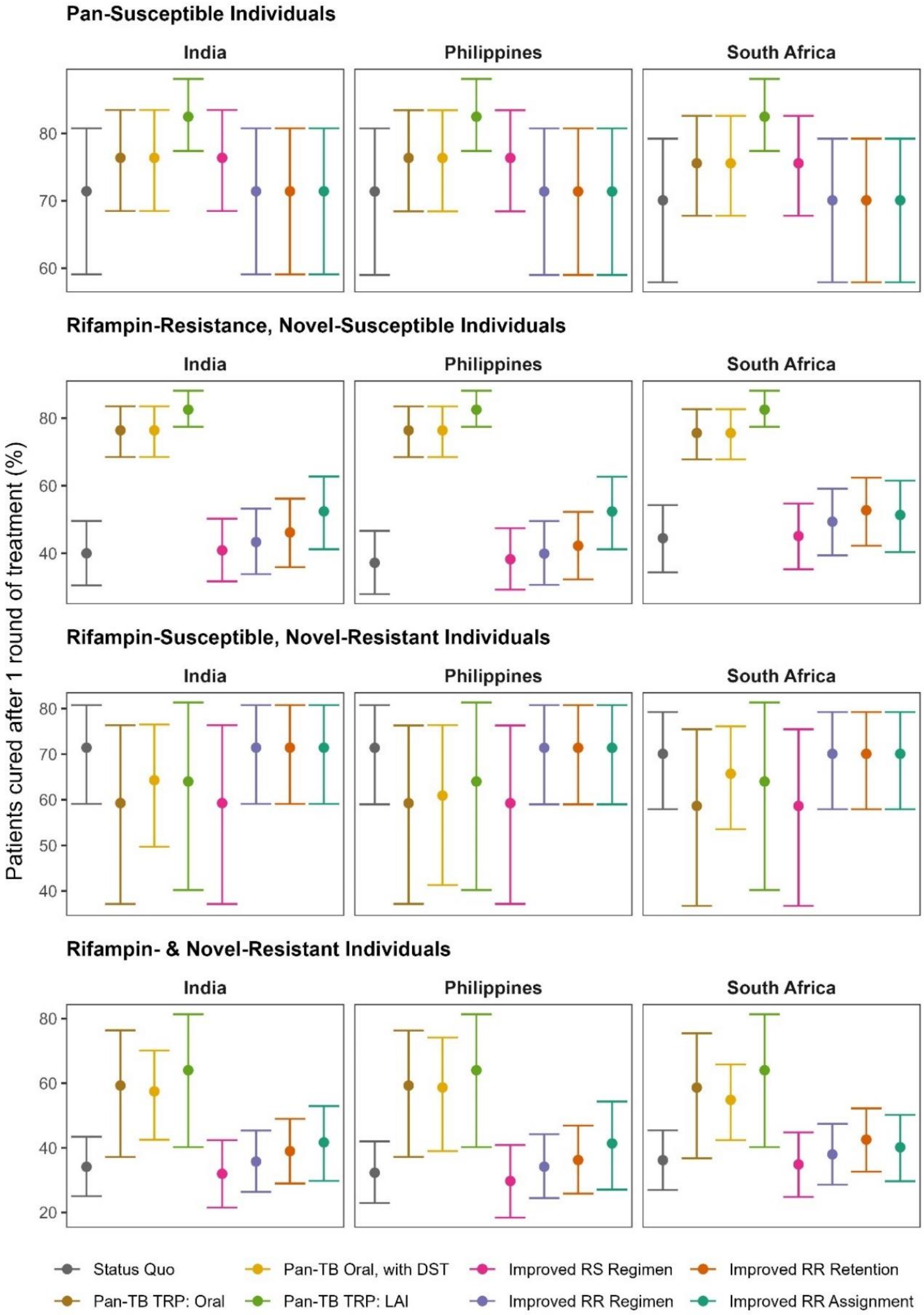

Figure shows the proportion of patients, by drug resistance phenotype, cured in each country and scenario after one round of treatment. Proportions are calculated among all patients newly diagnosed over the 10 year model period.

Error bars indicate 95% uncertainty intervals. “TRP” = target regimen profile; “LAI” = long-acting injectable; “RS” = rifampin-susceptible; “RR” = rifampin-resistant; “DST” = drug-susceptibility testing. Note that although efficacy is higher with the individualized regimen than the Pan-TB regimen for people with TB that is both rifampin- and novel-resistant, in many simulations the overall probability of cure was lower, due to the individualized regimen’s assumed longer duration and poorer adherence; this led to lower probability of cure for people with rifampin- and novel-resistant TB in many simulations when confirmatory DST was added to the Pan-TB regimen scenario (i.e., comparing yellow vs. brown in the bottom panel of the figure).

Appendix Figure 7: Costs per person by cost category, country, and scenario

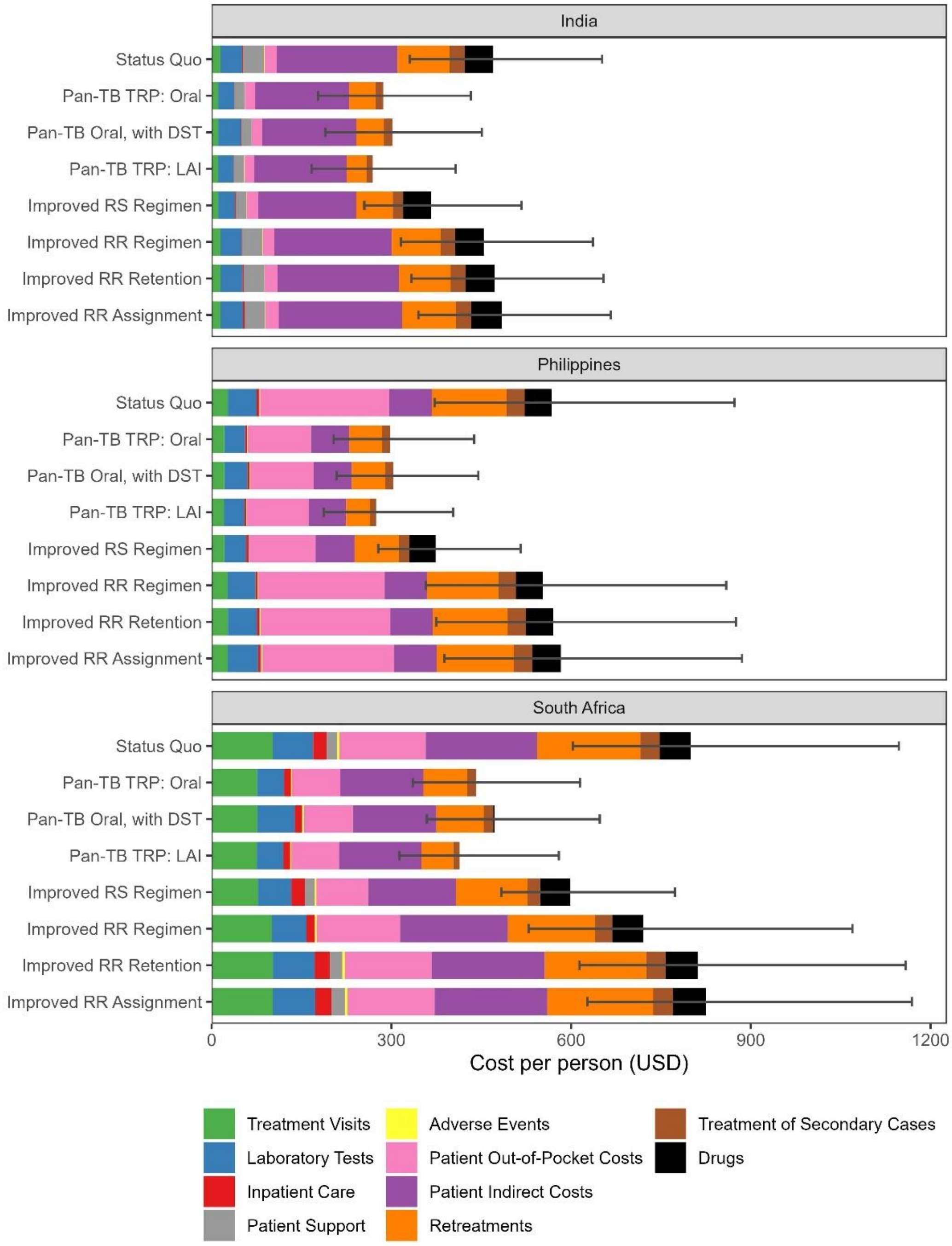

Costs are shown as per-person averages over 10 years. A short-term cost analysis would exclude retreatment and secondary case treatment costs (orange and brown bars), while a health systems perspective cost analysis would exclude patient-borne costs (pink and purple bars). Pan-TB drug costs (black bars) are treated as unknown and thus excluded from this figure in the analyses of Pan-TB regimens (except in the Pan-TB oral with DST scenario, in which a small number of patients with novel-resistant TB receive standard of care regimens). In all other analyses, drugs are costed the same as the standard of care regimens for treating RS-TB and RR-TB (mostly 6HRZE and BPaL[M]). Error bars indicate 95% uncertainty intervals. "TRP" = target regimen profile; "LAI" = long-acting injectable; "RS" = rifampin-susceptible; "RR" = rifampin-resistant; "DST" = drug-susceptibility testing; "USD" = United States Dollars.

Appendix Figure 8: Cost-saving price thresholds of Pan-TB regimens under alternative comparators

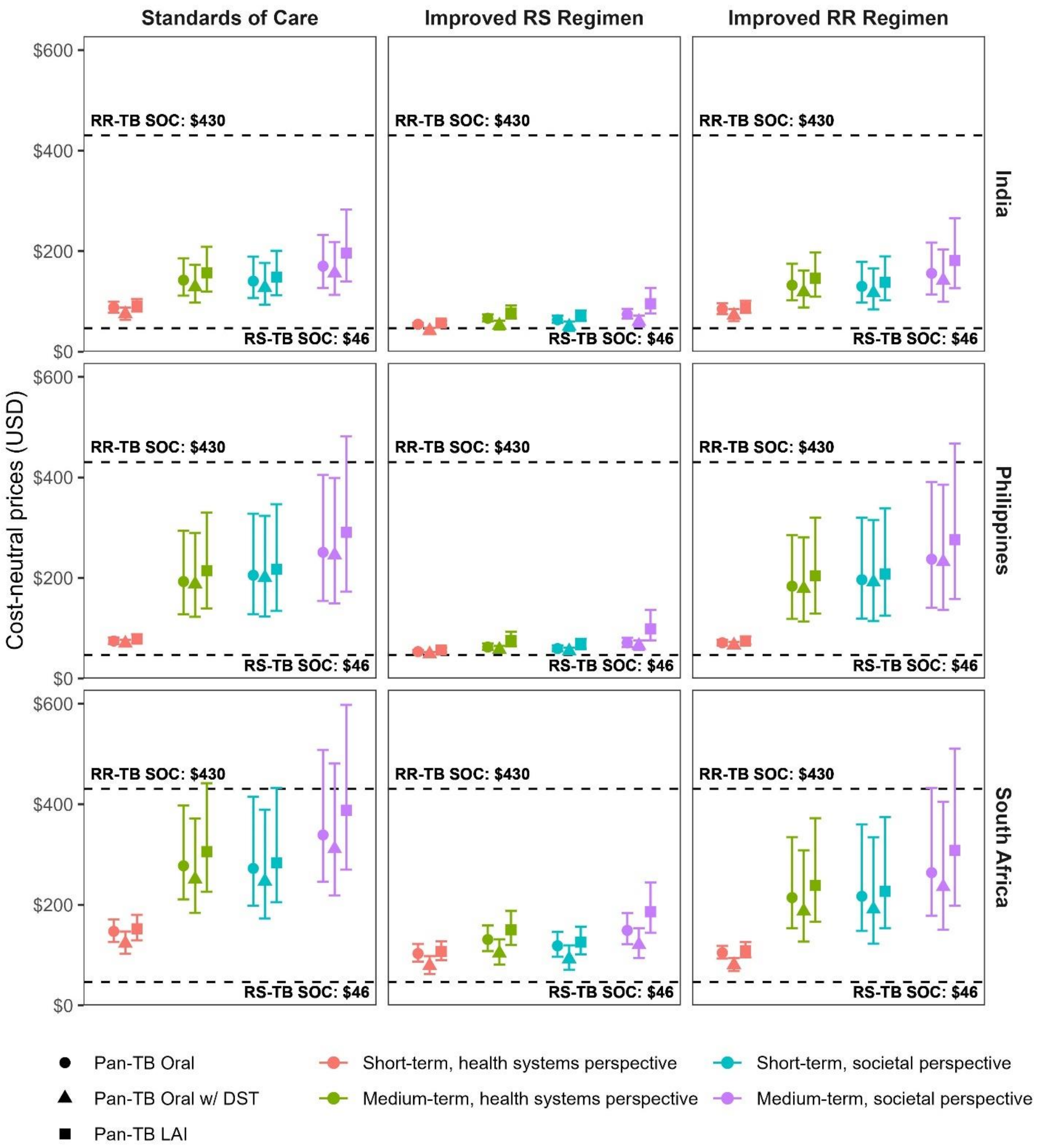

Figure shows the prices below which the Pan-TB regimens would be cost-saving compared to the standards of care (left column), compared to the isolated improvements scenario with an improved RS-TB regimen (costing the same as the RS-TB standard of care) and the RR-TB standard of care (middle column), and compared to the isolated improvements scenario with an improved RR-TB regimen (costing the same as the RR-TB standard of care) and the RS-TB standard of care (right column). Cost-saving price thresholds vary by country, regimen (either a 2-month oral regimen or a long-acting injectable regimen/"LAI"), horizon (short-term incorporating costs during a patient's

treatment course only or medium-term incorporating averted costs of retreatments and secondary case treatments over 10 years), and perspective (health systems perspective that omits patient-borne non-medical costs or societal perspective that includes them). Results are presented in 2021 United States Dollars ("USD"). Current prices of the rifampin-susceptible (RS) and rifampin-resistant (RR) standards of care are shown as dashed horizontal lines on each plot. Error bars indicate 95% uncertainty intervals.

Appendix Figure 9: Uncertainty in cost-saving price thresholds

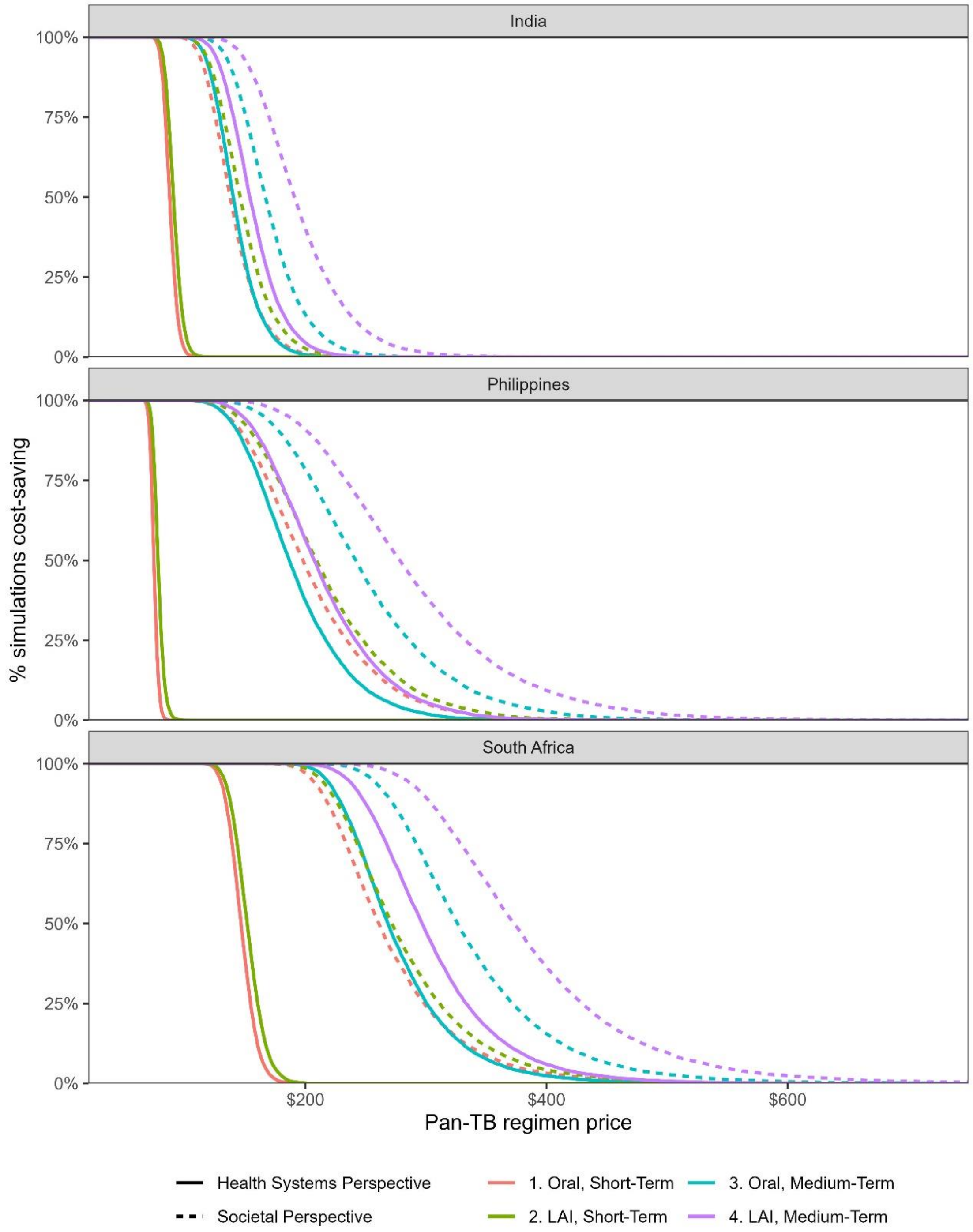

Figure shows the percentage of the 10,000 simulations (each representing a different sample from the parameter uncertainty distributions) for which the modeled Pan-TB regimens (either oral or one-time long-acting injectable/"LAI") would be considered cost-saving at a range of hypothetical prices (x-axis), under both short-term (considering costs of treating primary patients only) and medium-term (considering also the costs of retreatments and treatment of secondary cases) time horizons and both health systems (considering medical costs only) and societal (considering also non-medical out-of-pocket and indirect costs borne by patients) perspectives.

Appendix Figure 10: DALYs per person by type, country, and scenario

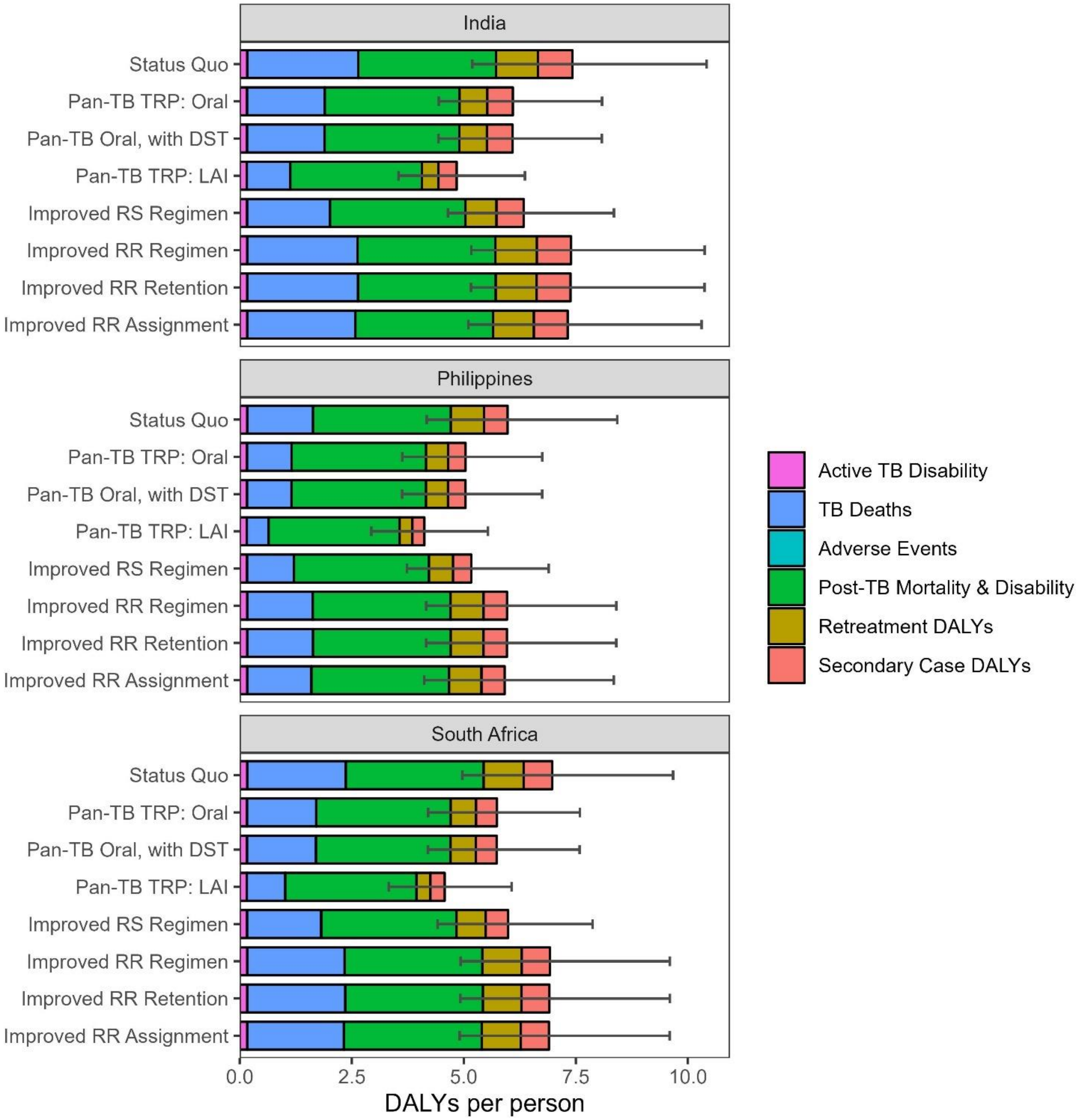

Disability-adjusted life years (DALYs) are shown as per-person averages over 10 years. Retreatment DALYs include active TB disability, TB deaths, and adverse events that accrue during or after a second course of treatment. Secondary case DALYs include active TB disability, TB mortality, adverse events, post-TB mortality and disability, and retreatment DALYs that occur among secondary cases. Adverse event DALYs accrued are small compared to other DALYs accrued, and are thus not visible in the figure. Error bars indicate 95% uncertainty intervals. "TRP" = target regimen profile; "LAI" = long-acting injectable; "RS" = rifampin-susceptible; "RR" = rifampin-resistant; "DST" = drug-susceptibility testing.

Appendix Figure 11: Cost-effective price thresholds for a Pan-TB regimen

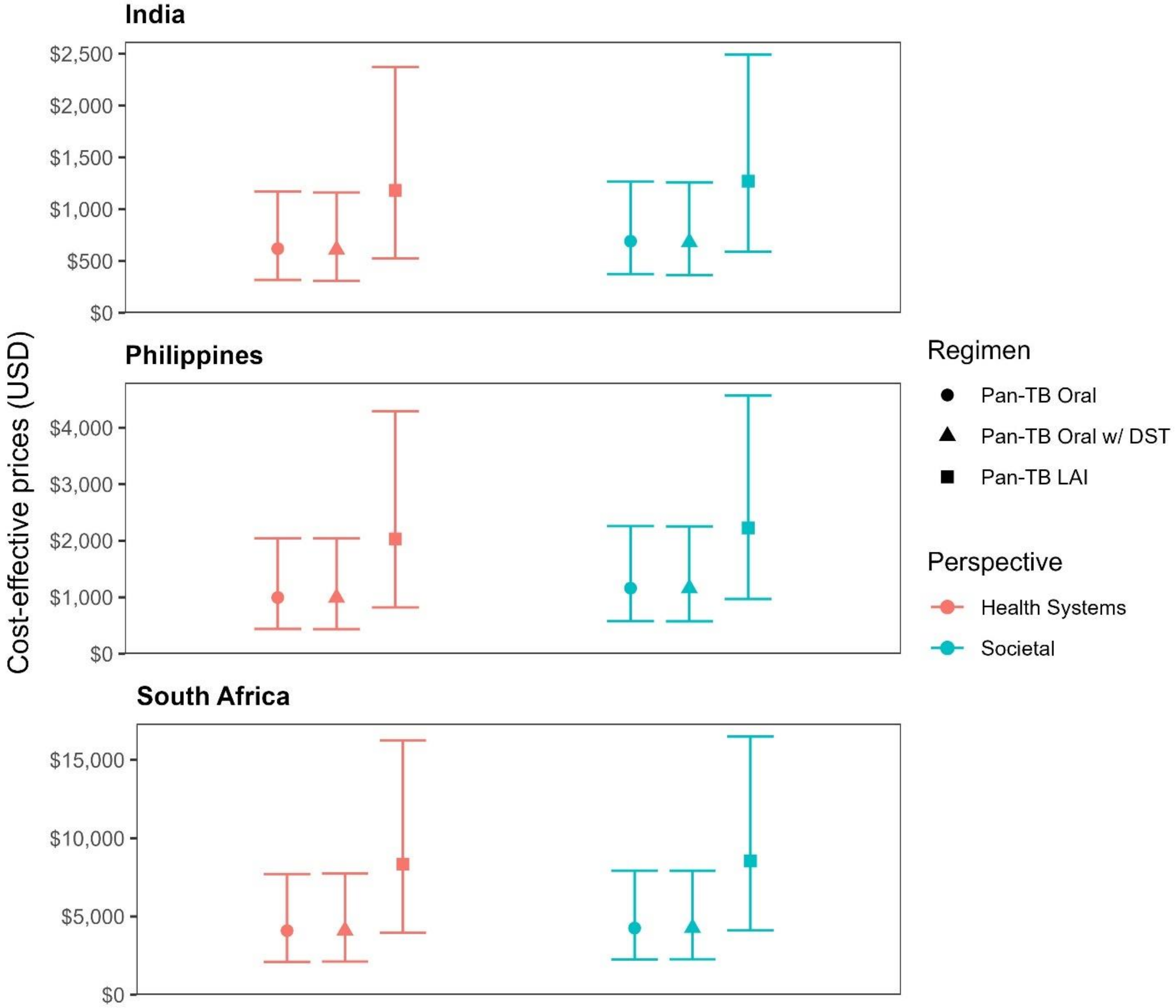

Figure shows the prices at which a Pan-TB regimen would be cost-effective compared to the standard of care. Cost-effective prices vary by country, regimen (a 3·5-month oral regimen, with or without confirmatory DST, or a long-acting injectable regimen/"LAI"), and perspective (health systems perspective that omits patient-borne non-medical costs or societal perspective that includes them). Results are presented in 2021 United States Dollars ("USD"). Cost-effective prices were based on willingness-to-pay thresholds that varied by country: $430 in India, $1060 in the Philippines, and $3400 in South Africa.[25] Error bars indicate 95% uncertainty intervals. Note that y-axes vary by panel.

Appendix Figure 12: Sensitivity of cost-effective prices to willingness-to-pay

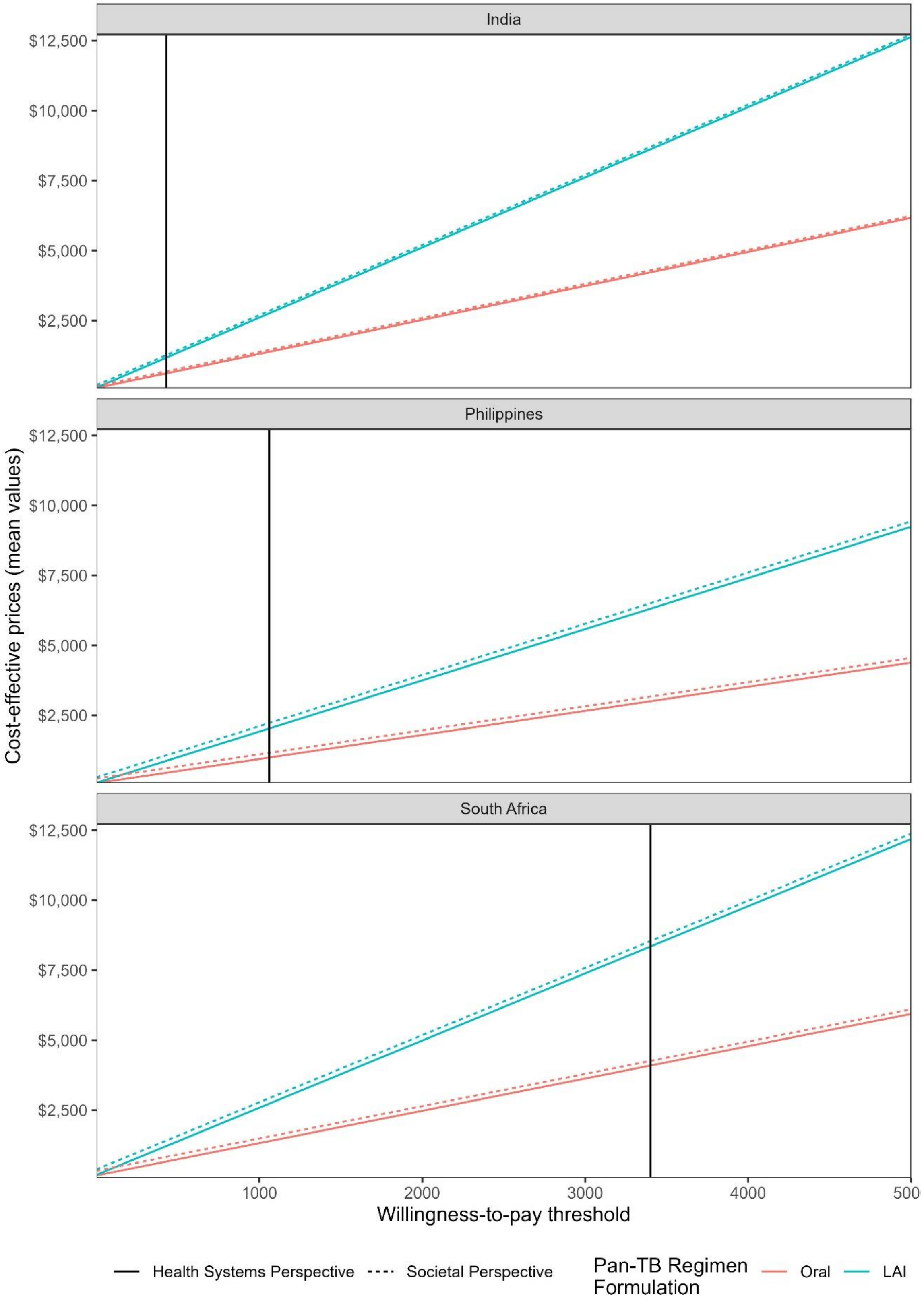

Figure shows the mean (across 10,000 parameter set samples) prices at which the oral and one-time long-acting injectable ("LAI") pan-TB regimens would be considered cost-effective across a range of willingness-to-pay thresholds (x-axis), under both health systems (considering medical costs only) and societal (also considering non-medical out-of-pocket and indirect costs borne by patients) perspectives. Black vertical lines indicate the willingness-to-pay thresholds used in the main analysis of cost-effective prices ($430 in India, $1060 in the Philippines, and $3400 in South Africa).

Appendix Figure 13: Effects of varying oral pan-TB regimen characteristics on initial durable cures ranges

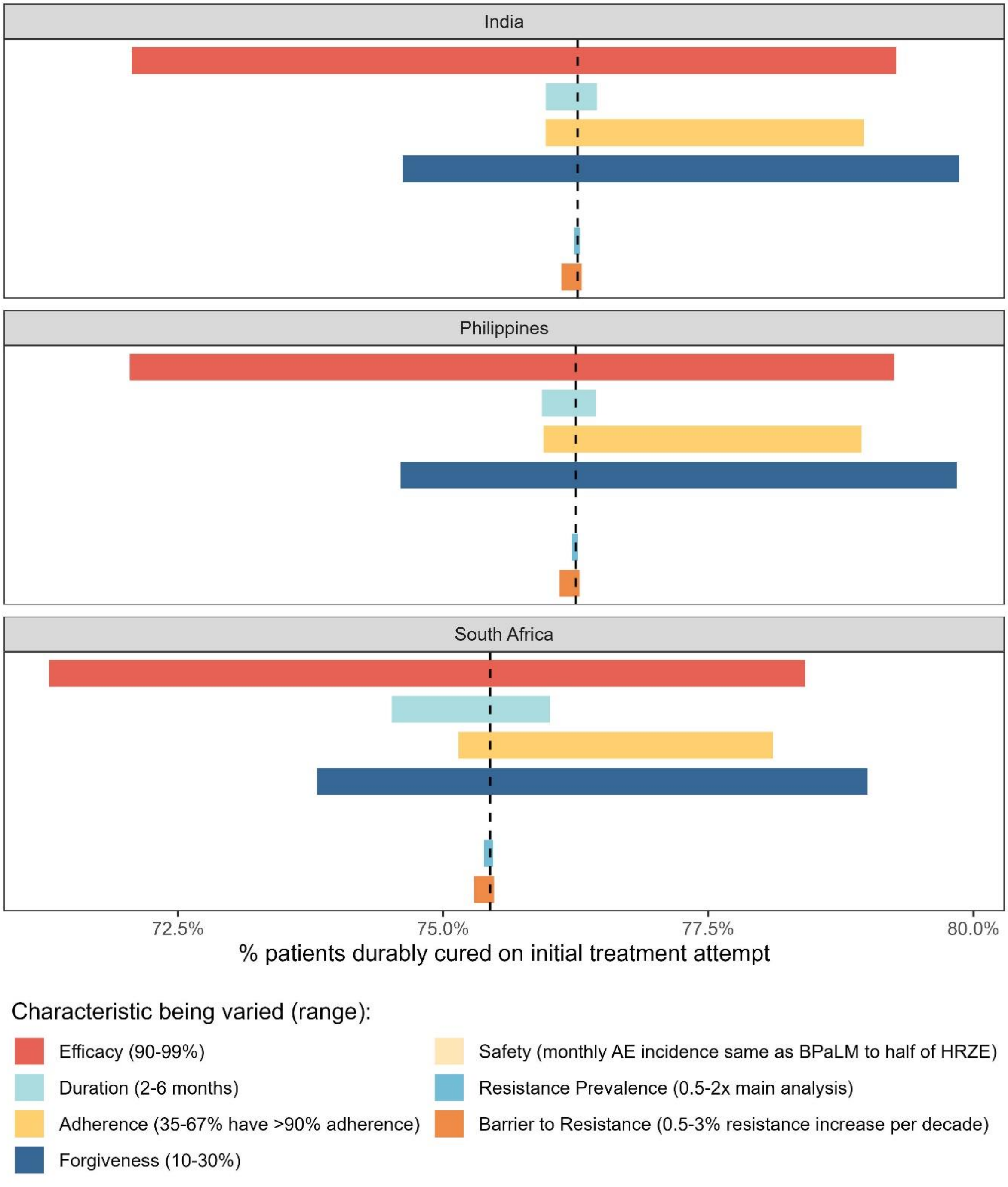

Figure shows how the proportion of patients durably cured after an initial treatment attempt with the oral Pan-TB regimen varies by that regimen's characteristics. The analysis was run with each characteristic (colors) individually varied from a pessimistic value (lefthand values of each bar) to an optimistic value (righthand values of each bar), while all other characteristics remained at their values in the main analysis. The optimistic and pessimistic values are shown in the figure legend, with additional details in Appendix Table 6. The proportion of patients durably cured in the main analysis is indicated for each country via a vertical dashed line. Durable cures were not sensitive to regimen safety (apart from its impact on adherence, which was considered as a separate characteristic), which primarily affected disability-adjusted life years and monitoring costs.

Appendix Figure 14: Effects of varying oral pan-TB regimen characteristics on TB deaths

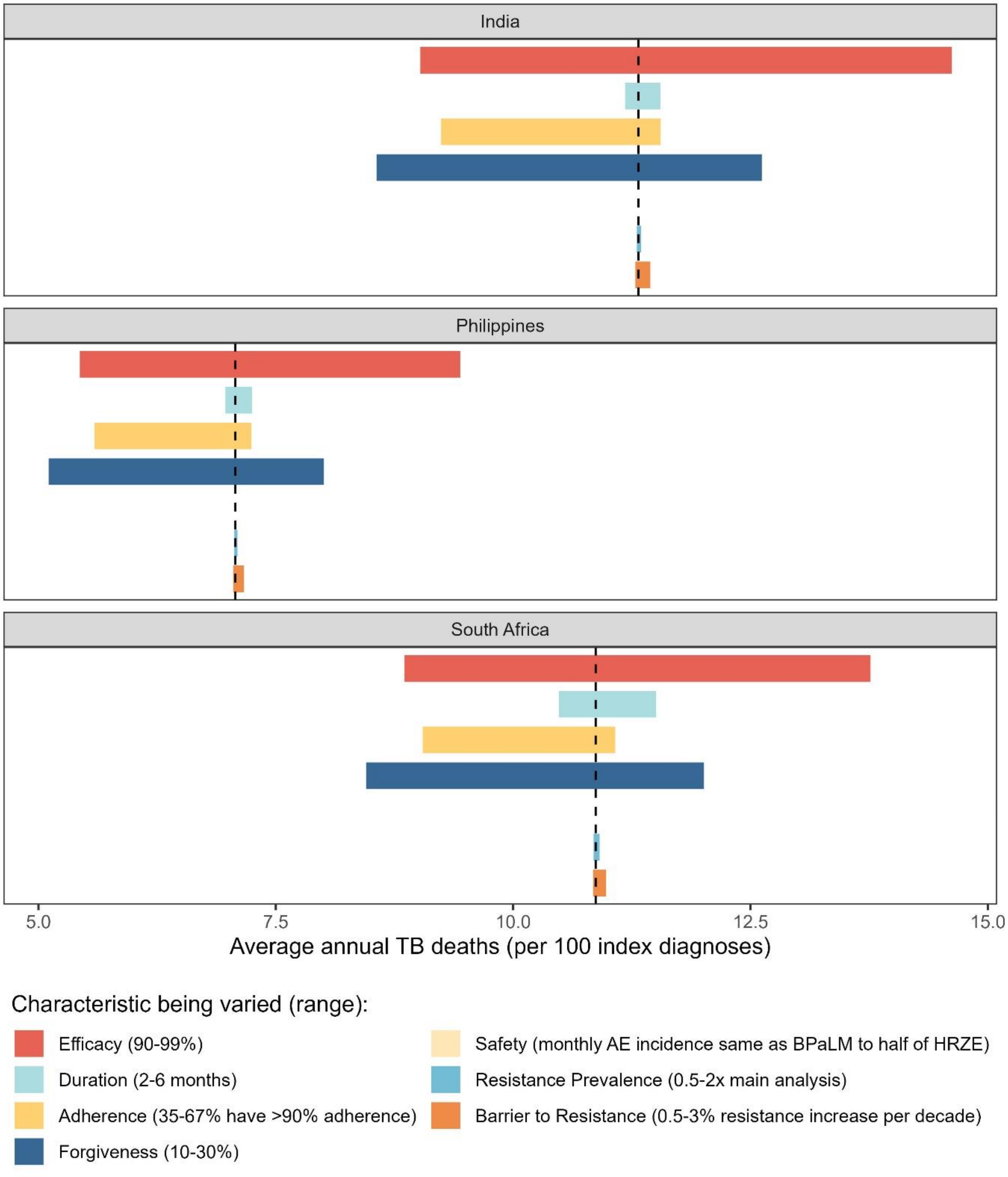

Figure shows how the number of TB deaths per 100 index patients in the oral Pan-TB regimen scenario varies by the Pan-TB regimen's characteristics. The analysis was run with each characteristic (colors) individually varied from a pessimistic value (righthand values of each bar) to an optimistic value (lefthand values of each bar), while all other characteristics remained at their values in the main analysis. The optimistic and pessimistic values are shown in the figure legend, with additional details in Appendix Table 6. The number of deaths in the main analysis is indicated for each country via a vertical dashed line. Deaths were not sensitive to regimen safety (apart from its impact on adherence, which was considered as a separate characteristic), which primarily affected disability-adjusted life years and monitoring costs.

Appendix Figure 15: Effects of varying oral pan-TB regimen characteristics on secondary cases

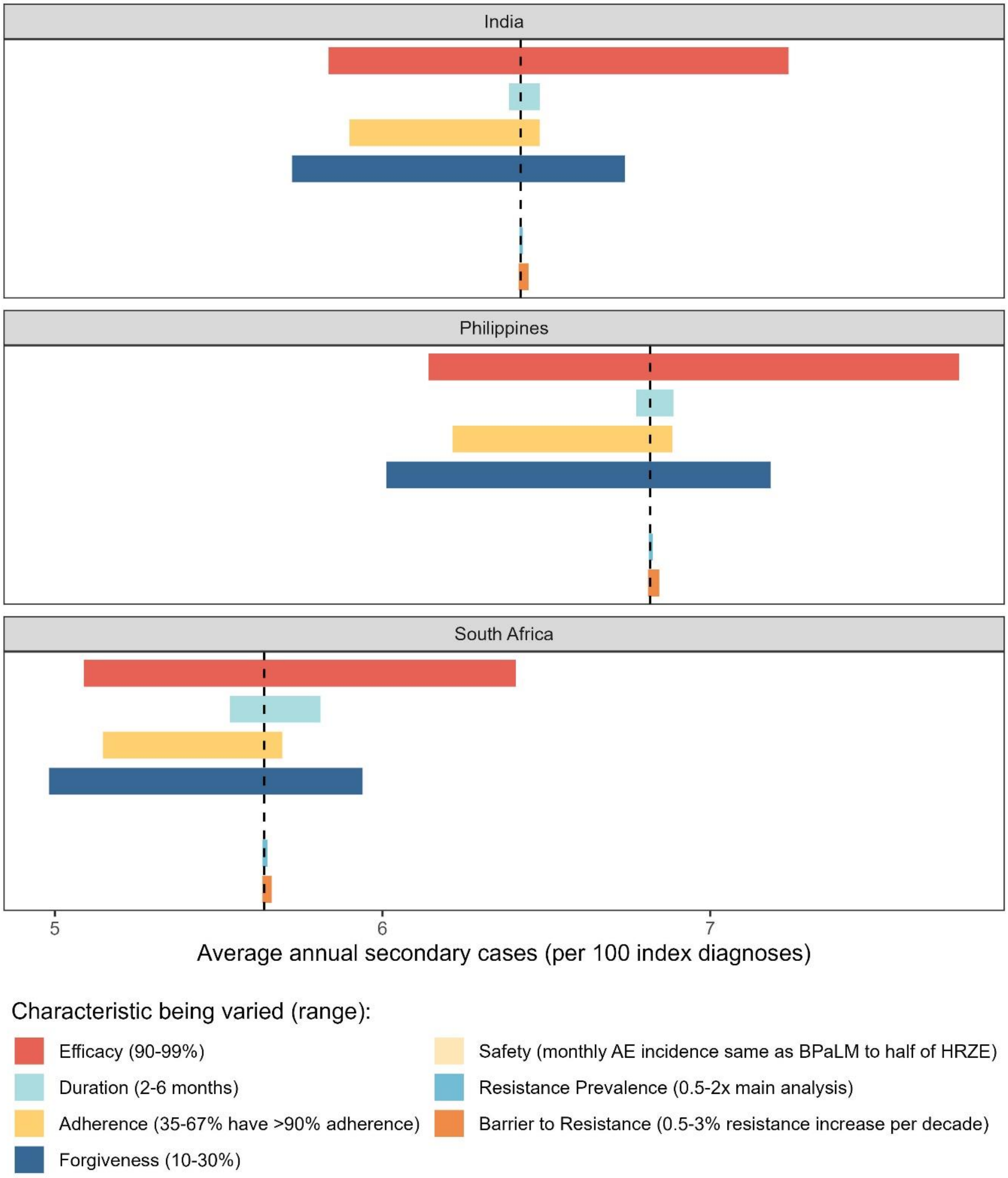

Figure shows how the number of secondary TB cases generated per 100 index patients in the oral Pan-TB regimen scenario varies by the Pan-TB regimen's characteristics. The analysis was run with each characteristic (colors) individually varied from a pessimistic value (righthand values of each bar) to an optimistic value (lefthand values of each bar), while all other characteristics remained at their values in the main analysis. The optimistic and pessimistic values are shown in the figure legend, with additional details in Appendix Table 6. The number of secondary cases in the main analysis is indicated for each country via a vertical dashed line. Secondary cases were not sensitive to regimen safety (apart from its impact on adherence, which was considered as a separate characteristic), which primarily affected disability-adjusted life years and monitoring costs.

Appendix Figure 16: Effects of varying oral pan-TB regimen characteristics on the cost-saving price thresholds

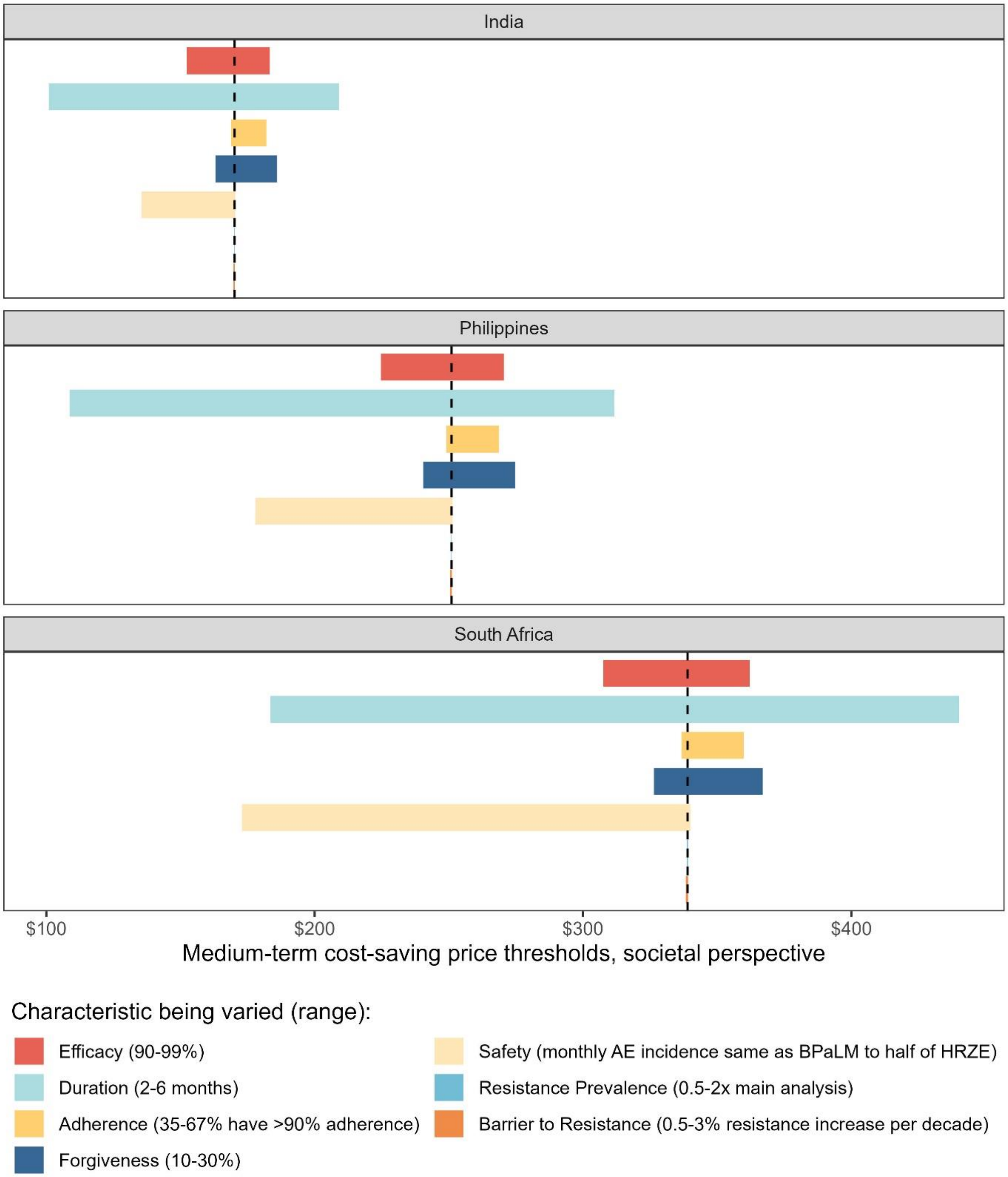

Figure shows how the medium-term cost-saving price thresholds of the oral Pan-TB regimen varied by that regimen's characteristics. The analysis considered a societal perspective that included both medical costs and patient-borne non-medical out-of-pocket and indirect costs. The analysis was run with each characteristic (colors) individually varied from a pessimistic value (lefthand values of each bar) to an optimistic value (righthand values of each bar), while all other characteristics remained at their values in the main analysis. The optimistic and pessimistic values are shown in the figure legend, with additional details in Appendix Table 6. The cost-saving price thresholds

estimated in the main analysis are indicated for each country via a vertical dashed line. Costs were relatively insensitive to resistance prevalence at baseline and the trend in the prevalence of resistance over time (labeled here as “barrier to resistance”) and thus it is difficult to see the lower blue and orange bars in each panel.

Appendix Text 5: Results of additional sensitivity analyses

Several additional sensitivity analyses were conducted to evaluate health and economic impact under alternative parameters and assumptions. A summary of the impact of these analyses on results is described below. Further results are presented in Appendix Figures 13-16 and Appendix Tables 8-11.

1. **Higher levels of resistance to novel drugs ("More novel resistance"):** In this analysis, the baseline prevalence of novel resistance (stratified by rifampin susceptibility) and the annual increase in the prevalence of novel resistance were both doubled compared to the main analysis. However, novel-resistant TB still represents a small proportion of all TB (< 3%), and thus outcomes and costs were not substantially impacted under any regimen scenario.

2. **Poorer adherence and higher levels of both pre-treatment loss-to-follow-up and discontinuation while on treatment under all scenarios ("Bigger efficacy-effectiveness gap"):** Under this analysis, the proportion of patients taking > 90% of prescribed doses decreased by 10 percentage points and the proportion taking < 70% increased by 10 percentage points under all regimens (except the Pan-TB injectable regimen, which was unaltered). Pre-treatment loss-to-follow-up and the weekly probably of discontinuation while on treatment were also increased by 50% compared to the main analysis (this latter change again affected all regimens except the Pan-TB injectable).

   Outcomes were worse under all scenarios. The oral Pan-TB regimens improved outcomes by less – primarily because there was more nonadherence and loss-to-follow-up that could not be averted via improved regimens. The injectable Pan-TB regimen also improved outcomes by less, particularly because of pre-treatment loss-to-follow-up that, again, could not be averted by improved regimens. However, the prices below which a Pan-TB regimen would be cost-saving were not substantially altered (≤ $20 difference). Of the isolated improvement scenarios, improving RR-TB retention also had a greater impact under this analysis, although it still had a much smaller impact than improving the RS-TB regimen.

3. **Better adherence and lower levels of both pre-treatment loss-to-follow-up and discontinuation while on treatment under all scenarios ("Smaller efficacy-effectiveness gap"):** This analysis was intended as a counterpart to #2 (Bigger efficacy-effectiveness gap): compared to the main analysis, the proportion of patients taking > 90% of prescribed doses *increased* by 10 percentage points, the proportion taking < 70% *decreased* by 10 percentage points, and loss-to-follow-up before and during treatment were halved. Again, the Pan-TB injectable regimen was unaltered (except for the changes to pre-treatment loss-to-follow-up).

   As expected, results were opposite those under analysis #3: outcomes were better under all scenarios, the Pan-TB regimen improved outcomes more, and changes to the cost-saving price thresholds (relative to the main analysis) were minor.

4. **Shorter RS-TB standard of care ("4-month RS-TB SOC"):** In this analysis, the RS-TB standard of care was modeled as a 4-month regimen – reducing discontinuation during treatment and also reducing inputs like outpatient visits and laboratory tests that were accrued in the cost analysis in months 5 and 6. Other regimens were unaltered. This analysis was intended to approximate a scenario in which the 4-month isoniazid rifapentine moxifloxacin pyrazinamide (4HPMZ) regimen, which had non-inferior efficacy and approximately similar safety in a clinical trial,[76] is scaled up prior to the introduction of a pan-TB regimen. The reduction in the RS-TB regimen duration slightly improved outcomes and more substantially reduced non-drug costs under the standard of care. As a result, the impact of all regimen improvements was slightly reduced and the cost-saving price thresholds of the Pan-TB regimens decreased by $10-90, depending on the country, perspective, and time horizon.

Appendix Figure 13: Proportion of patients cured with standard of care and pan-TB regimens, under additional sensitivity analyses

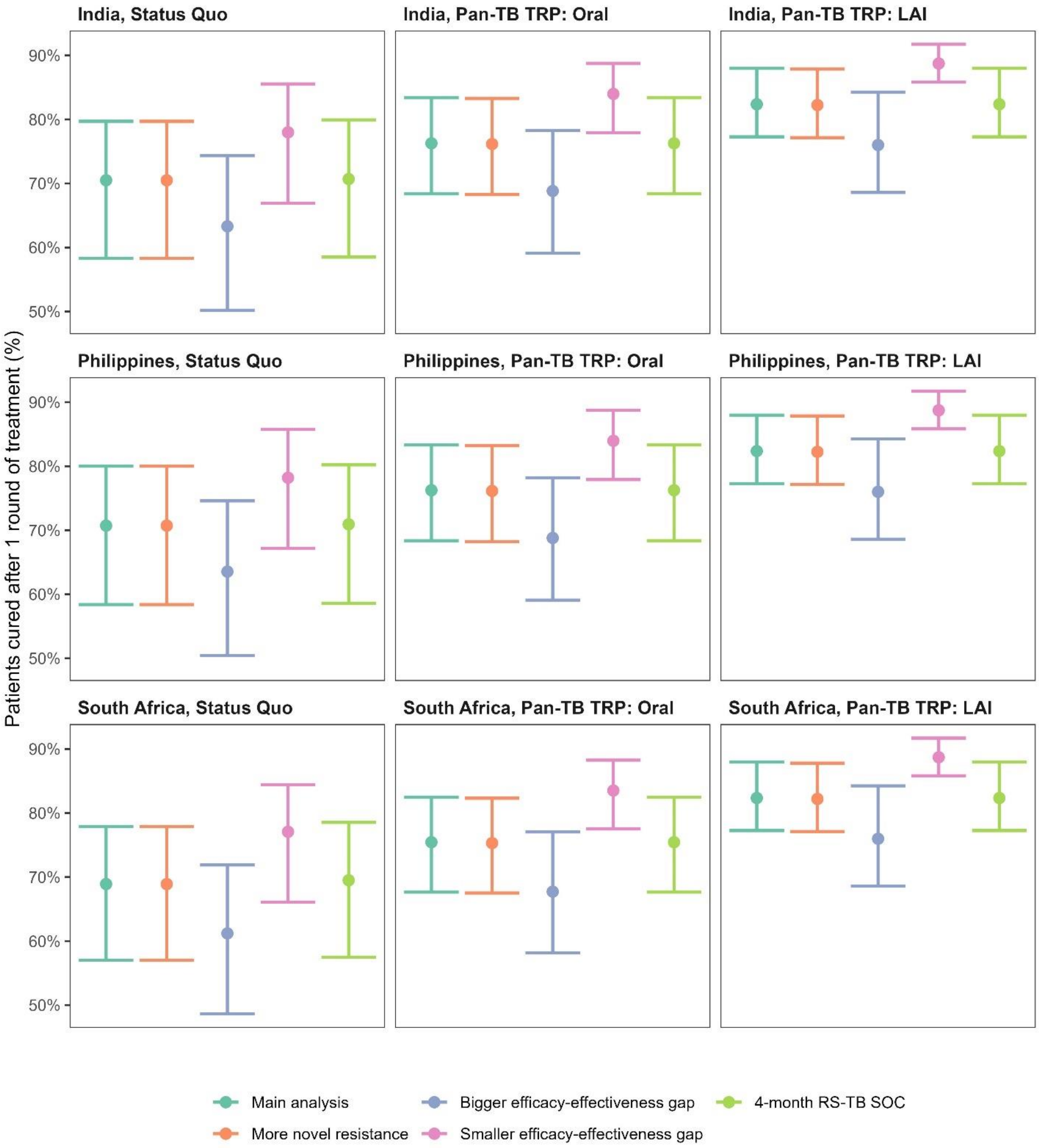

Figure shows the proportion of patients cured under each country and scenario analysis after one round of treatment, under the status quo and Pan-TB regimen scenarios. Error bars indicate 95% uncertainty intervals. Results are presented as annual averages over 10 years. “TRP” = target regimen profile; “LAI” = long-acting injectable; “RS” = rifampin-susceptible; “DST” = drug-susceptibility testing.

Appendix Figure 14: TB deaths with standard of care and pan-TB regimens, under additional sensitivity analyses

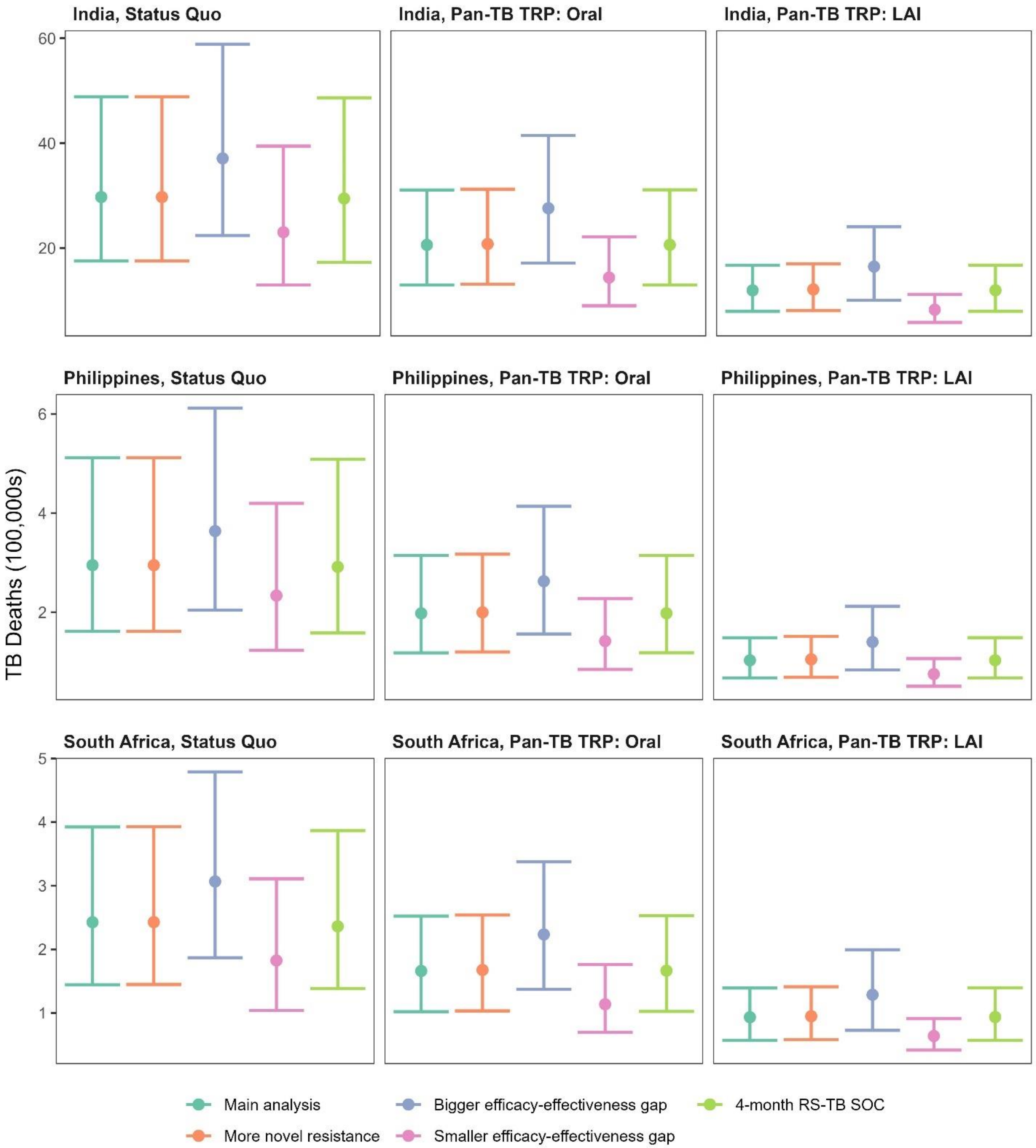

Figure shows the total number of TB deaths avertible at the point of diagnosis (including post-diagnosis deaths in the index patient and all deaths in the secondary cases they generate after diagnosis) estimated for each country and scenario analysis, under the status quo and Pan-TB regimen scenarios. Error bars indicate 95% uncertainty intervals. Deaths are presented cumulatively over 10 years. "TRP" = target regimen profile; "LAI" = long-acting injectable; "RS" = rifampin-susceptible; "DST" = drug-susceptibility testing.

Appendix Figure 15: Secondary cases with standard of care and pan-TB regimens, under additional sensitivity analyses

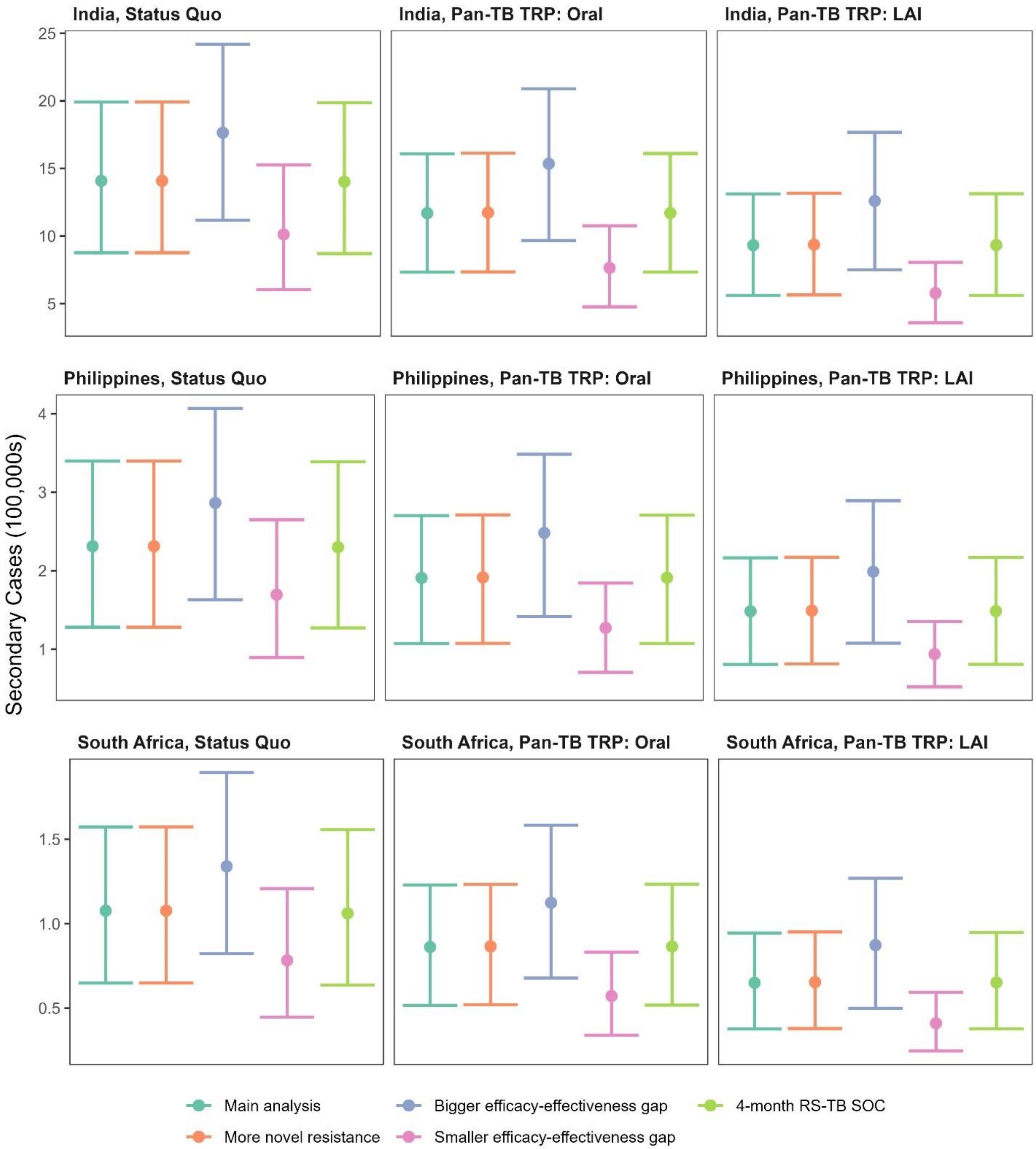

Figure shows the total number of secondary cases (occurring after diagnosis) estimated for each country and scenario analysis, under the status quo and Pan-TB regimen scenarios. Secondary cases are presented cumulatively over a 10-year time horizon. Error bars indicate 95% uncertainty intervals. "TRP" = target regimen profile; "LAI" = long-acting injectable; "RS" = rifampin-susceptible; "DST" = drug-susceptibility testing.

Appendix Figure 16: Short-term non-drug costs with standard of care and pan-TB regimens, under additional sensitivity analyses

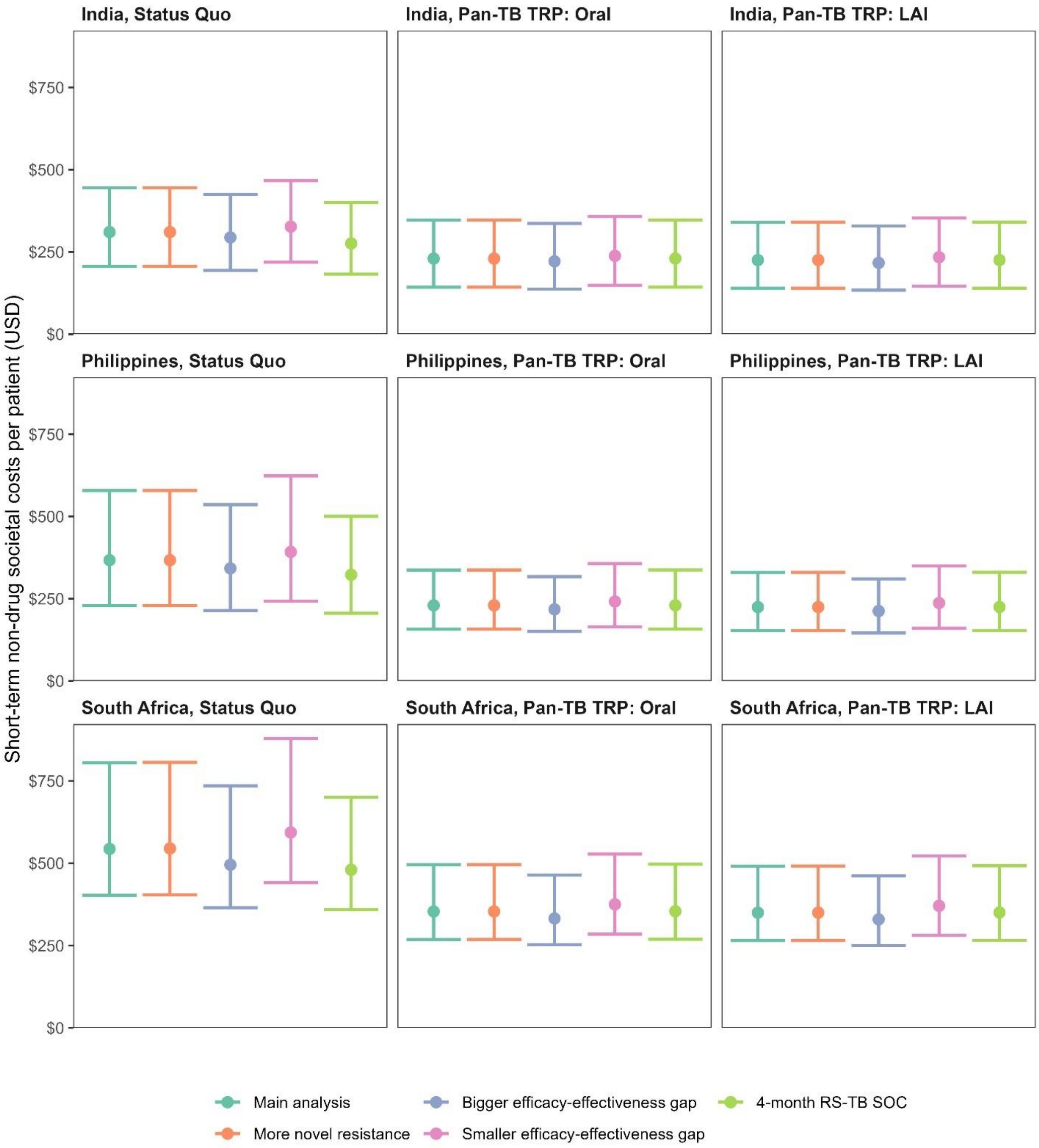

Figure shows the short-term costs per patient, under a societal perspective, estimated for each country and scenario analysis, under the status quo and Pan-TB regimen scenarios. Patient-borne out-of-pocket and non-medical costs are included, while the costs of retreatments and treatment of secondary cases are excluded. Costs are averaged over 10 years. Error bars indicate 95% uncertainty intervals. “TRP” = target regimen profile; “LAI” = long-acting injectable; “RS” = rifampin-susceptible; “DST” = drug-susceptibility testing.

Appendix Table 9: Percent decrease in proportion of patients not initially cured from regimen improvements, under additional sensitivity analyses

Regimen	Analysis	India	Philippines	South Africa
Pan-TB TRP: Oral	Main analysis	13.5% [7.7-21.1%]	12.3% [6.5-20.1%]	15.0% [9.5-22.0%]
	More novel resistance	13.3% [7.3-20.9%]	12.0% [6.1-19.8%]	14.7% [9.1-21.7%]
	Bigger efficacy-effectiveness gap	11.0% [6.2-17.6%]	10.1% [5.3-16.8%]	12.6% [8.1-18.7%]
	Smaller efficacy-effectiveness gap	17.7% [10.4-26.5%]	15.9% [8.5-25.1%]	18.9% [11.8-27.3%]
	4-month RS-TB SOC	13.1% [7.2-20.9%]	11.9% [5.9-19.8%]	13.8% [8.0-21.1%]
Pan-TB Oral, with DST	Main analysis	13.6% [7.8-21.2%]	12.3% [6.5-20.1%]	15.1% [9.7-22.0%]
	More novel resistance	13.4% [7.6-21.0%]	12.1% [6.2-19.9%]	14.9% [9.4-21.8%]
	Bigger efficacy-effectiveness gap	11.1% [6.3-17.7%]	10.1% [5.3-16.8%]	12.7% [8.2-18.7%]
	Smaller efficacy-effectiveness gap	17.9% [10.6-26.6%]	16.0% [8.6-25.1%]	19.1% [12.2-27.4%]
	4-month RS-TB SOC	13.2% [7.3-20.9%]	11.9% [6.0-19.8%]	13.9% [8.2-21.2%]
Pan-TB TRP: LAI	Main analysis	27.1% [14.8-41.8%]	25.1% [13.2-39.8%]	30.0% [18.6-43.8%]
	Pan-TB novel DST	26.8% [14.5-41.5%]	24.8% [12.8-39.5%]	29.7% [18.2-43.5%]
	Bigger efficacy-effectiveness gap	24.6% [13.0-39.4%]	23.0% [11.7-37.7%]	27.8% [17.0-41.5%]
	Smaller efficacy-effectiveness gap	31.0% [18.0-45.6%]	28.3% [15.3-43.3%]	33.4% [20.8-47.2%]
	4-month RS-TB SOC	26.8% [14.4-41.6%]	24.8% [12.7-39.7%]	29.2% [17.3-43.3%]
Improved RS Regimen	Main analysis	11.1% [4.8-19.2%]	10.6% [4.6-18.6%]	11.9% [6.2-19.4%]
	More novel resistance	10.8% [4.4-18.9%]	10.3% [4.2-18.4%]	11.6% [5.8-19.2%]
	Bigger efficacy-effectiveness gap	8.9% [3.9-15.9%]	8.7% [3.7-15.5%]	9.9% [5.3-16.3%]
	Smaller efficacy-effectiveness gap	14.5% [6.5-24.1%]	13.7% [6.0-23.4%]	15.1% [7.5-24.2%]
	4-month RS-TB SOC	10.7% [4.3-18.9%]	10.2% [4.0-18.3%]	10.7% [4.6-18.6%]
Improved RR Regimen	Main analysis	0.2% [0.1- 0.5%]	0.1% [0.0- 0.3%]	0.5% [0.2- 0.9%]
	More novel resistance	0.2% [0.1- 0.5%]	0.1% [0.0- 0.3%]	0.5% [0.2- 0.9%]
	Bigger efficacy-effectiveness gap	0.2% [0.1- 0.3%]	0.1% [0.0- 0.2%]	0.4% [0.2- 0.6%]
	Smaller efficacy-effectiveness gap	0.4% [0.2- 0.7%]	0.2% [0.1- 0.4%]	0.8% [0.3- 1.4%]
	4-month RS-TB SOC	0.3% [0.1- 0.5%]	0.1% [0.0- 0.3%]	0.5% [0.2- 0.9%]
Improved RR Retention	Main analysis	0.3% [0.1- 0.7%]	0.2% [0.1- 0.4%]	0.8% [0.3- 1.5%]
	More novel resistance	0.3% [0.1- 0.7%]	0.2% [0.1- 0.4%]	0.8% [0.3- 1.5%]
	Bigger efficacy-effectiveness gap	0.4% [0.2- 0.9%]	0.2% [0.1- 0.5%]	0.9% [0.4- 1.8%]
	Smaller efficacy-effectiveness gap	0.2% [0.1- 0.4%]	0.1% [0.0- 0.3%]	0.5% [0.2- 1.0%]
	4-month RS-TB SOC	0.3% [0.1- 0.7%]	0.2% [0.1- 0.4%]	0.8% [0.3- 1.5%]
Improved RR Assignment	Main analysis	1.1% [0.5- 1.9%]	0.8% [0.4- 1.5%]	0.8% [0.3- 1.6%]
	More novel resistance	1.1% [0.5- 1.9%]	0.8% [0.4- 1.5%]	0.8% [0.3- 1.6%]
	Bigger efficacy-effectiveness gap	0.7% [0.2- 1.3%]	0.5% [0.2- 1.0%]	0.5% [0.1- 1.0%]
	Smaller efficacy-effectiveness gap	1.7% [0.8- 3.0%]	1.3% [0.6- 2.4%]	1.4% [0.5- 2.7%]
	4-month RS-TB SOC	1.1% [0.5- 1.9%]	0.8% [0.4- 1.5%]	0.8% [0.3- 1.6%]

Table shows, under the Pan-TB and isolated improvement regimen scenarios and under all scenario analyses, the percent declines in the proportion of newly diagnosed TB patients not cured after 1 round of treatment relative to the standard of care scenario. Reductions in non-cures are presented cumulatively over 10 years. 95% uncertainty intervals are shown in brackets. “TRP” = target regimen profile; “LAI” = long-acting injectable; “RS” = rifampin-susceptible; “RR” = rifampin-resistant; “DST” = drug-susceptibility testing.

Appendix Table 10: Percent decline in TB deaths from regimen improvements, under additional sensitivity analyses

Regimen	Analysis	India	Philippines	South Africa
Pan-TB TRP: Oral	Main analysis	29.8% [19.9-40.9%]	31.8% [20.9-43.2%]	30.9% [22.0-41.0%]
	More novel resistance	29.1% [18.8-40.5%]	31.1% [19.7-42.8%]	30.3% [20.9-40.5%]
	Bigger efficacy-effectiveness gap	25.0% [16.0-35.4%]	27.0% [17.2-37.5%]	26.8% [18.9-36.0%]
	Smaller efficacy-effectiveness gap	36.3% [24.8-48.3%]	37.8% [25.4-50.3%]	36.7% [26.0-48.1%]
	4-month RS-TB SOC	29.0% [18.7-40.4%]	30.9% [19.5-42.8%]	28.7% [18.9-39.6%]
Pan-TB Oral, with DST	Main analysis	30.0% [20.2-40.9%]	31.9% [21.1-43.2%]	31.1% [22.4-41.1%]
	More novel resistance	29.5% [19.6-40.6%]	31.2% [20.0-42.8%]	30.7% [21.9-40.7%]
	Bigger efficacy-effectiveness gap	25.1% [16.3-35.4%]	27.0% [17.3-37.5%]	27.0% [19.2-36.1%]
	Smaller efficacy-effectiveness gap	36.5% [25.3-48.4%]	37.9% [25.6-50.3%]	37.0% [26.6-48.2%]
	4-month RS-TB SOC	29.2% [19.0-40.5%]	30.9% [19.6-42.8%]	28.9% [19.4-39.7%]
Pan-TB TRP: LAI	Main analysis	57.9% [37.9-76.4%]	62.9% [42.4-80.2%]	60.2% [42.5-77.1%]
	More novel resistance	57.3% [37.2-76.0%]	62.2% [41.4-79.9%]	59.5% [41.7-76.6%]
	Bigger efficacy-effectiveness gap	54.0% [33.8-74.2%]	59.6% [38.2-78.5%]	57.1% [39.1-75.3%]
	Smaller efficacy-effectiveness gap	62.0% [42.8-78.8%]	65.4% [45.5-81.5%]	63.2% [45.7-79.1%]
	4-month RS-TB SOC	57.4% [37.1-76.2%]	62.3% [41.4-80.1%]	58.9% [40.3-76.6%]
Improved RS Regimen	Main analysis	24.0% [12.1-37.0%]	27.0% [14.5-40.1%]	24.3% [14.0-36.2%]
	More novel resistance	23.3% [11.0-36.6%]	26.3% [13.1-39.8%]	23.8% [13.1-35.8%]
	Bigger efficacy-effectiveness gap	20.0% [10.0-31.7%]	22.9% [12.2-34.8%]	20.9% [12.3-31.3%]
	Smaller efficacy-effectiveness gap	29.3% [15.1-44.0%]	32.0% [17.3-46.8%]	29.0% [16.0-42.8%]
	4-month RS-TB SOC	23.1% [10.8-36.5%]	26.0% [12.9-39.7%]	21.9% [10.5-34.9%]
Improved RR Regimen	Main analysis	0.7% [0.3- 1.2%]	0.4% [0.2- 0.8%]	1.3% [0.6- 2.3%]
	More novel resistance	0.6% [0.3- 1.2%]	0.4% [0.2- 0.8%]	1.3% [0.6- 2.2%]
	Bigger efficacy-effectiveness gap	0.5% [0.2- 0.9%]	0.3% [0.1- 0.6%]	0.9% [0.4- 1.6%]
	Smaller efficacy-effectiveness gap	0.9% [0.4- 1.7%]	0.6% [0.2- 1.2%]	1.8% [0.8- 3.2%]
	4-month RS-TB SOC	0.7% [0.3- 1.2%]	0.4% [0.2- 0.8%]	1.3% [0.6- 2.3%]
Improved RR Retention	Main analysis	0.9% [0.3- 1.9%]	0.5% [0.1- 1.1%]	1.6% [0.6- 3.1%]
	More novel resistance	0.9% [0.3- 1.8%]	0.5% [0.1- 1.1%]	1.5% [0.6- 3.1%]
	Bigger efficacy-effectiveness gap	1.2% [0.4- 2.4%]	0.6% [0.2- 1.5%]	2.0% [0.7- 4.1%]
	Smaller efficacy-effectiveness gap	0.5% [0.2- 1.1%]	0.3% [0.1- 0.6%]	0.9% [0.3- 1.9%]
	4-month RS-TB SOC	0.9% [0.3- 1.9%]	0.5% [0.1- 1.1%]	1.6% [0.6- 3.2%]
Improved RR Assignment	Main analysis	2.8% [1.2- 5.1%]	2.7% [1.1- 5.2%]	2.1% [0.7- 4.2%]
	More novel resistance	2.7% [1.2- 5.1%]	2.7% [1.1- 5.1%]	2.1% [0.7- 4.1%]
	Bigger efficacy-effectiveness gap	1.7% [0.6- 3.5%]	1.8% [0.7- 3.7%]	1.3% [0.4- 2.8%]
	Smaller efficacy-effectiveness gap	4.1% [1.8- 7.5%]	3.9% [1.6- 7.2%]	3.2% [1.1- 6.3%]
	4-month RS-TB SOC	2.8% [1.2- 5.2%]	2.8% [1.1- 5.2%]	2.1% [0.7- 4.3%]

Table shows, under the Pan-TB and isolated improvement regimen scenarios and under all scenario analyses, the percent change in TB deaths that could be averted at the point of diagnosis (including post-diagnosis deaths in the index patient and all deaths in the secondary cases they generate after diagnosis) relative to the standard of care scenario. Reductions in deaths are presented cumulatively over 10 years. 95% uncertainty intervals are shown in brackets. “TRP” = target regimen profile; “LAI” = long-acting injectable; “RS” = rifampin-susceptible; “RR” = rifampin-resistant; “DST” = drug-susceptibility testing.

Appendix Table 11: Percent decline in secondary cases from regimen improvements, under additional sensitivity analyses

Regimen	Analysis	India	Philippines	South Africa
Pan-TB TRP: Oral	Main analysis	16.7% [9.1-26.9%]	17.1% [9.0-27.6%]	19.7% [12.4-29.1%]
	More novel resistance	16.4% [8.7-26.7%]	16.8% [8.6-27.4%]	19.4% [12.0-28.8%]
	Bigger efficacy-effectiveness gap	12.9% [6.9-21.6%]	13.2% [6.7-22.2%]	16.0% [10.1-24.1%]
	Smaller efficacy-effectiveness gap	23.8% [13.6-35.8%]	24.2% [13.5-36.5%]	26.3% [16.8-37.3%]
	4-month RS-TB SOC	16.2% [8.5-26.5%]	16.5% [8.3-27.2%]	18.2% [10.7-28.0%]
Pan-TB Oral, with DST	Main analysis	16.8% [9.1-26.9%]	17.1% [9.1-27.6%]	19.8% [12.5-29.1%]
	More novel resistance	16.5% [8.9-26.7%]	16.8% [8.7-27.4%]	19.5% [12.3-28.9%]
	Bigger efficacy-effectiveness gap	12.9% [6.9-21.7%]	13.2% [6.7-22.2%]	16.1% [10.1-24.1%]
	Smaller efficacy-effectiveness gap	23.9% [13.8-35.8%]	24.3% [13.6-36.5%]	26.4% [17.1-37.4%]
	4-month RS-TB SOC	16.3% [8.6-26.5%]	16.5% [8.3-27.2%]	18.3% [10.8-28.1%]
Pan-TB TRP: LAI	Main analysis	33.2% [17.3-53.5%]	35.0% [18.2-55.6%]	39.0% [23.8-57.9%]
	More novel resistance	32.9% [17.0-53.1%]	34.7% [17.9-55.3%]	38.7% [23.5-57.6%]
	Bigger efficacy-effectiveness gap	28.4% [14.2-48.3%]	30.2% [15.0-50.6%]	34.5% [20.5-53.3%]
	Smaller efficacy-effectiveness gap	41.7% [23.5-61.6%]	43.3% [24.4-63.2%]	46.3% [29.3-64.6%]
	4-month RS-TB SOC	32.8% [16.8-53.3%]	34.6% [17.6-55.4%]	37.9% [22.4-57.2%]
Improved RS Regimen	Main analysis	13.3% [5.5-23.9%]	14.7% [6.4-25.6%]	15.1% [7.7-25.0%]
	More novel resistance	13.0% [5.1-23.7%]	14.4% [5.9-25.3%]	14.8% [7.3-24.7%]
	Bigger efficacy-effectiveness gap	10.1% [4.1-19.0%]	11.2% [4.7-20.4%]	12.0% [6.2-20.2%]
	Smaller efficacy-effectiveness gap	19.3% [8.4-32.2%]	20.9% [9.5-34.2%]	20.7% [10.4-32.9%]
	4-month RS-TB SOC	12.8% [4.9-23.6%]	14.1% [5.6-25.2%]	13.5% [5.7-23.9%]
Improved RR Regimen	Main analysis	0.3% [0.1- 0.5%]	0.2% [0.1- 0.3%]	0.6% [0.3- 1.1%]
	More novel resistance	0.3% [0.1- 0.5%]	0.2% [0.1- 0.3%]	0.6% [0.3- 1.1%]
	Bigger efficacy-effectiveness gap	0.2% [0.1- 0.4%]	0.1% [0.0- 0.2%]	0.4% [0.2- 0.8%]
	Smaller efficacy-effectiveness gap	0.5% [0.2- 0.9%]	0.3% [0.1- 0.5%]	1.0% [0.5- 1.8%]
	4-month RS-TB SOC	0.3% [0.1- 0.5%]	0.2% [0.1- 0.3%]	0.7% [0.3- 1.1%]
Improved RR Retention	Main analysis	1.0% [0.4- 1.9%]	0.5% [0.2- 1.0%]	1.7% [0.7- 3.2%]
	More novel resistance	1.0% [0.4- 1.9%]	0.5% [0.2- 1.0%]	1.7% [0.7- 3.2%]
	Bigger efficacy-effectiveness gap	1.1% [0.4- 2.2%]	0.6% [0.2- 1.2%]	2.0% [0.8- 3.7%]
	Smaller efficacy-effectiveness gap	0.7% [0.3- 1.4%]	0.3% [0.1- 0.7%]	1.2% [0.5- 2.4%]
	4-month RS-TB SOC	1.0% [0.4- 1.9%]	0.5% [0.2- 1.0%]	1.7% [0.7- 3.3%]
Improved RR Assignment	Main analysis	0.7% [0.0- 1.7%]	0.7% [0.1- 1.5%]	0.7% [0.1- 1.6%]
	More novel resistance	0.7% [0.0- 1.6%]	0.7% [0.1- 1.5%]	0.7% [0.1- 1.6%]
	Bigger efficacy-effectiveness gap	0.1% [-0.6- 0.9%]	0.2% [-0.3- 0.9%]	0.2% [-0.3- 0.9%]
	Smaller efficacy-effectiveness gap	1.8% [0.7- 3.3%]	1.6% [0.7- 2.9%]	1.6% [0.6- 3.2%]
	4-month RS-TB SOC	0.7% [0.0- 1.7%]	0.7% [0.1- 1.5%]	0.7% [0.1- 1.7%]

Table shows, under the Pan-TB and isolated improvement regimen scenarios and under all scenario analyses, the percent changes in total secondary cases (occurring after diagnosis) relative to the standard of care scenario. Reductions in secondary cases are presented cumulatively over 10 years. 95% uncertainty intervals are shown in brackets. “TRP” = target regimen profile; “LAI” = long-acting injectable; “RS” = rifampin-susceptible; “RR” = rifampin-resistant; “DST” = drug-susceptibility testing.

Appendix Table 12: Cost-saving price thresholds, under additional sensitivity analyses

Regimen	Analysis	India	Philippines	South Africa
Short-Term, Health Systems Perspective				
Pan-TB TRP: Oral	Main analysis	90 [80-100]	70 [70-80]	150 [130-170]
	More novel resistance	90 [80-100]	70 [70-80]	150 [130-170]
	Bigger efficacy-effectiveness gap	90 [80-100]	70 [70-80]	140 [120-160]
	Smaller efficacy-effectiveness gap	90 [80-100]	80 [70-80]	160 [130-180]
	4-month RS-TB SOC	80 [70-90]	70 [60-70]	120 [110-150]
Pan-TB TRP: LAI	Main analysis	90 [80-100]	80 [70-90]	150 [130-180]
	More novel resistance	90 [80-100]	80 [70-90]	150 [130-180]
	Bigger efficacy-effectiveness gap	90 [80-100]	80 [70-90]	140 [120-170]
	Smaller efficacy-effectiveness gap	90 [80-100]	80 [70-90]	160 [140-190]
	4-month RS-TB SOC	80 [70-90]	70 [60-80]	130 [110-150]
Medium-Term, Health Systems Perspective				
Pan-TB TRP: Oral	Main analysis	140 [110-190]	190 [130-290]	280 [210-400]
	More novel resistance	140 [110-190]	190 [130-290]	280 [210-400]
	Bigger efficacy-effectiveness gap	140 [110-180]	180 [120-280]	260 [190-370]
	Smaller efficacy-effectiveness gap	150 [110-190]	200 [130-310]	300 [230-430]
	4-month RS-TB SOC	110 [90-130]	150 [100-220]	210 [160-290]
Pan-TB TRP: LAI	Main analysis	160 [120-210]	210 [140-330]	310 [230-440]
	More novel resistance	160 [120-210]	210 [140-330]	310 [230-440]
	Bigger efficacy-effectiveness gap	150 [120-200]	210 [140-320]	280 [210-410]
	Smaller efficacy-effectiveness gap	160 [120-210]	220 [140-340]	320 [240-470]
	4-month RS-TB SOC	120 [100-150]	170 [110-250]	230 [170-330]
Short-Term, Societal Perspective				
Pan-TB TRP: Oral	Main analysis	140 [110-190]	210 [130-330]	270 [200-410]
	More novel resistance	140 [110-190]	210 [130-330]	270 [200-420]
	Bigger efficacy-effectiveness gap	140 [100-190]	200 [130-320]	260 [180-390]
	Smaller efficacy-effectiveness gap	140 [110-190]	210 [130-330]	290 [210-430]
	4-month RS-TB SOC	100 [90-120]	160 [100-240]	200 [150-290]
Pan-TB TRP: LAI	Main analysis	150 [110-200]	220 [130-350]	280 [200-430]
	More novel resistance	150 [110-200]	220 [130-350]	290 [210-430]
	Bigger efficacy-effectiveness gap	150 [110-200]	210 [130-340]	270 [190-410]
	Smaller efficacy-effectiveness gap	150 [110-200]	220 [140-350]	300 [220-450]
	4-month RS-TB SOC	110 [90-130]	170 [110-250]	210 [150-310]
Medium-Term, Societal Perspective				
Pan-TB TRP: Oral	Main analysis	170 [130-230]	250 [150-400]	340 [250-510]
	More novel resistance	170 [130-230]	250 [150-400]	340 [250-510]
	Bigger efficacy-effectiveness gap	170 [130-230]	250 [150-410]	330 [230-490]
	Smaller efficacy-effectiveness gap	170 [130-230]	250 [150-400]	350 [250-520]
	4-month RS-TB SOC	130 [100-160]	190 [130-300]	260 [190-370]
Pan-TB TRP: LAI	Main analysis	200 [140-280]	290 [170-480]	390 [270-600]
	More novel resistance	200 [140-280]	290 [170-480]	390 [270-600]
	Bigger efficacy-effectiveness gap	200 [140-290]	300 [180-500]	380 [260-590]
	Smaller efficacy-effectiveness gap	190 [140-270]	280 [170-460]	390 [270-590]
	4-month RS-TB SOC	150 [110-220]	230 [140-380]	300 [210-460]

Table shows cost-saving price thresholds of the Pan-TB all-oral 3·5-month regimen and the Pan-TB long-acting injectable (LAI), under different horizons (short-term incorporating costs during a patient's treatment course only or medium-term incorporating averted costs of retreatments and secondary case treatments over 10 years), and perspectives (health systems perspective that omits patient-borne non-medical costs or societal perspective that includes them). All prices are presented in 2021 USD. 95% uncertainty intervals are shown in brackets. "TRP" = target regimen profile; "LAI" = long-acting injectable; "RS" = rifampin-susceptible; "RR" = rifampin-resistant; "DST" = drug-susceptibility testing.
